# *De novo* transcriptome of the diatom *Cylindrotheca closterium* identifies genes involved in the metabolism of anti-inflammatory compounds

**DOI:** 10.1038/s41598-020-61007-0

**Published:** 2020-03-05

**Authors:** Ali M. Elagoz, Luca Ambrosino, Chiara Lauritano

**Affiliations:** 1 Department of Integrative Marine Ecology, Stazione Zoologica Anton Dohrn, Villa Comunale, 80121, Napoli, Italy; 2 Ghent University, Marine Biology Research Group, Krijgslaan 281, B-9000 Gent, Belgium; 3 Research Infrastructure for Marine Biological Resources Department, Stazione Zoologica Anton Dohrn, Villa Comunale, 80121, Napoli, Italy

**Keywords:** Plant biotechnology, Microbial ecology, Molecular ecology, Transcriptomics

## Abstract

Diatoms are the most diverse and abundant group of phytoplankton species and represent a huge reservoir of marine natural products with possible application for human health. Several diatoms are known to have anticancer, anti-inflammatory, antioxidant and anti-microbial properties, but the compounds responsible of these activities are often still unknown. The diatom *Cylindrotheca closterium* showed anti-inflammatory properties inhibiting TNFα release in human monocytic leukemia cells. In this study, we present the full transcriptome of *C. closterium*, and used an -omic approach to identify transcripts coding enzymes that can be involved in the synthesis/degradation of anti-inflammatory compounds. This approach allowed to identify phosphatidylinositol-3-phosphatase, phosphatidylinositol 3-kinase catalytic subunit type 3, phosphatidylinositol N-acetylglucosaminyltransferase subunit A, monogalactosyldiacylglycerol synthase and violaxanthin de-epoxidase, which are known to be involved in anti-inflammatory compound metabolism. When *C. closterium* was cultured in silica-starvation conditions, selected as stress condition to potentially trigger the synthesis of bioactive metabolites, anti-inflammatory activity was lost and expression levels of the analyzed transcripts were reduced. These data suggested that the control culturing condition was the most active. This study used for the first time a transcriptomic-guided approach to identify enzymes involved in anti-inflammatory compound metabolism, directing future discoveries of marine natural products in microalgae.

## Introduction

Diatoms are the most diverse group of phytoplankton with an estimation of 200,000 different species^1^. Their diversity offers an enormous reservoir of new compounds with possible biotechnological applications^2^. Throughout the last decades, enhancement in the multi-omics methodologies (such as genomics, transcriptomics, proteomics and metabolomics) lead to exploration and exploitation of diatom properties, supporting the identification and characterization of new marine natural products and enzymes with possible applications in the blue biotechnology field^2–6^. Diatoms have been already shown to have antioxidant^7^, anti-diabetes^8^, anticancer^9^, antibacterial^7,10^, anti-tuberculosis^11^, anti-epilepsy^12^ and anti-inflammatory^7,10,13^ properties.

Recently, various studies have been carried out for identification of microalgal compounds with anti-inflammatory activities for human applications (e.g. drugs or nutraceuticals^14^). The most known microalgal compounds with anti-inflammatory properties are various carotenoids, polyunsaturated fatty acids (PUFA) and certain carbohydrates^15–17^. In addition, other studies have reported microalgal anti-inflammatory properties but the bioactive chemical entities are still unknown^7,10^.

Currently, six diatoms showed anti-inflammatory properties: *Porosira glacialis*, *Attleya longicornis*^7^, *Cylindrotheca closterium*, *Odontella mobiliensis*, *Pseudonitzschia pseudodelicatissima*^10^ and *Phaeodactylum tricornutum*^14,18^. Both Lauritano *et al*.^10^ and Ingebrigtsen *et al*.^7^ carried out an assessment of the capability of inhibition of the tumour necrosis factor α release, one of the fundamental mediators of inflammation^19^, in lipopolysaccharide-stimulated human monocytic leukaemia cells (THP-1). Samarakoon *et al*.^18^ evaluated the inhibition of NO production (%) level, another inflammatory mediator, in LPS-induced RAW macrophages. Even though these approaches allowed to identify diatoms with anti-inflammatory properties, they did not reveal yet the microalgal bioactive metabolite(s).

In this study, a transcriptomic approach has been used in order to identify transcripts that can be involved in the synthesis/degradation of compounds with anti-inflammatory properties in the diatom *Cylindrotheca closterium*. In particular, we sequenced the transcriptome of *C. closterium* in both control (complete medium) and stress conditions (Silica-starvation). Nitrogen- and phosphate-starvation have already shown to reduce the anti-inflammatory bioactivity of *C. closterium*^10^. The reason behind stressing the microalgae is to trigger the activation of the broadest range of metabolic pathways of interest, which can be silent under control conditions. Diatoms require silica (Si) to build the frustules that surround the cell and we hypothesized that Si-starvation could induce a stress response in the cell with possible activation of metabolic pathways of interest. Si is also known to be important for transporting gases and solutes, light penetration and defence against predators. For many diatoms, Si-depletion is known to induce cell cycle and growth arrest^20–23^. Therefore, Si is an important limiting factor for diatom productivity and may lead to alterations in cellular homeostasis. In this study, Si-starvation was chosen rather than complete Si-depletion in order to avoid cell death and, at the same time, inducing a less severe stress exposure. Previous studies on various microalgae have shown that Si-starvation/depletion induced a metabolic remodelling, including the activation of specific pathways involved in the synthesis of potential bioactive metabolites^24,25^.

Among microalgal compounds, phosphatidylinositol, monogalactosyldiacylglycerols and various carotenoids are known to have anti-inflammatory properties^26–30^ and we looked for enzymes involved in their synthesis/degradation. This is the first study investigating transcripts involved in the metabolism of anti-inflammatory metabolites in *C. closterium*, showing that they are differentially expressed under stress exposure and providing information directing the chemical discovery of new marine natural products in diatoms.

## Results and Discussion

### Transcriptome sequencing and de novo assembly

RNA sequencing (RNA-seq) experiment was performed on 6 samples: a triplicate derived from the microalga *C. closterium* cultured in complete medium and considered as control condition (CTRL), and a triplicate of *C. closterium* cultured in Si-starvation conditions (Si-starved). Since the genome of *C. closterium* is not available, RNA-seq reads have been assembled with *de novo* approach producing 44718 putative transcripts grouped into 33433 genes. The mean GC content was 46.16%. The average and the median contig length were 1064 bp and 781 bp, respectively. The N50 was 1554 bp.

Several controls were performed on the raw transcriptome assembly to check for its quality. Transcripts were translated into proteins obtaining a total of 32586 protein sequences (minimum length 50aa). Among those, 14307 (43.90%) were complete (with a methionine and a stop codon), 2855 (8.76%) started with a methionine but lacked a stop codon, 8194 (25.14%) only had a stop codon, 7230 (22.18%) did not start with a methionine and did not have a stop codon.

In order to verify the completeness of the assembly, the protein sequences were blasted against the two datasets of the Core Eukaryotic Genes, including 248 (http://korflab.ucdavis.edu/Datasets/genome_completeness/) and 458 (http://korflab.ucdavis.edu/datasets/cegma/) protein sequences, respectively. 248 out of 248 (100%) and 456 out of 458 (99.56%) could be detected in the assembly. In addition, the length of the proteins from the assembly was compared to the length of the core eukaryotic genes. About 403 of the proteins covered more than 90% of the length of the corresponding core eukaryotic proteins and 536 covered more than 80% of the length of the corresponding core eukaryotic protein, indicating a good quality of the assembly.

As NGS data could suffer from contaminations of organisms that are not the target of the experiment, we blasted the sequences of the transcripts against the NCBI database of bacteria and archaea in order to remove possible contaminations. By this way, 172 transcripts were detected and removed. In order to detect other sources of contamination, the distribution of the GC content was analysed in the dataset. As already mentioned, the GC content followed a normal distribution with a mean value of 45.84% and a standard deviation of 3.48. Following a z‐test, 5445 sequences could be identified for having a GC content significantly different from the observed mean (p < 0.01). Those sequences were blasted against the NCBI (nr) database to look for contaminants. About 878 sequences were removed for matching bacterial or metazoan sequences.

The obtained assembly was initially composed by 43668 transcripts belonging to 32606 genes. The mean GC content was 46.11%. The average and the median contig length were 1079.06 bp and 803 bp, respectively. The N50 was 1564 bp.

### Functional annotation

Assembled transcript sequences were translated into proteins with Transdecoder (minimum length of 50 aa). When multiple translations were possible, the priority was set in order to get the longest complete ORF; when a complete ORF was not detected the longest sequence was kept. The sequences were also investigated for the presence of repetitive elements with Repeat Masker. The software Blast2GO was used to associate a function to the assembled transcripts. A total of 20540 proteins got significant blast hits, and among these 11178 proteins had gene ontology (GO) terms associated. During the blast step, we realized that some proteins had human or bacterial hits, 355 and 103 respectively. These sequences were removed from downstream analysis. Finally, the filtered assembly was composed by 43210 transcripts grouped into 32251 genes (Table 1). The mean GC content was 46.10%. The average and the median contig length were 1078.34 bp and 803 bp, respectively. The N50 was 1561 bp. The final dataset was then translated into proteins (minimum length 50 aa) obtaining a total of 31613 protein sequences. Among these, 14162 (44.79%) were complete protein sequences (with a methionine and a stop codon), 2782 (8.8%) started with a methionine but lacked a stop codon, 7957 (22%) had only a stop codon, 6712 (25.17%) did not start with a methionine and did not have a stop codon.

**Table 1:** *Cylindrotheca closterium* transcriptome assembly statistics.

Number of genes	32251
Number of transcripts	43210
Percent GC content	46.10
Contig N50	1561
Median contig length	803
Average contig length	1078.34
Number of proteins	31613
Number of complete proteins	14162
Number of partial proteins	17451

### Differential expression analysis

Differential expression analysis identified 1818 genes with significant expression variations in Si-starved condition compared to control. Among them, 845 were up-regulated (among these, 191 had an NCBI NR assignment), while 973 were down-regulated (among these, 361 had an NCBI NR assignment). The full list of DEGs, log fold change (logFC), false discovery rate (FDR) and their NCBI NR assignment are reported in the Supplementary Table 1.

Among the Differentially Expressed Genes (DEGs), Si-starvation induced an up-regulation on various unknown proteins, tkl dicty4 protein kinase (FDR < 1,35E-004), rna pseudouridine (FDR < 8,82E-003) and a transketolase (FDR < 0). Conversely, Si-starvation induced a strong down-regulation of vacuolar iron family transporter (FDR < 0), sodium bile acid cotransporter 7 isoform x1 (FDR < 6,48E-005) and two ABC transporters (FDR < 6,97E-009 and 1,01E-003, respectively).

Up-regulated transcripts were mainly involved in pathways related to bicarbonate transporters (R-HSA-425381), integrin cell surface interactions (R-HSA-216083) and unwinding of DNA (R-HSA-176974). On the contrary, transcripts involved in pathways related to Na+/Pi cotransporters (R-HSA-427589), transport of bile salts, organic acids, metal ions and amine compounds (R-HSA-425366), ABC transporters in lipid homeostasis (R-HSA-1369062), biotin transport (R-HSA-196780) and metabolism of folate and pterines (R-HSA-196757) were down-regulated when cultured in Si- starvation. In order to validate differential expression analysis between the control and Si-starvation conditions, 13 transcripts were tested using reverse transcription-quantitative PCR (RT-qPCR; gene names and primers are reported in Supplementary Tables 2 and 3). A significant positive correlation was established between RNAseq and RT-qPCR analyses (R = 0.8767, p value < 0.00001), supporting the results obtained by RNA-Seq.

### Identification of transcripts related to anti-inflammatory compound metabolism

We identified 5 transcripts coding for enzymes involved in the synthesis of compounds with possible anti-inflammatory activity: Phosphatidylinositol-3-Phosphatase (PtdIns(3)), Phosphatidylinositol 3-kinase catalytic subunit type 3 (PIK3C3), Phosphatidylinositol N-acetylglucosaminyltransferase subunit A (PIGA), Monogalactosyldiacylglycerol synthase (MGD), and Violaxanthin De-Epoxidase (VDE). These were selected as genes of interest (GOI) for this study.

Several inositol derivatives, such as phosphatidylinositol, are known to have anti-inflammatory activity^26,30,31^ and have been also quantified in several microalgae to be proposed as supplement in diet^32^. PtdIns(3), PIK3C3 and PIGA are the main enzymes involved in the inositol phosphate metabolism (KEGG pathway ec00562), and were selected as GOI for RT-qPCR analyses in this study. In particular, PtdIns(3) converts 1-phosphatidyl-1D-myo-inositol 3-phosphate in 1-phosphatidyl-1D-myo-inositol, while PIK3C3 and PIGA convert a 1, 2-diacyl-sn-glycero-3-phospho-(1D-myo-inositol) in a 1, 2-diacyl-sn-glycero-3-phospho-(1D-myo-inositol 3-phosphate) and a 6-(N-acetyl-α-D-glucosaminyl)-1-phosphatidyl-1D-myo-inositol, respectively (https://www.uniprot.org/uniprot/Q6PF93, https://www.uniprot.org/uniprot/P37287). Phosphatidylinositol 3-kinase is known to regulate key events in inflammatory responses, and there are also studies on genetically-modified mice with altered phosphatidylinositol 3-kinase signaling in order to understand its role in chronic inflammation mouse models^33^. Another selected transcript was MGD, essential for the synthesis of monogalactosyldiacylglycerols (MGDG)^34^. Several microalgae are known to be rich in MGDG containing a high proportion of polyunsaturated fatty acids (PUFAs) with potential nutraceutical and pharmaceutical applications^35^. MGDG are known to have anti-inflammatory activity reducing the release of inflammatory mediators (i.e. interleukins) and inhibiting the generation of superoxide anion^34^.

Finally, the transcript coding VDE was selected as well. VDE converts violaxanthin to zeaxanthin and both of them, as well as other carotenoids, are known to possess anti-inflammatory activities^36,37^ and have been widely studied in microalgae for their potential health benefits^15^. Primers were designed in order to amplify the five selected transcripts, their expression levels were analyzed in both control and Si-starvation conditions by RT-qPCR and *in silico* prediction of the three-dimensional structures of their corresponding proteins was performed.

### Reference gene assessment and GOI expression level analyses

**Figure 1:**
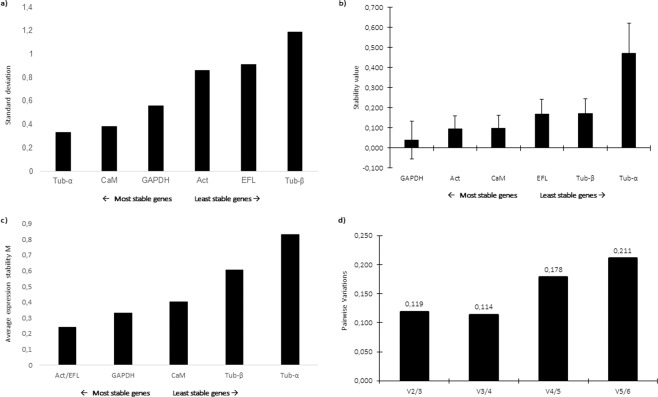
Reference gene assessment for *Cylindrotheca closterium*. Ranking of the best reference genes obtained with (**a**) BestKeeper (lowest standard deviation), (**b**) NormFinder (lowest expression stability value) and (**c**) geNorm (lowest average expression stability M) softwares. (**d**) According to geNorm algorithm, the inclusion of additional reference genes was not required for values below the cut-off of 0.15. The selected genes were: Actin (Act), Glyceraldehyde 3-Phosphate Dehydrogenase (GAPDH), Translation elongation factor-like protein (EFL), Calmodulin (CaM), α-tubulin (Tub-α) and β-tubulin (Tub-β).

In order to study GOIs expression levels, a panel of putative reference genes (RGs) was screened. Selected genes were included in those already used as RGs for other microalgae^38–42^. Results on the assessment of the best RGs obtained by three different software are reported in Fig. 1. According to the results obtained by BestKeeper, the lowest standard deviation (SD) was obtained for α-tubulin (Tub-α), followed by calmodulin (CaM) and glyceraldehyde 3-phosphate dehydrogenase (GAPDH) (Fig. 1a), which indicated that the most stable RG was Tub-α. According to NormFinder, the lowest stability values were calculated for GAPDH, Act and CaM; therefore, they were suggested as best candidate RGs (Fig. 1b). According to the statistical approach of geNorm, the two most stable genes (i.e., with the lowest expression stability, M) were actin (Act) and translation elongation factor-like protein (EFL) (Fig. 1c). Pairwise variation was calculated for assessment of the effect of adding another RG to those already analysed. The obtained results indicated that the addition of other RGs was not required since the value for V2/3 is below the cutoff of 0.15 (Fig. 1d). Considering the best RGs assigned by each software, Tub-α, GAPDH, Act and EFL were selected as RGs for RT-qPCR analyses. The best predicted RGs were different compared to other diatoms (such as *Skeletonema marinoi, Pseudo-nitzschia multistriata* and *P. arenysensis*^39,40,42^ since their stability is related to the studied species, growth phases and studied conditions (e.g. nutrient starvation/depletion and CO_2_ exposure).

**Figure 2:**
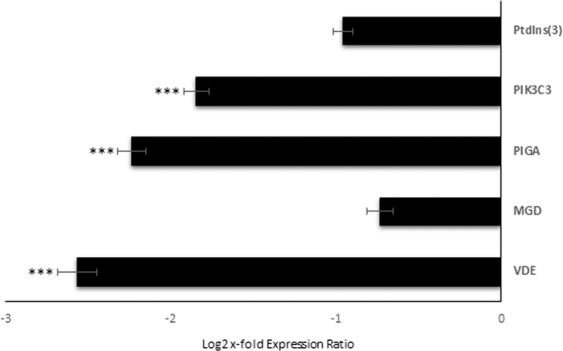
Expression levels of selected genes of interest in *C. closterium* cells cultured in silica starvation compared to the control condition. Data are represented as log2 x-fold expression ratio ± SD (n = 3). *C. closterium* cultured in complete medium was used as control (represented in the figure by x-axis; *** for p < 0.001). Gene abbreviations were Phosphatidylinositol-3-Phosphatase (PtdIns(3)), Phosphatidylinositol 3-kinase catalytic subunit type 3 (PIK3C3), Phosphatidylinositol N-acetylglucosaminyltransferase subunit A (PIGA), Monogalactosyldiacylglycerol synthase (MGD), and Violaxanthin De-Epoxidase (VDE).

Relative expression levels of the selected GOIs were analyzed in order to investigate the effect of Si-starvation on their expression. Figure 2 displays GOI expression levels in *C. closterium* cultured in Si-starvation compared to the control condition (x-axis). According to REST analysis, among the GOI, Gal-9, PIGA, PK3C3 and VDE showed significant down-regulation under Si-starvation (p < 0.001 for all), suggesting that there is higher probability to find their corresponding proteins and products in control conditions. For the other transcripts, changes were not significant. Even if whole transcriptome expression analyses in response to Si-starvation were already done for *Phaeodactylum tricornutum*^43^ and *Thalassiosira pseudonana*^44,45^, our GOIs were not previously studied. Hook *et al*.^46^ also sequenced the transcriptome of a *C. closterium* clone using 454 pyrosequencing and investigated its ecotoxicogenomic response by exposing the microalgae to various coastal contaminants (e.g. a photosystem II inhibiting herbicide, ammonia, copper and crude oil). However, due to differences in the aims of the studies and in the studied genes, a direct comparison with the present work was not possible.

### Domain identification and structure prediction of proteins encoded by selected GOI

With the aim of supporting the quality assessment of the selected GOIs, we performed an InterProScan analysis on the entire protein collection of *C. closterium*, obtaining an exhaustive functional characterization for each protein sequence. In Table 2, functional annotations from PFAM (protein families) and InterPro (protein domains) databases of five selected GOIs involved in anti-inflammatory compound metabolism are shown. This is the first study to investigate and identify transcripts involved in the synthesis of inflammatory mediators in diatoms and *in silico* predicting their three-dimensional structures.

**Table 2:** PFAM and INTERPRO annotation for each GOI.

SEQ ID	GOI	PFAM ANNOTATION	INTERPRO ANNOTATION
TR1438|c1_g1_i2	PtdIns(3)	SacI homology domain	SAC domain
TR3730|c0_g1_i2	PIK3C3	Phosphatidylinositol 3- and 4-kinase; Phosphoinositide 3-kinase family, accessory domain (PIK domain)	Phosphatidylinositol 3-/4-kinase, catalytic domain; Phosphoinositide 3-kinase, accessory (PIK) domain; Phosphatidylinositol 3-kinase, Vps34 type; Armadillo-type fold
TR15831|c0_g1_i2	PIGA	Glycosyl transferases group 1; PIGA (GPI anchor biosynthesis)	Glycosyl transferase, family 1; PIGA, GPI anchor biosynthesis
TR19402|c0_g2_i1	MGD	Monogalactosyldiacylglycerol (MGDG) synthase; Glycosyltransferase family 28 C-terminal domain	Diacylglycerol glucosyltransferase, N-terminal; Glycosyl transferase, family 28, C-terminal
TR16349|c0_g1_i1	VDE	VDE lipocalin domain	VDE lipocalin domain; Calycin

**Figure 3:**
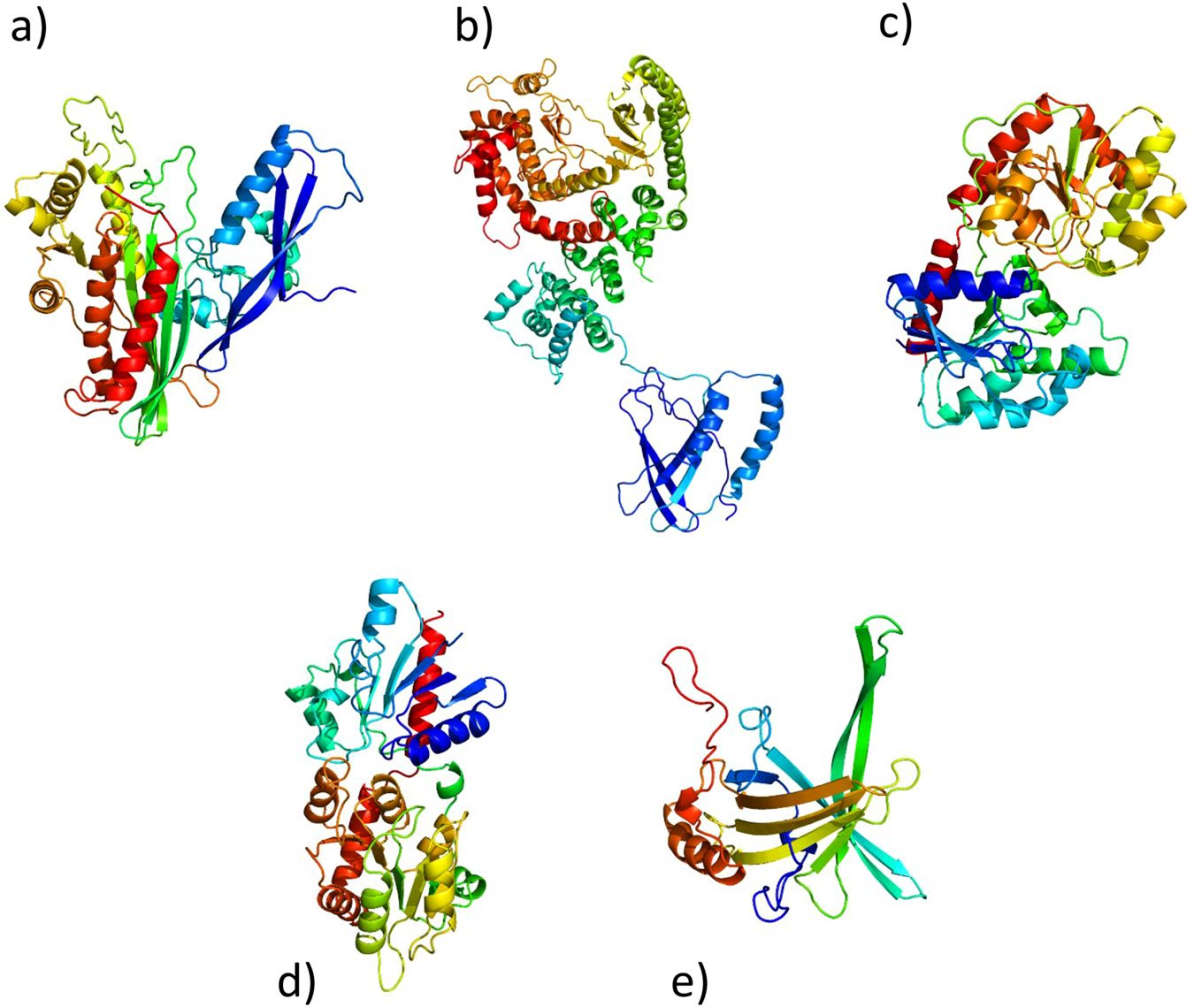
*In silico* protein structure predictions of five proteins encoded by the selected genes of interest. Protein structure predictions, colored by rainbow from N to C terminus, for Gal-9 (**a**), PtdIns(3) (**b**), PIK3C3 (**c**), PIGA (**d**), MGD (**e**) and VDE (**f**). Three-dimensional structures are indicated as follows: α-helices are represented by helices, β-strands are represented by arrows, coils are represented by simple lines.

In order to further investigate the selected GOIs at protein structure levels, we *in silico* predicted their three-dimensional structure by fold recognition approach (Kelley *et al*. 2015 https://www.ncbi.nlm.nih.gov/pubmed/25950237) (Fig. 3). The modeled structures showed PHYRE2 confidence scores that range between 99, 5% and 100%.

### Anti-inflammatory testing

*C. closterium* anti-inflammatory activity was screened by monitoring the release of tumor necrosis factor α (TNFα) in human THP-1 cells. Microalgae cultured in Si-starvation condition did not show anti-inflammatory activity (8% TNFα-inhibition, p > 0.05). Similarly, previous studies have shown that *C. closterium* cultured in nitrogen- and phosphate-starvation did not show anti-inflammatory activity, while the same species cultured in control condition (complete medium) induced more than 80% TNFα-inhibition^10^. These data suggest that in stressful conditions, such as nutrient starvation, *C. closterium* should not produce anti-inflammatory molecule/s or produce very low amounts. This information will direct future chemical analyses in *C. closterium* cultured in control conditions and guide marine natural product discovery increasing diatom applications in the blue biotechnology sector.

## Materials and Methods

### Cell culturing and harvesting

The diatom *Cylindrotheca closterium* was cultured in Guillard’s F/2 medium^47^. Experimental culturing was performed in triplicate for both control (CTRL) or Si-starvation conditions. In particular, microalgae cultured in control condition were cultivated in complete F/2 medium^47^, while for Si-starvation experiments, 36 μM Si(OH)_4_ was used. Culturing was carried out in 2-litre polycarbonate bottles, constant bubbling with air filtered through 0.2 μm membrane filters in a climate chamber at 19 °C on a 12:12 hours light:dark cycle and at 100 μmol photons m^−2^ s^−1^. Initial cell concentrations were approximately 5000 cells/mL for each replicate, and culture growth was daily monitored from samples fixed with one drop of Lugol (final concentration of about 2%) and counted in a Bürker counting chamber under an Axioskop 2 microscope (20×) (Carl Zeiss GmbH). Aliquots of 50 ml were sampled when the stationary phase was reached (Day 7), and centrifuged for 15 minutes at 4 °C at 1900 g (Eppendorf, 5810R). For RNA extraction, microalgal pellets were resuspended in 500 μL of TRIZOL© (Invitrogen, Carlsbad, CA), incubated for 2–3 min at 60 °C until completely dissolved, frozen in liquid nitrogen and kept at −80 °C till RNA extraction procedure (as in^40^). For chemical extraction and anti-inflammatory activity, pellets were frozen in liquid nitrogen and kept at −80 °C till further processing.

### RNA extraction

RNA extraction was performed following TRIZOL® manufacturer’s instructions (as in^40^). Afterwards, each sample was treated with DNase I (Invitrogen) using the instruction’s manual in order to remove hypothetically contaminating DNA. The assessment of RNA quantity was carried out by using Nano-Drop (ND-1000 UV–Vis spectrophotometer; NanoDrop Technologies), monitoring the absorbance at 260 nm and the 260/280 nm and 260/230 nm ratios. RNA samples were considered good when both ratios were approximately 2. RNA quality was also assessed on 1% agarose gel, showing intact 18S and 28S ribosomal bands. Total RNA quality was evaluated by measuring the RNA Integrity Number (RIN) with Agilent 2100 Bioanalyzer (Agilent Technologies, Inc.). High quality (RIN > 8) RNA was used for both RNAseq and RT-qPCR.

### RNA sequencing

Next generation sequencing experiments, including samples quality control, were performed by Genomix4life S.R.L. (Baronissi, Salerno, Italy). Indexed libraries were prepared from 2 ug/ea purified RNA with TruSeq Stranded mRNA Sample Prep Kit (Illumina) according to the manufacturer’s instructions. Libraries were quantified using the Agilent 2100 Bioanalyzer (Agilent Technologies) and pooled in a way that each index-tagged sample was present in equimolar amounts, with final concentration of the pooled samples of 2 nM. The pooled samples were subject to cluster generation and sequencing using an Illumina HiSeq. 2500 System (Illumina) in a 2 × 100 paired-end format at a final concentration of 8 pmol. The raw sequence files generated (.fastq files) underwent quality control analysis using FastQC (http://www.bioinformatics.babraham.ac.uk/projects/fastqc/).

### Transcriptome assembly, annotation, expression quantification and differential expression analysis

Illumina paired-end 100 bp reads were processed to produce the transcriptome assembly. Reads are freely available under the series entry PRJNA577416 in the NCBI BioProject database. Raw reads were trimmed and clipped with BBDuk (https://jgi.doe.gov/data-and-tools/bbtools/) setting a minimum Phred-like quality of 25 and a minimum length of 35 nucleotides. The quality of the reads before and after trimming was checked with the software FASTQC (http://www.bioinformatics.babraham.ac.uk/projects/fastqc/). High quality reads were then normalized with Trinity^48^ using the options:–SS_lib_type RF–pairs_together–max_cov 50. De novo transcriptome assembly was then performed with Trinity using the options:–SS_lib_type RF–no_normalize_reads–min_kmer_cov 1–KMER_SIZE 32. Transcriptome redundancy was removed with CD-HIT-EST^49^ using the following options: -r 0 -g 1. A filter for contaminants was applied by performing a BLAST search of the transcripts against the NCBI nr database, discarding all the sequences having a significant hit (evalue <= 0.0001) against bacteria or metazoa. The completeness of the assembly was checked against the Core Eukaryotic Genes database (http://korflab.ucdavis.edu/Datasets/genome_completeness/ and http://korflab.ucdavis.edu/datasets/cegma/). *In silico* translation was performed with TransDecoder^50^ whereas Functional Annotation was performed with Blast2GO software^51^. Transcript expression quantification was performed using Express (v 1.5.1)^52^ after mapping the reads against the assembly with STAR^53^. Posterior counts were used as input to perform transcript differential expression analysis with EBSeq.^54^, and differentially expressed transcripts with |LogFC| > 2 and FDR ≤ 0.01 were considered significant.

### Selection and characterization of Putative Reference Genes (RGs) and Genes of Interest (GOIs) and Primer Design

The selection of putative reference genes (RGs) was carried out between a series of genes previously used as reference for other microalgae^39,41,42^. The selected genes were: Actin (Act), Glyceraldehyde 3-Phosphate Dehydrogenase (GAPDH), Translation elongation factor-like protein (EFL), Calmodulin (CaM), α-tubulin (Tub-α) and β-tubulin (Tub-β). Their primary function in the cell is reported in Supplementary Table 2. Regarding genes of interest (GOIs), transcripts coding enzymes involved in the metabolism of anti-inflammatory compounds were selected. Transdecoder software (https://github.com/TransDecoder/TransDecoder/wiki) was used to translate into protein sequences the entire *C. closterium* collection of transcripts. InterProScan (version 5.33) software^55^ was used to scan the entire collection of protein sequences of *C. closterium* (activating the “iprlookup” parameter) against the InterPro database^56^, a reference collection for protein domains and functional information. We selected the Reactome database^57^ to associate metabolic pathways information to DEGs. We also used Kyoto Encyclopedia of Genes and Genomes (KEGG) pathway database during the selection of the genes of interests to look for pathways of interest, such as the Inositol Phosphate Metabolism Pathway since inositol derivatives are known as anti-inflammatory agents^31^ (https://www.genome.jp/kegg/pathway.html). The chosen GOIs were: Phosphatidylinositol-3-Phosphatase (PtdIns(3)), Phosphatidylinositol 3-kinase catalytic subunit type 3 (PIK3C3), Phosphatidylinositol N-acetylglucosaminyltransferase subunit A (PIGA), Monogalactosyldiacylglycerol synthase (MGD) and Violaxanthin De-Epoxidase (VDE) (as reported in Supplementary Table 2). Protein structures of the selected GOIs were predicted through the Phyre2 web server^58^ (http://www.sbg.bio.ic.ac.uk/phyre2/html/page.cgi?id=index). In order to perform RNA-seq validation by RT-qPCR, other GOIs, chosen between the most up- or down-regulated differentially expressed genes (DEGs) with functional annotation, were selected: Adenylate and Guanylate cyclase catalytic domain-containing protein (AG), 3′5′-cyclic nucleotide phosphodiesterase (PDEase), Transposon Protein (TE), Nitrate Transporter (NRT), Vacuolar Iron Family Transporter (VIT), rRNA pseudouridylate synthase (Rsu) and ATP-Binding Cassette Protein Transporter (ABC). Their primary function in the cell is reported in Supplementary Table 2. Primers were designed using the software Primer3 version 4.1.0 (http://primer3.ut.ee/). In order to evaluate the cross-comparison of assays and assure equal PCR efficiencies, the size of amplicons was kept in the range of 150 to 250 base pairs, GC content at 50%, the length of primers between 19 and 21 nucleotides, and melting temperature from 59 °C to 61 °C. Primers were synthesized by Sigma-Aldrich. PCR conditions were optimized on a GeneAmp PCR System 9700 (Perkin Elmer) as in Lauritano *et al*.^59^. Supplementary Table 3 illustrates primer sequences for all the selected genes as well as their amplicon sizes, oligo efficiencies (E) and correlation factors (R2).

### cDNA synthesis and Reverse Transcription-Quantitative Polymerase Chain Reaction (RT-qPCR)

RNA samples (1 μg/each) were retrotranscribed into complementary DNA (cDNA) by using the iScriptTM cDNA Synthesis Kit (BIORAD, Hercules, CA) following the manufacturer’s instructions, in the GeneAmp PCR System 9700 (Perkin Elmer). The obtained cDNA (1:10 dilution) was used as template for RT-qPCR experiments performed in a Viia7 real-time PCR system (Applied Biosystems). PCR volume of each sample was 10 μl with 5 μl of Fast Start SYBR Green Master Mix (Roche), 0.7 pmol/ μl for each oligo and 1 μl of cDNA template. Experiments were performed as in Lauritano *et al*.^38^. Best reference genes were identified using three different algorithms, i.e. BestKeeper^60^, geNorm^61^ and NormFinder^62^. To study expression levels for each gene of interest relative to the most stable RGs, we used the REST tool (Relative Expression Software Tool)^63^. Statistical analysis was performed using GraphPad Prim Statistic Software, V4.00 (GraphPad Software; http://www.graphpad.com/quickcalcs/).

### Chemical Extraction, Pre-Fractionation and Anti-inflammatory testing

Chemical extraction was performed as in Lauritano *et al*.^10^ by using Amberlite XAD16N resin (20–60 mesh, Sigma-Aldrich). The final extracts were freeze-dried and stored at −20 °C until screening. Before performing the assays, extracts were first diluted at 1 mg/mL with MilliQ water and 2.5% DMSO. A triplicate of control plus the same concentration of DMSO used in test wells was used in all assays. The anti-inflammatory assay was performed as in Lind *et al*. (2013). Briefly, ~10^6^ human monocyte THP-1 cells/mL (ATCC^(R) TIB-202TM^) supplemented with 50 ng/mL phorbol 12-myristate 13-acetate (PMA, SigmaAldrich) were seeded in 96 well plates and incubated at 37 °C, 5% CO_2_ for 48 h in RPMI-1640 medium (Biochrom;10%FBS). After 72 h, 80 μL fresh RPMI medium and 10 μL/well (tested concentration 100 μg/mL) of test extract were added. The test was performed in triplicate. After incubation for 1 h, all samples were incubated with 1 ng/mL lipopolysaccharide (LPS; final concentration) for another 6 h at 37 °C. Enzyme-linked immunosorbent Assay (ELISA) was used to test TNFα secretion as in Lauritano *et al*.^10^.

## Supplementary information

Supplementary information.

## Data Availability

Data are available and sequences are deposited in the NCBI Sequence Read Archive (SRA) database.
