# Supplementary material for: De novo transcriptome of the diatom Cylindrotheca closterium identifies genes involved in the metabolism of anti-inflammatory compounds
Source: Sci Rep. 2020 Mar 5;10:4138. doi: 10.1038/s41598-020-61007-0 (PMC7058042; doi:10.1038/s41598-020-61007-0)
Supplement: Supplementary file 1 — Supplementary information. [file 41598_2020_61007_MOESM1_ESM.docx]

Supplementary Information of

***De novo* transcriptome of the diatom *Cylindrotheca closterium* identifies genes involved in the metabolism of anti-inflammatory compounds**

**Ali M. Elagoz ^1,2^, Luca Ambrosino ^3^, Chiara Lauritano ^1,^***

^1^ Department of Integrative Marine Ecology, Stazione Zoologica Anton Dohrn, Villa Comunale, 80121, Napoli, Italy;

^2^ Ghent University, Marine Biology Research Group, Krijgslaan 281, B-9000 Gent, Belgium;

^3^ Research Infrastructure for Marine Biological Resources Department, Stazione Zoologica Anton Dohrn, Villa Comunale, CAP80121 (NA), Italy;

***** Correspondence: [chiara.lauritano@szn.it](mailto:chiara.lauritano@szn.it); Tel.: +39 081 5833221 (C.L.)

**Supplementary Table 1.** Full list of differentially expressed genes (DEGs), false discovery rate (FDR), log fold change (logFC) and their NCBI NR assignment

| **Transcript ID** | **FDR** | **LogFC** | **NCBI NR assignment** |
| --- | --- | --- | --- |
| TR8290\|c0_g2_i2 | 0.00E+000 | 20.08 |  |
| TR10484\|c1_g1_i7 | 0.00E+000 | 19.72 |  |
| TR8290\|c0_g2_i1 | 0.00E+000 | 19.64 |  |
| TR6658\|c3_g1_i2 | 0.00E+000 | 19.33 |  |
| TR3705\|c0_g3_i7 | 1.82E-009 | 18.78 |  |
| TR2334\|c0_g3_i7 | 1.35E-004 | 18.48 | tkl dicty4 protein kinase |
| TR9478\|c0_g1_i1 | 9.57E-007 | 18.13 |  |
| TR20428\|c0_g1_i2 | 1.57E-003 | 18.02 |  |
| TR18573\|c0_g2_i9 | 1.26E-003 | 17.96 | protein |
| TR16263\|c0_g1_i7 | 1.70E-004 | 17.87 |  |
| TR11328\|c1_g1_i5 | 8.33E-003 | 17.52 |  |
| TR8594\|c0_g1_i1 | 6.72E-003 | 17.24 |  |
| TR17682\|c2_g1_i2 | 4.34E-006 | 17.21 |  |
| TR5356\|c0_g1_i1 | 2.66E-003 | 17.19 |  |
| TR14382\|c0_g1_i1 | 7.74E-003 | 16.86 |  |
| TR16039\|c0_g1_i2 | 4.11E-003 | 16.68 |  |
| TR6451\|c0_g1_i2 | 9.85E-006 | 16.55 |  |
| TR16319\|c0_g1_i7 | 0.00E+000 | 16.17 |  |
| TR21125\|c1_g1_i8 | 2.46E-004 | 15.01 | protein |
| TR3737\|c13_g1_i5 | 8.70E-004 | 14.98 |  |
| TR16039\|c0_g1_i3 | 0.00E+000 | 13.87 |  |
| TR19917\|c1_g1_i1 | 1.65E-006 | 13.76 |  |
| TR2294\|c1_g2_i4 | 2.92E-004 | 13.23 |  |
| TR2249\|c4_g2_i6 | 0.00E+000 | 12.89 |  |
| TR23223\|c4_g1_i8 | 6.55E-015 | 11.92 |  |
| TR3700\|c0_g1_i2 | 5.93E-003 | 11.11 |  |
| TR3130\|c1_g1_i2 | 9.63E-009 | 10.54 |  |
| TR5543\|c3_g1_i4 | 2.82E-008 | 10.45 |  |
| TR8327\|c0_g1_i20 | 1.66E-003 | 9.41 |  |
| TR11372\|c0_g2_i1 | 2.73E-004 | 9.35 |  |
| TR18664\|c0_g1_i2 | 3.47E-007 | 9.24 |  |
| TR5176\|c0_g2_i1 | 7.42E-004 | 9.22 |  |
| TR16386\|c0_g1_i1 | 8.82E-003 | 9.20 | rna pseudouridine |
| TR23279\|c4_g1_i9 | 0.00E+000 | 8.05 | protein |
| TR6213\|c0_g1_i2 | 8.78E-003 | 7.92 |  |
| TR19113\|c0_g2_i1 | 1.49E-003 | 7.71 |  |
| TR1474\|c0_g1_i7 | 0.00E+000 | 7.71 | transketolase |
| TR25164\|c0_g1_i1 | 7.84E-003 | 7.67 |  |
| TR3707\|c0_g1_i6 | 7.48E-007 | 7.19 |  |
| TR1438\|c1_g1_i2 | 0.00E+000 | 7.04 | phosphatidylinositol phosphatase |
| TR22953\|c0_g1_i1 | 1.77E-012 | 6.65 |  |
| TR8760\|c0_g1_i2 | 8.19E-003 | 6.54 | frustulin-like protein |
| TR16020\|c0_g2_i3 | 8.20E-008 | 6.51 |  |
| TR10494\|c1_g1_i1 | 3.20E-003 | 6.44 |  |
| TR5575\|c3_g1_i3 | 5.42E-013 | 6.43 |  |
| TR2339\|c0_g1_i5 | 1.55E-004 | 6.30 |  |
| TR7184\|c0_g1_i1 | 1.11E-003 | 6.25 |  |
| TR8359\|c0_g1_i1 | 0.00E+000 | 6.11 |  |
| TR23278\|c2_g3_i3 | 1.85E-007 | 6.04 |  |
| TR25383\|c0_g1_i2 | 1.37E-003 | 5.85 | trna (adenine -n )-methyltransferase catalytic subunit trmt61a |
| TR14781\|c2_g1_i3 | 4.70E-006 | 5.74 | protein |
| TR24424\|c0_g1_i4 | 1.99E-005 | 5.70 |  |
| TR3738\|c3_g4_i1 | 0.00E+000 | 5.67 |  |
| TR2249\|c4_g2_i3 | 1.56E-003 | 5.64 |  |
| TR2164\|c0_g1_i1 | 7.39E-004 | 5.63 |  |
| TR20368\|c0_g2_i6 | 0.00E+000 | 5.60 |  |
| TR84\|c0_g1_i3 | 1.65E-006 | 5.57 |  |
| TR16276\|c0_g1_i1 | 3.57E-004 | 5.53 |  |
| TR21833\|c0_g1_i2 | 7.54E-003 | 5.53 |  |
| TR16263\|c0_g1_i2 | 1.13E-004 | 5.47 |  |
| TR16868\|c0_g1_i2 | 1.02E-012 | 5.47 |  |
| TR25383\|c0_g1_i3 | 5.34E-004 | 5.44 | trna (adenine -n )-methyltransferase catalytic subunit trmt61a |
| TR23223\|c3_g1_i3 | 0.00E+000 | 5.39 | tkl dicty4 protein kinase |
| TR11777\|c0_g1_i2 | 0.00E+000 | 5.34 |  |
| TR23223\|c3_g1_i1 | 0.00E+000 | 5.30 | tkl dicty4 protein kinase |
| TR1045\|c0_g1_i1 | 0.00E+000 | 5.28 |  |
| TR25492\|c0_g1_i2 | 1.28E-003 | 5.27 |  |
| TR10484\|c1_g1_i10 | 6.99E-003 | 5.16 | myosin-1 |
| TR7982\|c0_g1_i4 | 4.82E-005 | 5.11 |  |
| TR5206\|c1_g1_i2 | 3.63E-004 | 5.09 |  |
| TR21067\|c1_g2_i8 | 2.97E-003 | 5.05 | atp-dependent rna |
| TR23289\|c6_g1_i4 | 2.11E-003 | 5.03 |  |
| TR1383\|c0_g1_i1 | 5.25E-004 | 4.99 | protein |
| TR19941\|c0_g2_i1 | 0.00E+000 | 4.97 |  |
| TR5536\|c0_g2_i1 | 1.00E-003 | 4.96 |  |
| TR5572\|c1_g1_i3 | 1.95E-003 | 4.95 |  |
| TR13459\|c0_g1_i2 | 4.55E-003 | 4.91 |  |
| TR11276\|c9_g2_i5 | 1.52E-004 | 4.89 |  |
| TR22145\|c0_g1_i1 | 5.86E-011 | 4.87 |  |
| TR1124\|c0_g1_i2 | 4.16E-004 | 4.86 |  |
| TR3721\|c1_g2_i1 | 3.22E-009 | 4.82 |  |
| TR11324\|c1_g2_i4 | 6.02E-004 | 4.79 |  |
| TR7339\|c0_g1_i2 | 2.77E-005 | 4.78 |  |
| TR5620\|c0_g1_i1 | 0.00E+000 | 4.76 |  |
| TR18313\|c1_g1_i1 | 0.00E+000 | 4.73 |  |
| TR23485\|c0_g1_i1 | 1.89E-004 | 4.73 |  |
| TR6794\|c0_g1_i1 | 8.70E-004 | 4.73 |  |
| TR20608\|c0_g2_i2 | 0.00E+000 | 4.66 |  |
| TR16120\|c0_g1_i2 | 0.00E+000 | 4.61 | tkl dicty4 drk protein kinase |
| TR11777\|c0_g1_i3 | 0.00E+000 | 4.59 |  |
| TR7184\|c0_g1_i7 | 1.96E-004 | 4.57 |  |
| TR26008\|c0_g2_i1 | 0.00E+000 | 4.57 |  |
| TR11767\|c0_g1_i1 | 0.00E+000 | 4.55 | protein |
| TR7477\|c0_g1_i7 | 4.57E-003 | 4.49 | predicted protein |
| TR16295\|c0_g2_i4 | 3.58E-004 | 4.49 |  |
| TR20694\|c0_g1_i1 | 8.12E-005 | 4.48 |  |
| TR2269\|c4_g2_i8 | 5.96E-011 | 4.40 |  |
| TR2269\|c4_g2_i1 | 4.15E-011 | 4.40 |  |
| TR9862\|c0_g1_i1 | 8.40E-004 | 4.40 |  |
| TR13161\|c0_g1_i4 | 1.50E-003 | 4.36 |  |
| TR10402\|c0_g1_i2 | 1.66E-008 | 4.33 | short chain dehydrogenase reductase family protein |
| TR21074\|c1_g4_i6 | 6.23E-006 | 4.32 | protein |
| TR25848\|c0_g1_i1 | 0.00E+000 | 4.29 |  |
| TR3728\|c1_g2_i4 | 4.67E-004 | 4.28 |  |
| TR9771\|c0_g1_i1 | 6.72E-010 | 4.27 |  |
| TR12502\|c1_g1_i2 | 6.88E-009 | 4.26 |  |
| TR3721\|c2_g1_i7 | 0.00E+000 | 4.26 |  |
| TR1844\|c0_g1_i1 | 2.32E-013 | 4.25 |  |
| TR9333\|c0_g1_i1 | 1.19E-008 | 4.23 |  |
| TR25899\|c4_g2_i1 | 1.49E-005 | 4.23 |  |
| TR10230\|c0_g1_i2 | 4.61E-009 | 4.22 |  |
| TR7976\|c0_g1_i1 | 5.79E-003 | 4.21 |  |
| TR5643\|c0_g2_i1 | 2.21E-004 | 4.21 |  |
| TR3444\|c0_g2_i1 | 6.83E-005 | 4.18 |  |
| TR23297\|c1_g1_i1 | 1.10E-004 | 4.16 |  |
| TR20125\|c7_g3_i1 | 7.15E-003 | 4.14 |  |
| TR14998\|c0_g1_i1 | 7.06E-005 | 4.14 |  |
| TR25899\|c2_g1_i1 | 1.53E-012 | 4.14 |  |
| TR16120\|c0_g1_i1 | 0.00E+000 | 4.14 | tkl dicty4 drk protein kinase |
| TR18900\|c0_g1_i1 | 8.17E-003 | 4.11 |  |
| TR5568\|c0_g1_i3 | 0.00E+000 | 4.10 |  |
| TR20221\|c0_g1_i2 | 0.00E+000 | 4.10 |  |
| TR21061\|c7_g3_i9 | 0.00E+000 | 4.10 | tkl dicty4 protein kinase |
| TR17884\|c0_g1_i1 | 2.43E-003 | 4.06 |  |
| TR5172\|c0_g1_i2 | 6.72E-008 | 4.05 |  |
| TR10554\|c0_g1_i5 | 4.20E-003 | 4.05 |  |
| TR15976\|c1_g1_i1 | 0.00E+000 | 4.03 |  |
| TR616\|c0_g1_i1 | 7.33E-015 | 4.00 |  |
| TR3770\|c1_g1_i1 | 0.00E+000 | 3.98 |  |
| TR18824\|c0_g1_i1 | 6.48E-003 | 3.98 |  |
| TR7251\|c0_g1_i1 | 0.00E+000 | 3.98 |  |
| TR3091\|c0_g2_i5 | 7.60E-006 | 3.97 |  |
| TR3735\|c1_g3_i2 | 1.65E-004 | 3.95 | protein |
| TR11181\|c0_g1_i1 | 4.85E-007 | 3.95 |  |
| TR25238\|c1_g1_i1 | 0.00E+000 | 3.95 |  |
| TR25657\|c0_g1_i1 | 4.79E-006 | 3.93 | protein |
| TR16595\|c0_g1_i1 | 1.19E-003 | 3.93 |  |
| TR9415\|c0_g1_i1 | 1.00E-003 | 3.93 | surface antigen -like |
| TR21773\|c0_g1_i1 | 0.00E+000 | 3.92 |  |
| TR20238\|c0_g1_i2 | 0.00E+000 | 3.91 |  |
| TR7884\|c0_g2_i2 | 0.00E+000 | 3.91 |  |
| TR12035\|c0_g1_i1 | 1.63E-005 | 3.89 |  |
| TR285\|c0_g1_i1 | 0.00E+000 | 3.89 | hypothetical leucine rich repeat protein |
| TR24437\|c1_g1_i2 | 0.00E+000 | 3.87 | frustulin 5 |
| TR10554\|c1_g10_i9 | 4.10E-010 | 3.86 |  |
| TR21074\|c1_g4_i9 | 1.36E-008 | 3.86 |  |
| TR10499\|c1_g1_i5 | 1.83E-006 | 3.85 | photolyase blue-light receptor partial |
| TR13598\|c0_g2_i1 | 8.88E-016 | 3.85 |  |
| TR19379\|c4_g1_i1 | 3.11E-003 | 3.84 | protein |
| TR20202\|c0_g1_i1 | 0.00E+000 | 3.83 |  |
| TR24252\|c0_g1_i1 | 0.00E+000 | 3.82 |  |
| TR9699\|c0_g1_i1 | 6.59E-010 | 3.81 | protein |
| TR18052\|c0_g1_i1 | 4.03E-004 | 3.81 |  |
| TR20246\|c0_g1_i2 | 0.00E+000 | 3.80 |  |
| TR20067\|c1_g1_i1 | 0.00E+000 | 3.80 | reverse transcriptase |
| TR25313\|c1_g1_i1 | 0.00E+000 | 3.79 |  |
| TR12049\|c0_g1_i2 | 1.46E-005 | 3.77 |  |
| TR3702\|c0_g1_i2 | 5.37E-003 | 3.74 |  |
| TR3994\|c0_g1_i1 | 2.20E-003 | 3.73 |  |
| TR11360\|c3_g2_i3 | 1.82E-008 | 3.72 |  |
| TR21486\|c0_g1_i1 | 8.35E-003 | 3.72 |  |
| TR334\|c0_g1_i1 | 1.71E-009 | 3.70 |  |
| TR9217\|c0_g2_i1 | 0.00E+000 | 3.69 |  |
| TR19807\|c0_g1_i1 | 2.87E-003 | 3.68 |  |
| TR2265\|c0_g1_i3 | 6.10E-008 | 3.67 | protein |
| TR25313\|c0_g1_i1 | 0.00E+000 | 3.67 |  |
| TR1698\|c0_g1_i1 | 9.73E-003 | 3.67 |  |
| TR7519\|c0_g7_i2 | 0.00E+000 | 3.66 |  |
| TR25543\|c0_g1_i1 | 6.69E-013 | 3.66 |  |
| TR4416\|c0_g1_i2 | 3.00E-004 | 3.64 |  |
| TR24638\|c0_g1_i5 | 3.25E-009 | 3.64 |  |
| TR23223\|c4_g1_i12 | 1.54E-008 | 3.63 |  |
| TR15167\|c0_g1_i3 | 6.59E-012 | 3.61 |  |
| TR19208\|c0_g1_i1 | 3.53E-005 | 3.61 |  |
| TR24424\|c0_g1_i2 | 4.18E-004 | 3.61 |  |
| TR10531\|c5_g3_i1 | 2.50E-003 | 3.60 |  |
| TR10209\|c0_g1_i1 | 3.11E-011 | 3.60 |  |
| TR13389\|c0_g2_i1 | 9.97E-006 | 3.60 |  |
| TR10511\|c6_g2_i6 | 4.77E-003 | 3.59 | protein |
| TR8983\|c0_g1_i1 | 0.00E+000 | 3.58 |  |
| TR24178\|c0_g1_i1 | 1.92E-009 | 3.58 |  |
| TR10431\|c1_g1_i1 | 1.68E-005 | 3.57 |  |
| TR4588\|c0_g1_i1 | 0.00E+000 | 3.57 | protein kinase |
| TR3711\|c0_g1_i5 | 0.00E+000 | 3.56 | aminotransferase class-iii |
| TR12962\|c0_g1_i1 | 0.00E+000 | 3.56 |  |
| TR12529\|c0_g1_i3 | 0.00E+000 | 3.55 | lpxtg-motif cell wall anchor domain protein |
| TR13161\|c0_g1_i3 | 0.00E+000 | 3.55 |  |
| TR14399\|c0_g1_i2 | 1.11E-005 | 3.51 |  |
| TR10509\|c3_g4_i1 | 0.00E+000 | 3.51 | frustulin-like protein |
| TR18425\|c0_g1_i1 | 0.00E+000 | 3.51 |  |
| TR14782\|c1_g1_i2 | 0.00E+000 | 3.49 |  |
| TR12502\|c1_g1_i4 | 1.46E-012 | 3.49 |  |
| TR25103\|c0_g1_i2 | 0.00E+000 | 3.49 |  |
| TR24373\|c0_g1_i2 | 8.36E-003 | 3.49 |  |
| TR16263\|c0_g1_i4 | 0.00E+000 | 3.48 |  |
| TR20888\|c0_g1_i1 | 0.00E+000 | 3.47 |  |
| TR3732\|c0_g2_i4 | 0.00E+000 | 3.46 |  |
| TR11357\|c2_g3_i2 | 5.66E-015 | 3.45 |  |
| TR2079\|c0_g1_i1 | 0.00E+000 | 3.45 |  |
| TR12909\|c0_g1_i1 | 5.25E-005 | 3.45 |  |
| TR7511\|c5_g1_i1 | 2.22E-007 | 3.44 |  |
| TR25127\|c0_g1_i1 | 1.53E-012 | 3.44 |  |
| TR2320\|c4_g5_i4 | 6.20E-005 | 3.43 |  |
| TR20113\|c5_g4_i5 | 0.00E+000 | 3.43 |  |
| TR1950\|c0_g1_i1 | 2.06E-010 | 3.43 |  |
| TR13422\|c0_g1_i1 | 2.22E-016 | 3.43 |  |
| TR7433\|c0_g1_i1 | 4.17E-007 | 3.43 |  |
| TR20392\|c0_g1_i1 | 0.00E+000 | 3.42 |  |
| TR3732\|c0_g2_i3 | 4.99E-007 | 3.41 |  |
| TR19380\|c0_g1_i2 | 0.00E+000 | 3.40 |  |
| TR19941\|c0_g1_i1 | 0.00E+000 | 3.40 |  |
| TR14036\|c0_g1_i1 | 0.00E+000 | 3.39 |  |
| TR11484\|c0_g2_i1 | 9.22E-005 | 3.39 |  |
| TR12049\|c0_g1_i1 | 0.00E+000 | 3.38 |  |
| TR5371\|c0_g1_i1 | 7.36E-014 | 3.38 |  |
| TR24988\|c0_g1_i3 | 4.44E-011 | 3.37 |  |
| TR8539\|c0_g1_i1 | 2.22E-016 | 3.37 | tkl dicty4 protein kinase |
| TR4391\|c0_g1_i1 | 0.00E+000 | 3.37 |  |
| TR15607\|c0_g1_i1 | 5.20E-010 | 3.36 | atp-dependent rna helicase |
| TR18416\|c1_g1_i1 | 0.00E+000 | 3.36 | protein |
| TR10122\|c0_g1_i1 | 0.00E+000 | 3.35 |  |
| TR17517\|c0_g1_i1 | 0.00E+000 | 3.35 | serine protease p27 |
| TR2313\|c0_g4_i2 | 8.87E-004 | 3.34 |  |
| TR3759\|c1_g1_i1 | 1.48E-004 | 3.34 | predicted protein |
| TR3124\|c1_g1_i6 | 0.00E+000 | 3.33 |  |
| TR6568\|c0_g1_i3 | 2.06E-006 | 3.33 |  |
| TR10850\|c2_g1_i11 | 8.83E-003 | 3.32 |  |
| TR11851\|c0_g1_i1 | 0.00E+000 | 3.32 |  |
| TR23421\|c0_g1_i1 | 1.37E-007 | 3.31 |  |
| TR15038\|c0_g1_i2 | 2.10E-004 | 3.31 |  |
| TR12535\|c0_g1_i1 | 2.00E-015 | 3.29 | transcription factor gamyb |
| TR25242\|c0_g1_i1 | 1.37E-008 | 3.28 |  |
| TR17067\|c0_g1_i1 | 1.83E-004 | 3.28 |  |
| TR5524\|c0_g1_i1 | 0.00E+000 | 3.28 |  |
| TR6321\|c0_g1_i1 | 0.00E+000 | 3.26 | adenosine deaminase |
| TR10878\|c0_g1_i3 | 0.00E+000 | 3.26 |  |
| TR9421\|c0_g1_i1 | 3.75E-008 | 3.25 |  |
| TR7312\|c1_g1_i5 | 1.60E-003 | 3.25 | lactosylceramide 4-alpha-galactosyltransferase |
| TR2244\|c0_g2_i9 | 0.00E+000 | 3.24 |  |
| TR24128\|c0_g1_i1 | 3.04E-007 | 3.23 | atpase afg2 protein |
| TR6451\|c0_g1_i1 | 0.00E+000 | 3.23 |  |
| TR8365\|c0_g1_i1 | 0.00E+000 | 3.23 |  |
| TR20255\|c0_g1_i1 | 0.00E+000 | 3.23 |  |
| TR2559\|c0_g1_i1 | 1.15E-006 | 3.22 |  |
| TR7519\|c0_g7_i1 | 0.00E+000 | 3.21 |  |
| TR15165\|c0_g1_i2 | 4.21E-007 | 3.20 | d-arabinono- -lactone oxidase |
| TR11767\|c0_g1_i2 | 9.43E-009 | 3.20 | protein |
| TR14782\|c1_g1_i1 | 0.00E+000 | 3.20 |  |
| TR16132\|c0_g1_i5 | 7.24E-008 | 3.19 | homeodomain transcription factor |
| TR2519\|c0_g1_i1 | 2.22E-016 | 3.19 | hypothetical protein |
| TR11296\|c1_g1_i5 | 0.00E+000 | 3.18 |  |
| TR5240\|c0_g1_i3 | 0.00E+000 | 3.18 |  |
| TR15976\|c1_g1_i2 | 0.00E+000 | 3.18 |  |
| TR21904\|c0_g1_i1 | 7.23E-013 | 3.17 |  |
| TR10038\|c0_g2_i2 | 4.44E-016 | 3.16 |  |
| TR4075\|c0_g1_i1 | 3.30E-004 | 3.16 |  |
| TR13044\|c0_g1_i1 | 0.00E+000 | 3.15 | carnitine o-acetyltransferase |
| TR24613\|c0_g1_i2 | 1.15E-010 | 3.15 |  |
| TR10230\|c0_g1_i1 | 9.12E-004 | 3.15 |  |
| TR15972\|c5_g1_i4 | 0.00E+000 | 3.14 |  |
| TR24494\|c0_g1_i1 | 2.65E-003 | 3.14 |  |
| TR7190\|c2_g1_i2 | 0.00E+000 | 3.14 |  |
| TR1793\|c0_g2_i1 | 0.00E+000 | 3.13 |  |
| TR21471\|c0_g1_i1 | 0.00E+000 | 3.13 |  |
| TR9473\|c0_g1_i1 | 0.00E+000 | 3.13 |  |
| TR5213\|c0_g1_i1 | 3.79E-005 | 3.12 |  |
| TR22248\|c0_g1_i1 | 8.82E-003 | 3.12 |  |
| TR16263\|c0_g1_i5 | 0.00E+000 | 3.12 |  |
| TR7312\|c1_g1_i1 | 1.77E-005 | 3.11 | lactosylceramide 4-alpha-galactosyltransferase |
| TR10505\|c4_g2_i3 | 1.23E-003 | 3.11 |  |
| TR11324\|c1_g1_i2 | 3.18E-007 | 3.11 |  |
| TR20402\|c1_g1_i1 | 0.00E+000 | 3.11 |  |
| TR5568\|c0_g1_i4 | 0.00E+000 | 3.11 |  |
| TR10527\|c2_g3_i1 | 0.00E+000 | 3.11 | tkl dicty4 protein kinase |
| TR9318\|c0_g1_i1 | 5.73E-004 | 3.10 |  |
| TR4799\|c1_g1_i3 | 4.90E-003 | 3.10 |  |
| TR10881\|c0_g1_i1 | 2.37E-011 | 3.10 |  |
| TR12999\|c0_g1_i1 | 9.99E-016 | 3.09 |  |
| TR16412\|c0_g1_i1 | 0.00E+000 | 3.09 |  |
| TR25064\|c0_g1_i1 | 8.08E-010 | 3.08 |  |
| TR3124\|c1_g1_i1 | 0.00E+000 | 3.08 |  |
| TR3122\|c0_g2_i1 | 0.00E+000 | 3.08 |  |
| TR10382\|c1_g2_i2 | 2.96E-005 | 3.08 | protein |
| TR21014\|c0_g2_i1 | 1.51E-008 | 3.08 |  |
| TR2313\|c0_g3_i1 | 4.84E-005 | 3.07 |  |
| TR11830\|c0_g1_i1 | 0.00E+000 | 3.06 |  |
| TR2137\|c0_g1_i1 | 1.47E-007 | 3.06 | protein |
| TR17703\|c0_g2_i1 | 0.00E+000 | 3.06 |  |
| TR17689\|c0_g1_i1 | 0.00E+000 | 3.05 |  |
| TR11777\|c0_g1_i1 | 4.44E-016 | 3.05 |  |
| TR20043\|c0_g1_i1 | 0.00E+000 | 3.05 | tkl dicty4 protein kinase |
| TR1397\|c2_g1_i1 | 1.41E-006 | 3.04 |  |
| TR2285\|c2_g1_i2 | 1.11E-016 | 3.03 |  |
| TR13161\|c0_g1_i2 | 0.00E+000 | 3.03 |  |
| TR7190\|c2_g1_i3 | 0.00E+000 | 3.03 |  |
| TR2\|c1_g1_i1 | 0.00E+000 | 3.03 |  |
| TR19557\|c0_g1_i1 | 0.00E+000 | 3.03 | probable leucine-rich repeat receptor-like protein kinase at1g35710 |
| TR25310\|c0_g1_i1 | 0.00E+000 | 3.03 |  |
| TR8273\|c5_g3_i10 | 6.84E-011 | 3.02 |  |
| TR23683\|c0_g1_i1 | 0.00E+000 | 3.02 |  |
| TR5554\|c0_g4_i1 | 0.00E+000 | 3.02 |  |
| TR1520\|c0_g1_i1 | 2.54E-011 | 3.02 |  |
| TR21098\|c4_g3_i2 | 0.00E+000 | 3.02 | serine threonine-protein kinase ht1-like |
| TR3124\|c1_g1_i7 | 0.00E+000 | 3.01 |  |
| TR4619\|c1_g1_i3 | 9.29E-003 | 3.00 |  |
| TR1312\|c0_g1_i1 | 0.00E+000 | 3.00 |  |
| TR20344\|c0_g2_i2 | 0.00E+000 | 2.99 |  |
| TR19138\|c0_g1_i1 | 5.57E-008 | 2.99 | protein |
| TR19839\|c0_g1_i3 | 1.33E-015 | 2.99 |  |
| TR3461\|c0_g1_i2 | 0.00E+000 | 2.99 |  |
| TR12391\|c0_g1_i1 | 0.00E+000 | 2.98 | minichromosome maintenance protein 4 (cell division control protein 54) |
| TR8292\|c2_g1_i1 | 0.00E+000 | 2.98 |  |
| TR20843\|c0_g1_i1 | 0.00E+000 | 2.98 |  |
| TR11737\|c0_g2_i1 | 7.21E-011 | 2.98 |  |
| TR10442\|c0_g1_i1 | 3.31E-006 | 2.97 |  |
| TR2297\|c3_g3_i2 | 0.00E+000 | 2.97 | leucine-rich repeat receptor-like protein kinase family |
| TR24413\|c1_g1_i1 | 0.00E+000 | 2.97 |  |
| TR9148\|c0_g1_i1 | 0.00E+000 | 2.96 |  |
| TR25377\|c0_g1_i2 | 9.28E-003 | 2.96 |  |
| TR4672\|c0_g1_i2 | 8.88E-016 | 2.96 |  |
| TR3781\|c1_g2_i9 | 9.44E-005 | 2.95 |  |
| TR23553\|c0_g1_i1 | 0.00E+000 | 2.95 |  |
| TR6594\|c1_g1_i1 | 0.00E+000 | 2.95 |  |
| TR15986\|c0_g1_i10 | 3.14E-003 | 2.94 | peptide chain release factor 1 |
| TR3124\|c1_g1_i2 | 0.00E+000 | 2.94 |  |
| TR10530\|c1_g1_i9 | 0.00E+000 | 2.94 | silicon transporter |
| TR21098\|c4_g2_i1 | 0.00E+000 | 2.94 | tkl dicty4 protein kinase |
| TR11341\|c2_g2_i2 | 0.00E+000 | 2.94 |  |
| TR10885\|c0_g2_i3 | 0.00E+000 | 2.94 |  |
| TR24413\|c1_g1_i3 | 5.33E-009 | 2.93 |  |
| TR20344\|c0_g2_i1 | 0.00E+000 | 2.93 |  |
| TR16668\|c0_g3_i1 | 0.00E+000 | 2.93 | protein |
| TR20107\|c4_g1_i4 | 4.54E-005 | 2.93 |  |
| TR1438\|c2_g1_i2 | 0.00E+000 | 2.92 | protein |
| TR11563\|c0_g1_i1 | 0.00E+000 | 2.92 |  |
| TR8292\|c2_g1_i2 | 4.26E-009 | 2.91 |  |
| TR11740\|c0_g1_i1 | 0.00E+000 | 2.91 |  |
| TR22860\|c0_g1_i1 | 1.88E-013 | 2.90 |  |
| TR22079\|c0_g1_i1 | 0.00E+000 | 2.90 | nad -binding protein |
| TR16924\|c1_g1_i1 | 0.00E+000 | 2.89 |  |
| TR841\|c0_g1_i1 | 0.00E+000 | 2.89 |  |
| TR2\|c2_g1_i1 | 0.00E+000 | 2.89 |  |
| TR13161\|c0_g1_i1 | 0.00E+000 | 2.89 |  |
| TR20422\|c1_g2_i2 | 8.77E-003 | 2.89 | probable leucine-rich repeat receptor-like protein kinase at1g35710 |
| TR15565\|c0_g1_i5 | 1.14E-005 | 2.89 |  |
| TR5528\|c0_g1_i2 | 7.77E-005 | 2.89 |  |
| TR11290\|c0_g1_i1 | 0.00E+000 | 2.89 |  |
| TR25316\|c0_g1_i1 | 0.00E+000 | 2.89 |  |
| TR4278\|c0_g1_i2 | 0.00E+000 | 2.88 |  |
| TR6895\|c0_g1_i1 | 0.00E+000 | 2.87 |  |
| TR11354\|c0_g1_i2 | 1.05E-003 | 2.87 |  |
| TR16921\|c0_g1_i1 | 0.00E+000 | 2.87 |  |
| TR10462\|c0_g1_i1 | 0.00E+000 | 2.86 |  |
| TR10527\|c1_g1_i1 | 0.00E+000 | 2.86 |  |
| TR20051\|c5_g2_i1 | 2.56E-003 | 2.86 |  |
| TR22217\|c0_g1_i1 | 2.66E-010 | 2.85 |  |
| TR10486\|c4_g5_i12 | 4.68E-003 | 2.84 |  |
| TR15723\|c0_g1_i1 | 4.08E-003 | 2.84 |  |
| TR5568\|c0_g1_i1 | 5.80E-007 | 2.84 |  |
| TR20089\|c3_g1_i1 | 9.74E-009 | 2.84 |  |
| TR1436\|c1_g1_i4 | 5.53E-003 | 2.84 |  |
| TR7607\|c0_g1_i1 | 0.00E+000 | 2.84 | protein |
| TR10382\|c1_g3_i1 | 0.00E+000 | 2.84 | protein |
| TR24108\|c0_g1_i7 | 0.00E+000 | 2.83 | jumonji arid domain-containing protein 1a |
| TR10509\|c3_g2_i1 | 0.00E+000 | 2.82 | frustulin-like protein |
| TR10011\|c1_g1_i1 | 0.00E+000 | 2.82 | tkl dicty4 protein kinase |
| TR14431\|c1_g1_i1 | 6.33E-015 | 2.82 |  |
| TR10428\|c0_g1_i1 | 0.00E+000 | 2.81 | protein |
| TR25127\|c0_g2_i1 | 0.00E+000 | 2.81 | granzyme k-like |
| TR11357\|c2_g3_i1 | 0.00E+000 | 2.81 |  |
| TR11290\|c0_g1_i2 | 0.00E+000 | 2.81 |  |
| TR6580\|c1_g1_i1 | 2.26E-004 | 2.81 |  |
| TR8292\|c4_g1_i2 | 0.00E+000 | 2.80 |  |
| TR11557\|c0_g1_i1 | 1.72E-003 | 2.79 |  |
| TR12880\|c0_g1_i1 | 0.00E+000 | 2.78 |  |
| TR17400\|c0_g1_i1 | 2.05E-013 | 2.78 | protein |
| TR24628\|c0_g1_i2 | 6.72E-003 | 2.78 | uncharacterized aarf domain-containing protein kinase chloroplastic |
| TR7138\|c2_g1_i3 | 4.37E-013 | 2.78 |  |
| TR8292\|c4_g1_i1 | 0.00E+000 | 2.78 |  |
| TR20608\|c0_g2_i1 | 0.00E+000 | 2.78 |  |
| TR10382\|c0_g2_i1 | 0.00E+000 | 2.77 | protein |
| TR25990\|c0_g1_i1 | 0.00E+000 | 2.77 |  |
| TR2244\|c0_g2_i20 | 1.54E-013 | 2.77 |  |
| TR6646\|c3_g1_i8 | 2.37E-007 | 2.77 |  |
| TR12641\|c0_g1_i1 | 0.00E+000 | 2.77 |  |
| TR16263\|c0_g1_i3 | 0.00E+000 | 2.76 |  |
| TR5324\|c0_g1_i1 | 0.00E+000 | 2.76 |  |
| TR14516\|c0_g1_i1 | 1.11E-016 | 2.75 |  |
| TR6580\|c1_g2_i1 | 0.00E+000 | 2.74 | leucine rich repeat protein |
| TR23291\|c0_g1_i1 | 1.56E-003 | 2.74 | protein |
| TR2249\|c4_g2_i2 | 2.64E-006 | 2.74 |  |
| TR10527\|c2_g2_i1 | 0.00E+000 | 2.73 | tkl dicty4 protein kinase |
| TR9124\|c0_g2_i1 | 0.00E+000 | 2.73 |  |
| TR7124\|c0_g1_i2 | 0.00E+000 | 2.73 |  |
| TR3124\|c1_g1_i4 | 0.00E+000 | 2.73 |  |
| TR21055\|c0_g1_i1 | 0.00E+000 | 2.73 |  |
| TR7312\|c1_g1_i3 | 7.82E-011 | 2.72 | lactosylceramide 4-alpha-galactosyltransferase |
| TR10531\|c5_g2_i1 | 2.83E-010 | 2.72 | gag-pol polyprotein |
| TR6580\|c1_g2_i2 | 0.00E+000 | 2.71 | leucine rich repeat protein |
| TR1821\|c0_g1_i1 | 5.27E-004 | 2.71 |  |
| TR14743\|c0_g1_i1 | 1.00E-004 | 2.71 | frustulin-like protein |
| TR11507\|c0_g1_i1 | 5.59E-003 | 2.70 |  |
| TR25629\|c0_g2_i1 | 8.88E-009 | 2.70 |  |
| TR2187\|c0_g1_i1 | 0.00E+000 | 2.70 |  |
| TR12529\|c0_g1_i1 | 1.39E-003 | 2.70 |  |
| TR10556\|c8_g1_i7 | 9.50E-014 | 2.70 |  |
| TR8192\|c0_g1_i2 | 2.26E-006 | 2.70 | protein |
| TR19323\|c0_g1_i1 | 0.00E+000 | 2.70 | camk camk1 protein kinase |
| TR20106\|c0_g1_i1 | 0.00E+000 | 2.69 | lysine-specific histone demethylase 1 homolog 3 |
| TR7361\|c0_g1_i1 | 0.00E+000 | 2.69 |  |
| TR9797\|c0_g2_i1 | 5.45E-008 | 2.69 |  |
| TR17638\|c2_g1_i1 | 2.70E-006 | 2.69 |  |
| TR13236\|c0_g1_i1 | 0.00E+000 | 2.69 | tkl dicty4 drk protein kinase |
| TR1245\|c0_g1_i1 | 0.00E+000 | 2.69 |  |
| TR6658\|c3_g1_i5 | 1.71E-003 | 2.69 |  |
| TR1003\|c0_g1_i1 | 0.00E+000 | 2.69 | dnaj protein |
| TR23245\|c0_g1_i2 | 0.00E+000 | 2.69 |  |
| TR5914\|c0_g2_i1 | 1.74E-006 | 2.68 |  |
| TR17662\|c0_g1_i5 | 4.31E-005 | 2.68 | receptor-like protein kinase at3g47110 |
| TR5554\|c0_g2_i3 | 0.00E+000 | 2.68 |  |
| TR16905\|c0_g2_i1 | 2.90E-006 | 2.67 |  |
| TR403\|c0_g1_i1 | 0.00E+000 | 2.67 | protein |
| TR13453\|c0_g1_i1 | 0.00E+000 | 2.67 |  |
| TR19794\|c0_g1_i1 | 0.00E+000 | 2.67 |  |
| TR2249\|c0_g2_i1 | 2.40E-004 | 2.67 |  |
| TR19296\|c0_g1_i2 | 1.29E-006 | 2.67 |  |
| TR4783\|c0_g1_i1 | 0.00E+000 | 2.67 |  |
| TR23267\|c2_g4_i2 | 8.07E-004 | 2.66 |  |
| TR24661\|c0_g2_i1 | 7.72E-004 | 2.66 |  |
| TR12728\|c0_g1_i1 | 0.00E+000 | 2.66 |  |
| TR11093\|c0_g1_i1 | 0.00E+000 | 2.66 |  |
| TR16935\|c0_g1_i1 | 0.00E+000 | 2.66 |  |
| TR23067\|c0_g1_i1 | 2.00E-005 | 2.66 | protein |
| TR10527\|c2_g1_i6 | 0.00E+000 | 2.66 | tkl dicty4 protein kinase |
| TR21098\|c4_g3_i10 | 0.00E+000 | 2.65 | tkl dicty4 protein kinase |
| TR6609\|c1_g1_i1 | 1.19E-008 | 2.65 |  |
| TR3054\|c0_g1_i2 | 0.00E+000 | 2.65 |  |
| TR3769\|c0_g2_i2 | 1.08E-006 | 2.65 | sugar phosphate exchanger 3 |
| TR2296\|c2_g3_i5 | 0.00E+000 | 2.65 |  |
| TR7517\|c2_g1_i14 | 6.39E-003 | 2.64 |  |
| TR13291\|c1_g1_i1 | 0.00E+000 | 2.64 |  |
| TR10155\|c0_g1_i1 | 2.89E-007 | 2.63 |  |
| TR4299\|c0_g1_i2 | 1.74E-006 | 2.63 |  |
| TR1373\|c0_g1_i1 | 9.94E-005 | 2.63 |  |
| TR23223\|c4_g1_i10 | 1.87E-004 | 2.63 |  |
| TR8274\|c0_g1_i3 | 2.11E-013 | 2.62 |  |
| TR20967\|c0_g1_i2 | 3.87E-005 | 2.62 |  |
| TR24399\|c0_g1_i3 | 8.40E-007 | 2.62 |  |
| TR10382\|c1_g2_i1 | 1.10E-012 | 2.62 | protein |
| TR3746\|c1_g1_i1 | 0.00E+000 | 2.61 |  |
| TR8041\|c0_g1_i1 | 0.00E+000 | 2.61 |  |
| TR7971\|c0_g1_i2 | 1.38E-006 | 2.61 |  |
| TR10539\|c0_g1_i5 | 0.00E+000 | 2.61 | protein |
| TR14451\|c0_g1_i1 | 0.00E+000 | 2.61 |  |
| TR20384\|c1_g1_i2 | 0.00E+000 | 2.61 |  |
| TR24073\|c0_g1_i1 | 0.00E+000 | 2.61 |  |
| TR25998\|c0_g1_i1 | 0.00E+000 | 2.61 |  |
| TR2288\|c3_g1_i1 | 0.00E+000 | 2.60 |  |
| TR11777\|c0_g1_i4 | 0.00E+000 | 2.60 |  |
| TR10854\|c1_g4_i3 | 4.20E-005 | 2.60 | protein |
| TR10382\|c1_g1_i1 | 0.00E+000 | 2.60 | protein |
| TR8979\|c0_g1_i1 | 0.00E+000 | 2.60 |  |
| TR10142\|c0_g1_i1 | 0.00E+000 | 2.59 |  |
| TR25864\|c0_g1_i3 | 6.99E-014 | 2.59 |  |
| TR22101\|c0_g1_i1 | 4.91E-010 | 2.59 |  |
| TR7519\|c0_g3_i10 | 0.00E+000 | 2.59 |  |
| TR7645\|c0_g1_i1 | 2.47E-005 | 2.59 |  |
| TR25106\|c0_g1_i1 | 6.05E-003 | 2.58 |  |
| TR8292\|c4_g1_i3 | 0.00E+000 | 2.58 |  |
| TR5654\|c0_g2_i1 | 0.00E+000 | 2.58 |  |
| TR24661\|c0_g2_i2 | 0.00E+000 | 2.58 |  |
| TR276\|c0_g1_i1 | 4.39E-003 | 2.58 |  |
| TR11607\|c0_g1_i1 | 4.88E-015 | 2.58 | multi antimicrobial extrusion family protein |
| TR17185\|c0_g1_i1 | 0.00E+000 | 2.58 |  |
| TR10048\|c0_g1_i2 | 1.82E-012 | 2.58 |  |
| TR18562\|c2_g1_i2 | 0.00E+000 | 2.57 |  |
| TR14386\|c0_g1_i1 | 0.00E+000 | 2.57 |  |
| TR16905\|c1_g2_i3 | 1.58E-003 | 2.57 |  |
| TR19420\|c1_g1_i4 | 7.07E-004 | 2.57 |  |
| TR20093\|c2_g1_i1 | 0.00E+000 | 2.57 |  |
| TR22131\|c0_g1_i1 | 1.24E-004 | 2.56 |  |
| TR14451\|c0_g1_i3 | 4.84E-009 | 2.56 |  |
| TR20608\|c0_g1_i2 | 0.00E+000 | 2.56 |  |
| TR20384\|c1_g1_i1 | 0.00E+000 | 2.56 |  |
| TR7497\|c2_g4_i1 | 9.73E-004 | 2.55 |  |
| TR14430\|c0_g1_i3 | 0.00E+000 | 2.55 |  |
| TR24437\|c1_g1_i1 | 0.00E+000 | 2.55 |  |
| TR24252\|c0_g1_i2 | 1.11E-016 | 2.55 | protein |
| TR8255\|c0_g1_i1 | 4.21E-003 | 2.55 |  |
| TR10895\|c0_g1_i1 | 0.00E+000 | 2.54 |  |
| TR14794\|c1_g1_i4 | 2.29E-010 | 2.54 | protein |
| TR23303\|c4_g2_i3 | 7.50E-012 | 2.54 |  |
| TR18192\|c0_g1_i1 | 3.70E-010 | 2.54 |  |
| TR10650\|c0_g1_i1 | 0.00E+000 | 2.54 | protein |
| TR1492\|c4_g3_i2 | 1.43E-013 | 2.54 | protein |
| TR7138\|c2_g1_i2 | 0.00E+000 | 2.54 |  |
| TR9508\|c0_g1_i1 | 0.00E+000 | 2.53 |  |
| TR1289\|c0_g1_i1 | 7.28E-010 | 2.53 |  |
| TR12596\|c0_g1_i1 | 0.00E+000 | 2.53 |  |
| TR25750\|c0_g1_i1 | 0.00E+000 | 2.53 |  |
| TR1400\|c1_g1_i1 | 0.00E+000 | 2.53 |  |
| TR8292\|c3_g1_i2 | 0.00E+000 | 2.53 |  |
| TR17650\|c0_g1_i1 | 0.00E+000 | 2.53 | fructose-bisphosphate aldolase |
| TR12563\|c1_g1_i3 | 8.27E-005 | 2.52 |  |
| TR8554\|c0_g1_i1 | 0.00E+000 | 2.52 |  |
| TR10539\|c0_g1_i1 | 0.00E+000 | 2.52 | protein |
| TR11304\|c0_g1_i2 | 0.00E+000 | 2.52 | histidine-rich membrane protein ke4 homolog 2-like |
| TR22924\|c0_g1_i1 | 3.99E-006 | 2.51 |  |
| TR7616\|c0_g1_i1 | 0.00E+000 | 2.51 |  |
| TR1585\|c0_g3_i1 | 0.00E+000 | 2.50 | hypothetical protein GUITHDRAFT_161114 |
| TR11365\|c2_g1_i1 | 6.51E-004 | 2.50 | camp-dependent protein kinase type i-beta regulatory subunit |
| TR24397\|c2_g1_i1 | 8.41E-004 | 2.50 |  |
| TR25316\|c0_g1_i2 | 0.00E+000 | 2.50 |  |
| TR10507\|c7_g1_i1 | 0.00E+000 | 2.50 |  |
| TR25379\|c0_g3_i1 | 0.00E+000 | 2.49 |  |
| TR20529\|c0_g1_i1 | 8.96E-003 | 2.49 |  |
| TR10554\|c1_g10_i2 | 5.46E-003 | 2.49 |  |
| TR11328\|c1_g1_i3 | 7.80E-010 | 2.48 |  |
| TR10878\|c0_g1_i1 | 0.00E+000 | 2.48 |  |
| TR16263\|c0_g1_i6 | 9.69E-014 | 2.48 |  |
| TR7888\|c0_g1_i1 | 0.00E+000 | 2.48 |  |
| TR15502\|c0_g2_i1 | 0.00E+000 | 2.48 |  |
| TR9849\|c0_g2_i1 | 0.00E+000 | 2.47 |  |
| TR1448\|c0_g1_i6 | 1.57E-004 | 2.47 |  |
| TR21411\|c0_g1_i1 | 1.64E-008 | 2.47 |  |
| TR10567\|c0_g2_i1 | 6.21E-005 | 2.47 |  |
| TR1434\|c6_g1_i4 | 1.92E-011 | 2.46 |  |
| TR20122\|c1_g2_i11 | 4.64E-005 | 2.46 |  |
| TR13006\|c0_g2_i1 | 4.13E-010 | 2.46 |  |
| TR2279\|c1_g2_i2 | 4.19E-006 | 2.46 |  |
| TR20730\|c0_g1_i1 | 1.94E-010 | 2.46 |  |
| TR4641\|c0_g1_i2 | 0.00E+000 | 2.45 |  |
| TR11336\|c4_g1_i1 | 0.00E+000 | 2.45 |  |
| TR10120\|c0_g1_i1 | 0.00E+000 | 2.45 |  |
| TR1293\|c0_g1_i1 | 0.00E+000 | 2.45 | ion channel |
| TR890\|c0_g1_i1 | 0.00E+000 | 2.44 | grxc4_arath ame: full=glutaredoxin-c4 short= rxc4 |
| TR6616\|c2_g5_i8 | 0.00E+000 | 2.44 | fasciclin domain-containing protein |
| TR19288\|c0_g1_i1 | 3.42E-003 | 2.43 |  |
| TR16851\|c2_g2_i1 | 1.22E-011 | 2.43 |  |
| TR18712\|c0_g1_i1 | 0.00E+000 | 2.43 |  |
| TR12862\|c0_g1_i1 | 0.00E+000 | 2.43 | protein |
| TR24919\|c0_g1_i1 | 1.34E-004 | 2.43 |  |
| TR7190\|c2_g1_i1 | 0.00E+000 | 2.43 |  |
| TR3705\|c0_g1_i1 | 4.52E-003 | 2.43 |  |
| TR2481\|c0_g1_i1 | 3.91E-009 | 2.42 |  |
| TR2244\|c0_g2_i14 | 3.82E-004 | 2.42 |  |
| TR11684\|c0_g1_i1 | 0.00E+000 | 2.42 |  |
| TR1400\|c0_g1_i1 | 0.00E+000 | 2.42 |  |
| TR10560\|c6_g5_i7 | 1.34E-006 | 2.42 |  |
| TR3806\|c0_g1_i1 | 9.12E-003 | 2.42 |  |
| TR10487\|c0_g3_i3 | 5.28E-013 | 2.41 |  |
| TR8771\|c0_g2_i1 | 0.00E+000 | 2.41 |  |
| TR4626\|c0_g1_i1 | 0.00E+000 | 2.40 |  |
| TR5385\|c0_g2_i1 | 3.57E-012 | 2.40 | exosome complex component rrp41 |
| TR16301\|c0_g1_i1 | 0.00E+000 | 2.40 |  |
| TR10878\|c0_g1_i4 | 5.60E-004 | 2.39 |  |
| TR487\|c0_g1_i1 | 0.00E+000 | 2.39 |  |
| TR7480\|c1_g1_i4 | 2.42E-008 | 2.39 | goliath-related e3 ubiquitin ligase 1 |
| TR10500\|c2_g1_i2 | 0.00E+000 | 2.39 |  |
| TR7456\|c1_g1_i1 | 0.00E+000 | 2.39 | retinaldehyde binding protein 1 |
| TR5487\|c0_g1_i1 | 0.00E+000 | 2.39 |  |
| TR12514\|c1_g1_i1 | 0.00E+000 | 2.39 |  |
| TR7477\|c0_g1_i1 | 3.59E-006 | 2.39 |  |
| TR2285\|c2_g1_i6 | 5.19E-009 | 2.38 |  |
| TR2283\|c2_g8_i1 | 1.17E-010 | 2.38 |  |
| TR10530\|c1_g1_i8 | 0.00E+000 | 2.38 | silicon transporter |
| TR19903\|c1_g1_i1 | 1.09E-003 | 2.38 |  |
| TR24127\|c2_g1_i1 | 4.90E-005 | 2.38 |  |
| TR10624\|c0_g1_i1 | 0.00E+000 | 2.38 | hcr2- |
| TR8226\|c0_g1_i2 | 1.81E-004 | 2.37 |  |
| TR4810\|c0_g1_i2 | 4.44E-004 | 2.37 |  |
| TR11663\|c0_g1_i1 | 0.00E+000 | 2.37 |  |
| TR11341\|c6_g1_i2 | 5.05E-005 | 2.37 | protein |
| TR7182\|c3_g3_i1 | 0.00E+000 | 2.37 | protein |
| TR25893\|c1_g1_i1 | 0.00E+000 | 2.37 |  |
| TR11304\|c0_g1_i1 | 0.00E+000 | 2.37 | histidine-rich membrane protein ke4 homolog 2-like |
| TR8755\|c0_g1_i1 | 9.83E-013 | 2.37 |  |
| TR2295\|c1_g1_i8 | 9.61E-003 | 2.37 |  |
| TR19941\|c0_g3_i1 | 1.33E-010 | 2.36 |  |
| TR6658\|c3_g1_i3 | 0.00E+000 | 2.36 |  |
| TR2093\|c0_g1_i1 | 4.04E-004 | 2.36 |  |
| TR11324\|c1_g2_i1 | 3.17E-011 | 2.36 |  |
| TR16668\|c0_g1_i1 | 0.00E+000 | 2.36 | protein |
| TR12690\|c0_g1_i2 | 0.00E+000 | 2.35 |  |
| TR6658\|c1_g1_i1 | 0.00E+000 | 2.35 |  |
| TR6616\|c2_g5_i5 | 1.31E-005 | 2.34 | periostin-like isoform x2 |
| TR11281\|c3_g1_i1 | 1.03E-004 | 2.34 |  |
| TR1512\|c0_g1_i1 | 4.71E-006 | 2.34 |  |
| TR2337\|c4_g2_i1 | 1.68E-009 | 2.34 | protein |
| TR25589\|c0_g1_i1 | 1.42E-005 | 2.34 |  |
| TR23052\|c0_g1_i1 | 0.00E+000 | 2.34 |  |
| TR12494\|c0_g1_i1 | 4.66E-012 | 2.34 | 1-alkyl-2-acetylglycerophosphocholine esterase |
| TR21098\|c4_g3_i9 | 0.00E+000 | 2.33 | tkl dicty4 protein kinase |
| TR16014\|c1_g2_i1 | 2.45E-004 | 2.33 |  |
| TR19935\|c0_g1_i1 | 1.06E-004 | 2.33 |  |
| TR3744\|c4_g6_i2 | 1.58E-009 | 2.33 |  |
| TR10511\|c5_g4_i1 | 0.00E+000 | 2.33 | protein |
| TR16307\|c0_g1_i1 | 0.00E+000 | 2.32 |  |
| TR18605\|c0_g1_i1 | 0.00E+000 | 2.32 |  |
| TR8279\|c0_g1_i7 | 1.06E-005 | 2.32 |  |
| TR6609\|c1_g1_i3 | 2.05E-008 | 2.32 |  |
| TR25320\|c0_g1_i1 | 8.81E-003 | 2.31 |  |
| TR13459\|c0_g1_i1 | 0.00E+000 | 2.31 |  |
| TR7492\|c2_g1_i2 | 0.00E+000 | 2.31 | receptor-like protein kinase hsl1 |
| TR5240\|c0_g1_i1 | 0.00E+000 | 2.31 |  |
| TR17638\|c0_g1_i1 | 3.50E-003 | 2.31 |  |
| TR19941\|c0_g2_i2 | 0.00E+000 | 2.30 |  |
| TR6658\|c3_g1_i1 | 1.08E-007 | 2.30 |  |
| TR5864\|c0_g1_i1 | 0.00E+000 | 2.30 |  |
| TR19421\|c9_g1_i4 | 0.00E+000 | 2.30 |  |
| TR6660\|c2_g1_i1 | 0.00E+000 | 2.30 |  |
| TR17749\|c0_g1_i1 | 0.00E+000 | 2.30 | frustulin-like protein |
| TR1411\|c1_g1_i1 | 0.00E+000 | 2.29 |  |
| TR24656\|c0_g1_i1 | 5.27E-009 | 2.29 |  |
| TR15030\|c0_g1_i1 | 0.00E+000 | 2.29 |  |
| TR6455\|c0_g1_i1 | 0.00E+000 | 2.29 | peptidyl-prolyl cis-trans isomerase |
| TR7950\|c0_g1_i1 | 9.04E-005 | 2.29 |  |
| TR20080\|c1_g3_i2 | 0.00E+000 | 2.29 |  |
| TR2338\|c1_g2_i1 | 1.07E-004 | 2.29 |  |
| TR1520\|c0_g1_i2 | 9.42E-006 | 2.29 |  |
| TR10516\|c0_g1_i10 | 0.00E+000 | 2.29 | nitrate transporter |
| TR13152\|c0_g1_i1 | 3.65E-013 | 2.28 |  |
| TR9300\|c0_g1_i1 | 6.15E-006 | 2.28 | protein |
| TR4344\|c2_g1_i2 | 1.75E-006 | 2.28 |  |
| TR2330\|c1_g1_i1 | 9.09E-006 | 2.27 |  |
| TR11301\|c0_g1_i1 | 0.00E+000 | 2.27 |  |
| TR10186\|c0_g1_i3 | 1.39E-003 | 2.27 | gpi inositol-deacylase |
| TR4863\|c0_g1_i2 | 9.56E-007 | 2.27 |  |
| TR8758\|c0_g2_i1 | 3.67E-009 | 2.27 |  |
| TR19161\|c0_g2_i1 | 0.00E+000 | 2.27 |  |
| TR3084\|c5_g5_i1 | 0.00E+000 | 2.26 | protein |
| TR5854\|c0_g1_i1 | 0.00E+000 | 2.26 |  |
| TR7492\|c2_g1_i1 | 0.00E+000 | 2.26 | receptor-like protein kinase hsl1 |
| TR7162\|c5_g5_i1 | 9.83E-006 | 2.26 |  |
| TR2726\|c0_g1_i1 | 0.00E+000 | 2.26 | predicted protein |
| TR10530\|c1_g1_i13 | 0.00E+000 | 2.26 | silicon transporter |
| TR3093\|c0_g1_i2 | 3.33E-016 | 2.26 |  |
| TR18361\|c0_g1_i1 | 0.00E+000 | 2.25 |  |
| TR2505\|c0_g1_i1 | 6.89E-003 | 2.25 |  |
| TR1568\|c0_g1_i1 | 0.00E+000 | 2.25 |  |
| TR17198\|c0_g1_i1 | 2.09E-011 | 2.25 |  |
| TR7712\|c0_g1_i1 | 2.51E-005 | 2.25 |  |
| TR3148\|c0_g1_i1 | 0.00E+000 | 2.25 |  |
| TR3777\|c3_g1_i2 | 3.04E-003 | 2.25 |  |
| TR6658\|c2_g1_i1 | 0.00E+000 | 2.25 |  |
| TR16014\|c7_g1_i1 | 9.18E-006 | 2.25 |  |
| TR6611\|c1_g1_i2 | 6.89E-012 | 2.25 |  |
| TR21148\|c4_g2_i3 | 3.68E-005 | 2.24 |  |
| TR9563\|c0_g1_i1 | 0.00E+000 | 2.24 |  |
| TR16016\|c0_g1_i1 | 8.63E-006 | 2.24 |  |
| TR8825\|c0_g1_i1 | 8.87E-004 | 2.24 |  |
| TR13209\|c0_g1_i1 | 0.00E+000 | 2.24 |  |
| TR2711\|c0_g1_i1 | 0.00E+000 | 2.24 | syntaxin binding protein |
| TR22706\|c0_g1_i1 | 2.27E-003 | 2.24 |  |
| TR4207\|c1_g1_i1 | 1.07E-008 | 2.24 | protein |
| TR22159\|c0_g1_i1 | 0.00E+000 | 2.23 |  |
| TR25509\|c0_g1_i1 | 2.11E-012 | 2.23 | protein |
| TR10524\|c5_g1_i1 | 6.04E-005 | 2.23 |  |
| TR4619\|c1_g1_i1 | 0.00E+000 | 2.23 |  |
| TR23729\|c0_g1_i1 | 0.00E+000 | 2.23 |  |
| TR12072\|c1_g1_i1 | 0.00E+000 | 2.23 |  |
| TR7256\|c2_g1_i1 | 0.00E+000 | 2.23 |  |
| TR22152\|c0_g1_i1 | 0.00E+000 | 2.23 |  |
| TR7190\|c2_g2_i1 | 0.00E+000 | 2.22 |  |
| TR8348\|c0_g1_i1 | 0.00E+000 | 2.22 |  |
| TR11290\|c0_g2_i1 | 0.00E+000 | 2.22 | frustulin 5 |
| TR11321\|c5_g2_i9 | 3.66E-005 | 2.22 |  |
| TR17887\|c0_g1_i1 | 0.00E+000 | 2.22 |  |
| TR11457\|c0_g1_i1 | 0.00E+000 | 2.22 |  |
| TR23789\|c0_g1_i1 | 0.00E+000 | 2.22 | protein snf-2 |
| TR7138\|c2_g1_i7 | 0.00E+000 | 2.21 |  |
| TR13274\|c0_g1_i1 | 0.00E+000 | 2.21 |  |
| TR10940\|c0_g2_i1 | 0.00E+000 | 2.21 |  |
| TR12027\|c0_g1_i1 | 0.00E+000 | 2.21 |  |
| TR9849\|c0_g1_i1 | 0.00E+000 | 2.21 |  |
| TR25307\|c5_g1_i2 | 0.00E+000 | 2.21 |  |
| TR2300\|c1_g2_i1 | 0.00E+000 | 2.21 | protein |
| TR25989\|c0_g2_i1 | 6.69E-006 | 2.20 |  |
| TR4854\|c0_g1_i2 | 0.00E+000 | 2.20 |  |
| TR20914\|c0_g1_i1 | 5.86E-003 | 2.20 |  |
| TR170\|c0_g1_i1 | 6.93E-009 | 2.20 |  |
| TR5498\|c1_g1_i3 | 0.00E+000 | 2.20 |  |
| TR7037\|c0_g1_i1 | 0.00E+000 | 2.19 |  |
| TR7519\|c0_g3_i8 | 0.00E+000 | 2.19 |  |
| TR26008\|c0_g1_i1 | 9.47E-004 | 2.19 |  |
| TR2702\|c0_g1_i1 | 0.00E+000 | 2.19 |  |
| TR20941\|c0_g1_i1 | 2.52E-003 | 2.19 |  |
| TR1614\|c0_g1_i1 | 0.00E+000 | 2.19 |  |
| TR3738\|c3_g10_i1 | 0.00E+000 | 2.19 | oxidoreductase nad-binding domain-containing protein 1 |
| TR15446\|c0_g1_i1 | 0.00E+000 | 2.19 |  |
| TR12175\|c0_g1_i1 | 5.71E-003 | 2.19 |  |
| TR7049\|c0_g1_i1 | 3.75E-003 | 2.19 | protein |
| TR15821\|c0_g1_i1 | 0.00E+000 | 2.19 |  |
| TR16263\|c0_g1_i1 | 0.00E+000 | 2.18 |  |
| TR21155\|c0_g1_i1 | 0.00E+000 | 2.18 |  |
| TR2044\|c0_g1_i3 | 0.00E+000 | 2.18 |  |
| TR2244\|c0_g2_i17 | 3.71E-008 | 2.18 |  |
| TR13906\|c0_g1_i1 | 0.00E+000 | 2.18 |  |
| TR11791\|c2_g1_i1 | 8.28E-009 | 2.18 |  |
| TR23516\|c0_g1_i2 | 2.73E-005 | 2.18 |  |
| TR20284\|c0_g1_i1 | 1.99E-008 | 2.18 |  |
| TR20384\|c0_g1_i2 | 0.00E+000 | 2.17 |  |
| TR9154\|c0_g2_i1 | 3.95E-006 | 2.17 | cell division cycle cofactor-apc complex |
| TR15252\|c0_g1_i1 | 1.08E-004 | 2.17 |  |
| TR3141\|c3_g1_i1 | 0.00E+000 | 2.17 |  |
| TR1260\|c0_g1_i2 | 7.94E-011 | 2.17 |  |
| TR22133\|c0_g1_i1 | 0.00E+000 | 2.17 | hco3 transporter |
| TR25089\|c0_g1_i1 | 1.33E-015 | 2.17 | tkl dicty4 protein kinase |
| TR20128\|c0_g1_i2 | 0.00E+000 | 2.17 |  |
| TR7519\|c0_g3_i13 | 6.77E-015 | 2.17 |  |
| TR10530\|c1_g1_i1 | 0.00E+000 | 2.17 | silicon transporter |
| TR2330\|c1_g1_i3 | 0.00E+000 | 2.17 |  |
| TR4294\|c0_g1_i1 | 1.18E-004 | 2.17 |  |
| TR16440\|c0_g1_i1 | 0.00E+000 | 2.16 |  |
| TR16790\|c0_g1_i1 | 0.00E+000 | 2.16 |  |
| TR5851\|c0_g1_i1 | 0.00E+000 | 2.16 |  |
| TR22245\|c0_g1_i1 | 0.00E+000 | 2.16 |  |
| TR9423\|c0_g1_i1 | 0.00E+000 | 2.16 |  |
| TR2337\|c3_g3_i1 | 2.95E-014 | 2.16 |  |
| TR5708\|c0_g1_i1 | 0.00E+000 | 2.15 |  |
| TR8400\|c0_g1_i1 | 0.00E+000 | 2.15 |  |
| TR23518\|c0_g1_i1 | 0.00E+000 | 2.15 |  |
| TR5509\|c0_g1_i2 | 1.24E-010 | 2.15 | atp-dependent -nad h-hydrate partial |
| TR19707\|c0_g1_i1 | 0.00E+000 | 2.15 |  |
| TR22081\|c0_g1_i1 | 0.00E+000 | 2.14 |  |
| TR21114\|c2_g3_i1 | 0.00E+000 | 2.14 |  |
| TR10519\|c4_g1_i1 | 2.39E-013 | 2.14 |  |
| TR18639\|c3_g2_i11 | 1.16E-011 | 2.14 | tkl dicty4 protein kinase |
| TR10559\|c3_g2_i4 | 0.00E+000 | 2.14 | fucosyltransferase-like protein |
| TR821\|c0_g1_i1 | 3.95E-009 | 2.14 | protein |
| TR18624\|c0_g1_i1 | 0.00E+000 | 2.14 | laminin domain-containing 2 |
| TR7503\|c1_g1_i3 | 1.64E-006 | 2.14 |  |
| TR11341\|c5_g1_i1 | 0.00E+000 | 2.14 |  |
| TR16003\|c2_g7_i3 | 0.00E+000 | 2.13 | endo- -beta-glucosidase |
| TR6635\|c3_g1_i2 | 1.89E-006 | 2.13 |  |
| TR10854\|c1_g2_i1 | 9.01E-013 | 2.13 | protein |
| TR6936\|c0_g1_i3 | 5.99E-004 | 2.13 |  |
| TR6581\|c2_g1_i6 | 2.70E-005 | 2.13 |  |
| TR11910\|c0_g1_i1 | 0.00E+000 | 2.13 |  |
| TR21788\|c0_g1_i1 | 0.00E+000 | 2.13 |  |
| TR10484\|c1_g1_i8 | 7.53E-006 | 2.12 | myosin heavy partial |
| TR24274\|c0_g1_i1 | 2.55E-012 | 2.12 | mini-chromosome maintenance complex-binding |
| TR14717\|c7_g1_i1 | 4.43E-007 | 2.12 |  |
| TR12083\|c1_g1_i3 | 1.73E-007 | 2.12 |  |
| TR10103\|c0_g1_i1 | 2.31E-006 | 2.12 |  |
| TR20123\|c3_g1_i10 | 6.94E-003 | 2.12 |  |
| TR7209\|c1_g1_i4 | 5.66E-015 | 2.12 | tkl dicty4 protein kinase |
| TR25850\|c0_g1_i1 | 2.11E-013 | 2.12 |  |
| TR10922\|c1_g1_i1 | 3.44E-007 | 2.12 |  |
| TR21339\|c0_g1_i1 | 0.00E+000 | 2.12 | carbonyl reductase |
| TR10527\|c2_g1_i7 | 0.00E+000 | 2.11 | tkl dicty4 protein kinase |
| TR3497\|c0_g1_i1 | 8.51E-009 | 2.11 |  |
| TR24333\|c0_g1_i1 | 1.05E-012 | 2.11 |  |
| TR2299\|c0_g2_i1 | 2.07E-007 | 2.11 |  |
| TR6482\|c0_g1_i2 | 0.00E+000 | 2.11 | predicted protein |
| TR7144\|c7_g1_i3 | 0.00E+000 | 2.11 | frustulin-like protein |
| TR12083\|c1_g1_i1 | 4.00E-005 | 2.11 |  |
| TR18633\|c0_g1_i5 | 4.03E-003 | 2.11 | protein |
| TR21113\|c9_g8_i2 | 4.59E-006 | 2.11 | tkl dicty4 protein kinase |
| TR11324\|c1_g2_i3 | 7.10E-004 | 2.10 |  |
| TR10312\|c0_g1_i1 | 7.77E-016 | 2.10 | protein |
| TR16979\|c0_g1_i1 | 1.55E-010 | 2.10 |  |
| TR5782\|c0_g1_i1 | 2.35E-007 | 2.10 |  |
| TR20101\|c3_g1_i7 | 1.77E-003 | 2.10 | protein |
| TR7982\|c0_g1_i1 | 1.24E-004 | 2.10 |  |
| TR7162\|c5_g5_i2 | 1.67E-005 | 2.10 |  |
| TR3828\|c0_g1_i1 | 0.00E+000 | 2.10 |  |
| TR18024\|c0_g1_i1 | 4.21E-004 | 2.10 |  |
| TR14717\|c10_g2_i2 | 4.85E-010 | 2.10 |  |
| TR14382\|c0_g1_i2 | 4.50E-004 | 2.09 |  |
| TR2244\|c0_g2_i5 | 0.00E+000 | 2.09 |  |
| TR1856\|c0_g1_i1 | 0.00E+000 | 2.09 | protein |
| TR9275\|c0_g1_i1 | 0.00E+000 | 2.09 |  |
| TR4510\|c0_g1_i1 | 6.73E-014 | 2.08 |  |
| TR13649\|c0_g1_i1 | 0.00E+000 | 2.08 | leucine-rich repeat receptor-like protein kinase family |
| TR6762\|c0_g1_i1 | 2.22E-016 | 2.08 | protein |
| TR9379\|c0_g1_i1 | 0.00E+000 | 2.08 | ankyrin repeat domain-containing protein 50-like |
| TR12520\|c1_g1_i1 | 1.23E-003 | 2.08 | myosin- partial |
| TR3614\|c0_g1_i1 | 0.00E+000 | 2.08 |  |
| TR8282\|c0_g1_i1 | 0.00E+000 | 2.07 |  |
| TR5179\|c0_g1_i1 | 1.11E-016 | 2.07 | rna polymerase rpb1 repeat containing protein |
| TR1017\|c0_g1_i2 | 8.08E-003 | 2.07 |  |
| TR11808\|c0_g1_i1 | 0.00E+000 | 2.07 |  |
| TR20293\|c0_g1_i1 | 1.79E-008 | 2.07 |  |
| TR19822\|c0_g1_i1 | 0.00E+000 | 2.07 |  |
| TR7162\|c4_g3_i1 | 0.00E+000 | 2.07 |  |
| TR14717\|c10_g2_i1 | 1.44E-010 | 2.07 |  |
| TR6574\|c0_g1_i5 | 1.21E-006 | 2.06 | predicted protein |
| TR23279\|c4_g1_i17 | 1.93E-003 | 2.06 | protein |
| TR11298\|c0_g1_i1 | 1.11E-016 | 2.06 | rna polymerase rpb1 repeat containing protein |
| TR4664\|c0_g1_i1 | 0.00E+000 | 2.06 |  |
| TR2066\|c0_g1_i1 | 0.00E+000 | 2.06 |  |
| TR3588\|c0_g1_i1 | 5.19E-007 | 2.06 | pseudouridylate synthase |
| TR16287\|c0_g3_i1 | 0.00E+000 | 2.06 |  |
| TR1421\|c0_g1_i2 | 3.01E-003 | 2.06 | protein |
| TR20110\|c0_g1_i2 | 0.00E+000 | 2.06 |  |
| TR20175\|c0_g1_i1 | 0.00E+000 | 2.06 |  |
| TR9235\|c0_g1_i1 | 0.00E+000 | 2.06 |  |
| TR3755\|c4_g5_i1 | 0.00E+000 | 2.06 |  |
| TR11034\|c0_g1_i1 | 0.00E+000 | 2.05 |  |
| TR5554\|c0_g1_i1 | 1.80E-004 | 2.05 |  |
| TR20584\|c0_g2_i1 | 1.19E-005 | 2.05 |  |
| TR10559\|c2_g1_i1 | 0.00E+000 | 2.05 |  |
| TR13378\|c0_g1_i1 | 0.00E+000 | 2.05 | triosephosphate isomerase |
| TR20043\|c1_g1_i5 | 0.00E+000 | 2.05 |  |
| TR18618\|c0_g1_i1 | 0.00E+000 | 2.04 |  |
| TR15089\|c0_g1_i1 | 0.00E+000 | 2.04 |  |
| TR19947\|c12_g6_i1 | 0.00E+000 | 2.04 |  |
| TR9737\|c0_g1_i1 | 0.00E+000 | 2.04 |  |
| TR15972\|c6_g6_i1 | 2.05E-012 | 2.04 |  |
| TR7346\|c2_g1_i1 | 2.84E-003 | 2.04 |  |
| TR20065\|c6_g9_i1 | 0.00E+000 | 2.03 |  |
| TR7451\|c2_g1_i2 | 1.74E-014 | 2.03 |  |
| TR7188\|c2_g1_i1 | 6.60E-003 | 2.03 | protein |
| TR21916\|c0_g1_i1 | 0.00E+000 | 2.03 |  |
| TR21782\|c0_g1_i2 | 0.00E+000 | 2.03 | 5-hydroxyisourate hydrolase-like |
| TR19421\|c9_g1_i1 | 0.00E+000 | 2.03 |  |
| TR3110\|c5_g1_i2 | 4.31E-003 | 2.02 |  |
| TR2315\|c9_g5_i2 | 0.00E+000 | 2.02 |  |
| TR18144\|c0_g1_i1 | 0.00E+000 | 2.02 | histone h2b |
| TR20403\|c4_g3_i4 | 0.00E+000 | 2.02 | lrr-gtpase of the roco family |
| TR21918\|c0_g2_i1 | 0.00E+000 | 2.02 |  |
| TR8611\|c0_g1_i1 | 0.00E+000 | 2.02 |  |
| TR7519\|c0_g8_i1 | 0.00E+000 | 2.02 | epsilon frustilin |
| TR19916\|c0_g1_i1 | 1.44E-009 | 2.02 |  |
| TR7138\|c2_g1_i5 | 6.33E-003 | 2.02 |  |
| TR15113\|c0_g1_i1 | 0.00E+000 | 2.02 |  |
| TR8387\|c0_g1_i1 | 0.00E+000 | 2.02 | elongation factor 3-like protein abcf transporter family |
| TR13050\|c0_g1_i1 | 0.00E+000 | 2.01 |  |
| TR20299\|c0_g1_i1 | 1.11E-016 | 2.01 | dead deah box rna |
| TR20238\|c0_g1_i1 | 2.21E-008 | 2.01 |  |
| TR3054\|c1_g4_i2 | 4.41E-005 | 2.01 |  |
| TR8292\|c3_g1_i1 | 0.00E+000 | 2.01 |  |
| TR20260\|c0_g1_i1 | 0.00E+000 | 2.01 |  |
| TR24600\|c0_g1_i1 | 0.00E+000 | 2.01 |  |
| TR4253\|c1_g1_i3 | 1.54E-003 | 2.00 | protein |
| TR21853\|c0_g1_i1 | 1.93E-004 | 2.00 | 2-on-2 hemoglobin |
| TR10499\|c0_g1_i1 | 4.95E-004 | 2.00 |  |
| TR9269\|c0_g1_i2 | 2.29E-013 | 2.00 |  |
| TR10909\|c0_g2_i2 | 7.73E-004 | 2.00 |  |
| TR18643\|c0_g1_i2 | 0.00E+000 | 2.00 |  |
| TR8680\|c0_g1_i1 | 0.00E+000 | 2.00 |  |
| TR22046\|c0_g1_i1 | 0.00E+000 | 2.00 |  |
| TR16003\|c1_g1_i1 | 0.00E+000 | 2.00 |  |
| TR14433\|c0_g1_i1 | 0.00E+000 | 2.00 | protein |
| TR3103\|c1_g1_i2 | 0.00E+000 | -20.17 |  |
| TR842\|c0_g1_i2 | 0.00E+000 | -19.77 |  |
| TR489\|c0_g1_i5 | 0.00E+000 | -19.76 |  |
| TR1297\|c0_g2_i2 | 0.00E+000 | -19.42 | vacuolar iron family transporter |
| TR19390\|c3_g1_i2 | 3.11E-012 | -18.88 |  |
| TR7183\|c1_g1_i1 | 7.35E-009 | -18.73 |  |
| TR3524\|c0_g2_i1 | 1.51E-012 | -18.69 |  |
| TR20842\|c0_g2_i1 | 9.21E-004 | -18.54 |  |
| TR20351\|c0_g1_i4 | 6.48E-005 | -18.53 | sodium bile acid cotransporter 7 isoform x1 |
| TR6703\|c0_g2_i1 | 5.00E-005 | -18.52 |  |
| TR9205\|c0_g2_i1 | 2.10E-003 | -18.52 |  |
| TR6591\|c1_g1_i3 | 0.00E+000 | -18.44 | protein |
| TR8362\|c0_g1_i1 | 6.97E-009 | -18.35 | abc transporter |
| TR20834\|c0_g1_i1 | 7.53E-008 | -18.25 |  |
| TR489\|c0_g1_i2 | 7.00E-004 | -18.13 |  |
| TR1530\|c0_g1_i1 | 2.28E-005 | -18.06 |  |
| TR21150\|c0_g1_i5 | 1.01E-003 | -17.76 | abc transporter |
| TR14356\|c0_g1_i1 | 5.83E-003 | -17.58 |  |
| TR17546\|c0_g1_i1 | 3.35E-003 | -17.15 |  |
| TR19919\|c0_g1_i1 | 2.49E-008 | -16.89 |  |
| TR21829\|c0_g1_i3 | 5.39E-005 | -16.53 | protein |
| TR4462\|c0_g1_i2 | 2.47E-012 | -16.25 |  |
| TR16087\|c0_g1_i1 | 3.57E-003 | -16.04 | topoisomerase 6 subunit b |
| TR8579\|c0_g1_i7 | 2.36E-003 | -15.15 | predicted protein |
| TR19710\|c0_g1_i4 | 6.22E-015 | -14.73 | hypothetical protein THAOC_32472 |
| TR16304\|c0_g1_i2 | 8.18E-003 | -14.59 | predicted protein |
| TR811\|c0_g1_i1 | 0.00E+000 | -14.18 | protein |
| TR10516\|c0_g1_i9 | 7.67E-003 | -14.02 | nitrate transporter |
| TR6647\|c0_g1_i3 | 0.00E+000 | -13.64 | diacylglycerol acyltransferase type 2a |
| TR13441\|c0_g1_i1 | 0.00E+000 | -12.89 |  |
| TR15978\|c1_g1_i1 | 2.49E-005 | -12.83 |  |
| TR4345\|c0_g1_i1 | 1.43E-004 | -12.73 |  |
| TR7426\|c0_g1_i1 | 7.84E-005 | -12.47 |  |
| TR19710\|c0_g1_i7 | 1.44E-015 | -12.46 |  |
| TR21112\|c1_g1_i9 | 7.48E-004 | -12.34 |  |
| TR19710\|c0_g1_i2 | 0.00E+000 | -12.27 | hnh endonuclease family protein |
| TR10560\|c6_g5_i10 | 2.71E-003 | -11.90 |  |
| TR56\|c0_g1_i1 | 1.82E-005 | -11.63 |  |
| TR8004\|c0_g1_i1 | 2.20E-013 | -11.58 |  |
| TR56\|c0_g1_i4 | 2.54E-004 | -11.11 |  |
| TR4798\|c0_g2_i2 | 1.35E-009 | -10.64 |  |
| TR7217\|c0_g1_i5 | 1.78E-003 | -10.36 | protein |
| TR2772\|c0_g1_i1 | 3.87E-003 | -10.18 |  |
| TR7517\|c2_g1_i19 | 2.91E-010 | -10.12 |  |
| TR10560\|c6_g5_i9 | 0.00E+000 | -9.96 |  |
| TR18473\|c0_g1_i1 | 8.80E-004 | -9.93 |  |
| TR11374\|c0_g1_i1 | 1.71E-014 | -9.71 | protein |
| TR10209\|c0_g1_i3 | 2.80E-003 | -9.55 |  |
| TR20351\|c0_g1_i1 | 0.00E+000 | -9.45 | protein |
| TR25491\|c0_g1_i1 | 2.84E-003 | -9.06 |  |
| TR3720\|c0_g2_i6 | 1.44E-007 | -8.91 |  |
| TR2226\|c0_g1_i3 | 3.12E-004 | -8.85 |  |
| TR2309\|c0_g1_i3 | 4.12E-003 | -8.84 |  |
| TR1013\|c0_g1_i1 | 1.76E-006 | -8.56 | s-adenosylmethionine mitochondrial carrier protein |
| TR4372\|c0_g1_i1 | 0.00E+000 | -8.44 |  |
| TR7325\|c0_g2_i2 | 6.02E-004 | -8.22 | glutathione s-transferase |
| TR3388\|c0_g1_i1 | 0.00E+000 | -8.20 |  |
| TR17223\|c0_g2_i1 | 8.72E-003 | -8.19 | helicase partial |
| TR20531\|c0_g1_i2 | 7.28E-006 | -8.09 | protein |
| TR5346\|c0_g1_i1 | 0.00E+000 | -8.07 |  |
| TR21876\|c0_g1_i1 | 6.77E-015 | -7.84 |  |
| TR3120\|c0_g1_i2 | 0.00E+000 | -7.80 |  |
| TR7171\|c1_g7_i2 | 1.25E-006 | -7.75 |  |
| TR6649\|c1_g1_i5 | 0.00E+000 | -7.66 | probable inactive purple acid phosphatase 16 |
| TR10922\|c1_g2_i2 | 0.00E+000 | -7.65 |  |
| TR13681\|c0_g1_i5 | 1.14E-003 | -7.42 |  |
| TR7877\|c0_g1_i1 | 0.00E+000 | -7.39 |  |
| TR22975\|c0_g1_i2 | 0.00E+000 | -7.27 | arsenite-translocating family |
| TR4372\|c0_g1_i3 | 0.00E+000 | -7.24 |  |
| TR25114\|c0_g1_i3 | 1.29E-004 | -7.21 |  |
| TR10560\|c6_g5_i2 | 4.17E-007 | -7.09 |  |
| TR10922\|c1_g2_i1 | 0.00E+000 | -6.98 |  |
| TR10922\|c2_g1_i1 | 0.00E+000 | -6.97 |  |
| TR25902\|c0_g1_i2 | 0.00E+000 | -6.94 | isocitrate lyase |
| TR10922\|c1_g2_i3 | 0.00E+000 | -6.94 |  |
| TR6297\|c0_g1_i1 | 0.00E+000 | -6.77 |  |
| TR239\|c0_g1_i2 | 4.18E-004 | -6.64 |  |
| TR4372\|c0_g1_i2 | 0.00E+000 | -6.61 |  |
| TR12496\|c0_g1_i3 | 1.63E-003 | -6.59 |  |
| TR489\|c0_g2_i1 | 0.00E+000 | -6.50 |  |
| TR3103\|c1_g1_i1 | 0.00E+000 | -6.34 | mucin-like protein |
| TR10183\|c0_g1_i2 | 3.60E-007 | -6.24 |  |
| TR500\|c0_g1_i5 | 1.39E-003 | -6.22 |  |
| TR1286\|c0_g1_i1 | 0.00E+000 | -6.20 |  |
| TR23497\|c0_g1_i2 | 0.00E+000 | -6.18 | protein |
| TR21112\|c1_g1_i6 | 8.25E-003 | -6.13 |  |
| TR849\|c0_g1_i2 | 2.50E-006 | -6.09 |  |
| TR15508\|c0_g1_i2 | 2.56E-003 | -6.09 | camk cdpk protein kinase |
| TR15860\|c0_g1_i1 | 0.00E+000 | -6.01 |  |
| TR24121\|c0_g1_i4 | 0.00E+000 | -5.97 |  |
| TR15972\|c6_g2_i2 | 1.11E-006 | -5.97 |  |
| TR4638\|c0_g1_i1 | 0.00E+000 | -5.93 |  |
| TR5507\|c0_g1_i1 | 1.55E-010 | -5.91 |  |
| TR1088\|c0_g1_i2 | 2.86E-003 | -5.84 |  |
| TR193\|c0_g1_i2 | 3.39E-003 | -5.78 |  |
| TR6580\|c0_g1_i3 | 0.00E+000 | -5.71 |  |
| TR15492\|c0_g1_i1 | 0.00E+000 | -5.70 |  |
| TR24244\|c0_g2_i1 | 6.92E-005 | -5.69 | transmembrane protein with metallophosphoesterase domain |
| TR20123\|c1_g2_i1 | 0.00E+000 | -5.59 |  |
| TR13656\|c0_g1_i1 | 5.44E-007 | -5.58 |  |
| TR1452\|c0_g1_i3 | 1.49E-003 | -5.56 |  |
| TR20085\|c0_g2_i11 | 8.76E-005 | -5.55 |  |
| TR16994\|c0_g1_i1 | 0.00E+000 | -5.53 | abc transporter |
| TR1469\|c4_g1_i1 | 2.69E-014 | -5.53 | atp-dependent zinc metalloprotease ftsh chloroplastic |
| TR17479\|c0_g2_i1 | 0.00E+000 | -5.49 |  |
| TR18518\|c0_g1_i1 | 7.57E-005 | -5.38 |  |
| TR19423\|c0_g2_i2 | 0.00E+000 | -5.38 |  |
| TR1525\|c0_g1_i5 | 1.96E-003 | -5.30 |  |
| TR489\|c0_g1_i4 | 0.00E+000 | -5.28 |  |
| TR2129\|c1_g1_i1 | 0.00E+000 | -5.27 |  |
| TR4220\|c0_g1_i3 | 0.00E+000 | -5.27 |  |
| TR10705\|c0_g1_i1 | 0.00E+000 | -5.25 | lipid phosphate phosphatase 1 |
| TR25044\|c0_g1_i3 | 7.89E-011 | -5.24 | protein |
| TR20123\|c2_g1_i1 | 0.00E+000 | -5.14 |  |
| TR4620\|c0_g1_i3 | 1.85E-006 | -5.11 |  |
| TR24488\|c0_g1_i1 | 4.17E-008 | -5.11 |  |
| TR19105\|c0_g2_i1 | 4.79E-010 | -5.10 | triose phosphate phosphate translocator |
| TR12496\|c0_g1_i2 | 0.00E+000 | -5.08 |  |
| TR22933\|c0_g1_i1 | 1.47E-009 | -5.03 |  |
| TR23034\|c0_g1_i1 | 9.33E-015 | -5.02 | probable cytosolic iron-sulfur protein assembly protein 1 q0usg2 |
| TR24131\|c0_g3_i1 | 2.65E-014 | -5.00 |  |
| TR25294\|c0_g1_i5 | 1.80E-003 | -5.00 |  |
| TR21106\|c3_g1_i1 | 4.71E-005 | -5.00 | protein |
| TR18883\|c0_g1_i3 | 0.00E+000 | -5.00 | methylcrotonoyl- carboxylase beta mitochondrial |
| TR15158\|c0_g1_i2 | 0.00E+000 | -4.95 | gtp binding protein |
| TR1311\|c0_g1_i1 | 9.55E-009 | -4.93 | multidrug resistance protein homolog 49-like |
| TR5154\|c0_g1_i4 | 0.00E+000 | -4.89 | 2-oxoisovalerate dehydrogenase subunit mitochondrial |
| TR10477\|c0_g1_i5 | 3.33E-003 | -4.89 |  |
| TR5331\|c0_g1_i1 | 0.00E+000 | -4.88 |  |
| TR7745\|c0_g1_i1 | 2.39E-012 | -4.87 | protein |
| TR10873\|c0_g2_i2 | 9.06E-003 | -4.87 | agc pka protein kinase |
| TR11309\|c0_g1_i6 | 1.72E-009 | -4.86 | wd repeat domain phosphoinositide-interacting protein 3 |
| TR7897\|c0_g1_i1 | 8.93E-013 | -4.86 |  |
| TR12433\|c0_g1_i1 | 2.16E-005 | -4.86 | protein |
| TR4652\|c0_g1_i1 | 0.00E+000 | -4.86 |  |
| TR3777\|c3_g1_i1 | 2.30E-007 | -4.84 |  |
| TR2249\|c2_g1_i1 | 0.00E+000 | -4.83 |  |
| TR22713\|c0_g1_i1 | 0.00E+000 | -4.83 | protein creg1 |
| TR15492\|c0_g2_i1 | 6.27E-004 | -4.82 |  |
| TR1414\|c1_g2_i1 | 5.89E-003 | -4.80 | xs20e_neopa ame: full=bifunctional acetylxylan esterase xylanase 20e includes: ame: full=acetylxylan esterase includes: ame: full=endo- -beta-xylanase short=xylanase ame: full= -beta-d-xylan xylanohydrolase flags: precursor |
| TR5224\|c0_g1_i2 | 5.91E-004 | -4.79 |  |
| TR10183\|c0_g1_i1 | 0.00E+000 | -4.79 |  |
| TR4725\|c0_g1_i1 | 0.00E+000 | -4.78 |  |
| TR16033\|c0_g1_i1 | 9.01E-003 | -4.77 | histone deacetylase 6 |
| TR13336\|c0_g1_i1 | 0.00E+000 | -4.76 |  |
| TR7949\|c0_g2_i3 | 8.26E-010 | -4.75 | arylsulfatase g |
| TR489\|c0_g1_i3 | 0.00E+000 | -4.75 |  |
| TR8270\|c0_g1_i2 | 1.19E-010 | -4.75 |  |
| TR18350\|c0_g1_i1 | 2.85E-004 | -4.68 |  |
| TR21598\|c0_g1_i1 | 0.00E+000 | -4.67 |  |
| TR4300\|c0_g1_i1 | 0.00E+000 | -4.67 |  |
| TR19710\|c0_g1_i5 | 0.00E+000 | -4.65 | hnh endonuclease family protein |
| TR1443\|c0_g1_i2 | 0.00E+000 | -4.65 | divalent anion:na+ symporter family |
| TR2249\|c3_g1_i1 | 8.24E-008 | -4.64 |  |
| TR25636\|c0_g1_i2 | 1.59E-007 | -4.56 | choline transporter-like protein |
| TR21379\|c0_g1_i1 | 0.00E+000 | -4.56 | calreticulin |
| TR18573\|c0_g2_i5 | 5.80E-009 | -4.55 | protein |
| TR17893\|c0_g1_i1 | 9.99E-016 | -4.54 |  |
| TR19647\|c0_g1_i1 | 0.00E+000 | -4.50 |  |
| TR12526\|c0_g2_i1 | 0.00E+000 | -4.50 |  |
| TR20204\|c1_g1_i1 | 4.23E-005 | -4.50 | protein |
| TR259\|c0_g1_i1 | 3.17E-005 | -4.42 | high mobility group protein |
| TR16097\|c0_g1_i3 | 1.32E-003 | -4.41 | lipid transfer protein 5 |
| TR3738\|c3_g3_i1 | 0.00E+000 | -4.41 |  |
| TR10729\|c0_g1_i1 | 5.79E-006 | -4.40 |  |
| TR7998\|c0_g2_i2 | 0.00E+000 | -4.39 |  |
| TR15101\|c0_g1_i2 | 2.51E-006 | -4.38 | predicted protein |
| TR3124\|c1_g1_i5 | 4.07E-012 | -4.35 |  |
| TR16025\|c0_g1_i7 | 1.62E-007 | -4.35 | alpha beta hydrolase domain-containing protein 14b |
| TR1450\|c1_g1_i6 | 0.00E+000 | -4.34 |  |
| TR6477\|c0_g1_i2 | 8.45E-011 | -4.34 |  |
| TR14977\|c0_g1_i1 | 5.54E-010 | -4.33 | abc transporter b family member 2-like |
| TR24391\|c0_g2_i1 | 0.00E+000 | -4.32 |  |
| TR20122\|c1_g2_i10 | 0.00E+000 | -4.31 |  |
| TR1450\|c1_g1_i3 | 2.52E-007 | -4.31 |  |
| TR12817\|c0_g1_i1 | 0.00E+000 | -4.30 |  |
| TR12526\|c0_g1_i1 | 0.00E+000 | -4.30 |  |
| TR2513\|c0_g1_i1 | 2.76E-004 | -4.29 |  |
| TR21399\|c0_g1_i1 | 5.55E-006 | -4.29 |  |
| TR25217\|c0_g1_i1 | 0.00E+000 | -4.28 |  |
| TR15424\|c0_g1_i1 | 8.89E-004 | -4.26 |  |
| TR24121\|c0_g1_i1 | 0.00E+000 | -4.25 |  |
| TR21112\|c1_g1_i1 | 0.00E+000 | -4.24 |  |
| TR23503\|c0_g1_i1 | 0.00E+000 | -4.24 |  |
| TR2272\|c2_g4_i5 | 1.22E-003 | -4.23 |  |
| TR19076\|c0_g1_i1 | 4.00E-003 | -4.22 |  |
| TR23267\|c2_g1_i1 | 0.00E+000 | -4.22 |  |
| TR17507\|c0_g1_i2 | 2.20E-012 | -4.20 |  |
| TR9244\|c0_g1_i1 | 0.00E+000 | -4.19 |  |
| TR23267\|c2_g6_i2 | 0.00E+000 | -4.19 |  |
| TR12108\|c0_g1_i1 | 0.00E+000 | -4.16 | protein |
| TR18089\|c0_g1_i2 | 2.01E-005 | -4.13 | e3 ubiquitin-protein ligase ring1-like protein |
| TR16003\|c0_g1_i1 | 0.00E+000 | -4.12 |  |
| TR975\|c0_g1_i1 | 8.88E-016 | -4.12 |  |
| TR22827\|c0_g1_i1 | 1.06E-003 | -4.12 |  |
| TR15083\|c0_g1_i1 | 7.52E-007 | -4.11 |  |
| TR15158\|c1_g1_i1 | 0.00E+000 | -4.11 |  |
| TR10843\|c0_g1_i9 | 0.00E+000 | -4.11 | triacylglycerol lipase |
| TR11793\|c0_g1_i1 | 4.01E-013 | -4.10 |  |
| TR2059\|c0_g1_i1 | 0.00E+000 | -4.10 |  |
| TR15240\|c0_g1_i1 | 9.13E-013 | -4.06 |  |
| TR19710\|c0_g1_i3 | 0.00E+000 | -4.06 | hnh endonuclease family protein |
| TR7936\|c0_g1_i2 | 4.85E-004 | -4.05 |  |
| TR9239\|c0_g1_i1 | 0.00E+000 | -4.04 | luminal-binding protein |
| TR15829\|c0_g1_i1 | 2.48E-009 | -4.04 | zinc finger swim domain-containing protein 7 |
| TR25697\|c0_g1_i1 | 0.00E+000 | -4.04 |  |
| TR25339\|c0_g1_i3 | 7.25E-003 | -4.03 |  |
| TR2323\|c0_g1_i1 | 0.00E+000 | -4.03 |  |
| TR20388\|c0_g1_i8 | 3.92E-004 | -4.02 |  |
| TR6321\|c1_g1_i2 | 8.04E-013 | -4.01 | soluble nsf attachment protein |
| TR10843\|c0_g1_i6 | 0.00E+000 | -4.00 | triacylglycerol lipase |
| TR20035\|c6_g1_i5 | 5.69E-012 | -4.00 |  |
| TR8795\|c0_g1_i1 | 0.00E+000 | -4.00 | galactose oxidase |
| TR11312\|c6_g1_i4 | 1.81E-007 | -3.99 |  |
| TR16031\|c0_g1_i2 | 0.00E+000 | -3.99 | vacuolar transporter chaperone 4 |
| TR25114\|c0_g1_i2 | 6.53E-006 | -3.98 | u2 snrnp component ist3 |
| TR407\|c0_g1_i5 | 0.00E+000 | -3.97 | nep1-interacting 1 |
| TR5950\|c0_g1_i1 | 2.19E-011 | -3.97 |  |
| TR2196\|c0_g1_i1 | 0.00E+000 | -3.97 |  |
| TR7277\|c0_g1_i1 | 0.00E+000 | -3.97 | atp-dependent protease atpase subunit |
| TR14577\|c0_g1_i1 | 1.92E-005 | -3.96 |  |
| TR9219\|c0_g1_i1 | 0.00E+000 | -3.95 | neuroglobin |
| TR2141\|c0_g1_i2 | 3.06E-009 | -3.95 |  |
| TR13134\|c0_g1_i1 | 2.06E-003 | -3.95 |  |
| TR8961\|c0_g1_i2 | 0.00E+000 | -3.93 | homogentisate -dioxygenase |
| TR18104\|c0_g1_i1 | 0.00E+000 | -3.90 |  |
| TR1165\|c0_g1_i2 | 1.02E-008 | -3.89 | protein-tyrosine phosphatase |
| TR24558\|c0_g1_i1 | 2.67E-004 | -3.88 |  |
| TR8961\|c0_g1_i1 | 0.00E+000 | -3.88 | homogentisate -dioxygenase |
| TR23298\|c1_g1_i2 | 3.97E-005 | -3.87 | sulfatase- sulf- |
| TR9208\|c0_g2_i1 | 0.00E+000 | -3.86 | predicted protein |
| TR11333\|c1_g2_i2 | 8.33E-006 | -3.84 | protein |
| TR2268\|c4_g1_i7 | 0.00E+000 | -3.83 | transmembrane protein with metallophosphoesterase domain |
| TR9239\|c0_g2_i1 | 0.00E+000 | -3.82 | luminal-binding protein |
| TR912\|c0_g2_i1 | 1.08E-011 | -3.82 | omega-6 fatty acid desaturase (delta-12 desaturase) |
| TR24293\|c0_g1_i1 | 0.00E+000 | -3.82 |  |
| TR20122\|c1_g2_i7 | 0.00E+000 | -3.81 | protein |
| TR12592\|c0_g1_i1 | 3.60E-004 | -3.81 |  |
| TR16025\|c0_g1_i8 | 0.00E+000 | -3.80 |  |
| TR2494\|c0_g1_i1 | 2.22E-016 | -3.80 |  |
| TR407\|c0_g1_i1 | 0.00E+000 | -3.80 | nep1-interacting 1 |
| TR11749\|c0_g1_i1 | 2.80E-004 | -3.79 |  |
| TR16058\|c2_g1_i2 | 0.00E+000 | -3.78 | probable lrr receptor-like serine threonine-protein kinase at1g34110-like |
| TR20064\|c2_g4_i8 | 5.47E-003 | -3.77 | protein |
| TR16938\|c0_g1_i2 | 2.24E-004 | -3.76 |  |
| TR14393\|c1_g1_i1 | 1.43E-011 | -3.76 |  |
| TR19379\|c4_g1_i4 | 4.96E-003 | -3.75 | protein |
| TR6929\|c0_g1_i1 | 0.00E+000 | -3.74 | glycosyl group 1 family protein |
| TR2474\|c0_g1_i1 | 0.00E+000 | -3.74 |  |
| TR15267\|c0_g1_i2 | 0.00E+000 | -3.73 |  |
| TR20085\|c0_g4_i1 | 2.22E-016 | -3.73 |  |
| TR2171\|c0_g1_i5 | 1.05E-005 | -3.73 |  |
| TR24552\|c0_g1_i1 | 0.00E+000 | -3.72 | isochorismatase family protein family |
| TR8269\|c0_g2_i2 | 0.00E+000 | -3.72 |  |
| TR6647\|c0_g1_i1 | 0.00E+000 | -3.70 | diacylglycerol acyltransferase type 2a |
| TR2883\|c0_g1_i1 | 5.05E-009 | -3.68 | predicted protein |
| TR1941\|c0_g1_i1 | 2.22E-016 | -3.68 |  |
| TR12729\|c0_g2_i1 | 3.16E-007 | -3.67 |  |
| TR4199\|c1_g1_i3 | 2.65E-004 | -3.65 |  |
| TR21611\|c1_g1_i1 | 0.00E+000 | -3.65 | predicted protein |
| TR14419\|c0_g1_i1 | 0.00E+000 | -3.64 |  |
| TR16083\|c0_g1_i1 | 3.14E-003 | -3.63 | predicted protein |
| TR6581\|c0_g1_i1 | 2.72E-005 | -3.63 |  |
| TR2268\|c4_g5_i1 | 0.00E+000 | -3.63 |  |
| TR20535\|c0_g4_i1 | 0.00E+000 | -3.62 |  |
| TR551\|c0_g1_i2 | 4.00E-009 | -3.61 |  |
| TR407\|c0_g1_i3 | 2.47E-003 | -3.60 | nep1-interacting 1 |
| TR16643\|c0_g1_i1 | 0.00E+000 | -3.60 | dna damage-inducible protein 1 |
| TR891\|c0_g1_i1 | 5.85E-012 | -3.60 |  |
| TR6925\|c0_g1_i2 | 0.00E+000 | -3.60 | duf1244 molybdenum cofactor synthesis fusion protein |
| TR1775\|c0_g1_i1 | 1.42E-003 | -3.59 |  |
| TR18686\|c0_g1_i1 | 4.92E-005 | -3.59 | serine threonine-protein kinase ctr1 |
| TR23285\|c1_g6_i1 | 4.25E-004 | -3.58 | membrane protein tms1d |
| TR3778\|c0_g1_i5 | 1.36E-003 | -3.57 |  |
| TR6469\|c0_g1_i1 | 3.33E-016 | -3.56 | lipase family protein |
| TR18157\|c0_g1_i1 | 4.20E-011 | -3.56 |  |
| TR23576\|c1_g1_i2 | 0.00E+000 | -3.56 |  |
| TR7312\|c1_g1_i4 | 0.00E+000 | -3.55 | lactosylceramide 4-alpha-galactosyltransferase |
| TR8579\|c0_g1_i5 | 1.77E-003 | -3.55 | predicted protein |
| TR25388\|c0_g1_i1 | 0.00E+000 | -3.54 |  |
| TR25140\|c0_g1_i1 | 0.00E+000 | -3.53 | clavaminate synthase-like protein at3g21360 |
| TR6273\|c0_g1_i2 | 1.30E-005 | -3.53 |  |
| TR13978\|c0_g1_i1 | 0.00E+000 | -3.53 |  |
| TR20049\|c1_g1_i2 | 1.60E-008 | -3.52 |  |
| TR21107\|c1_g2_i1 | 2.55E-007 | -3.52 | long-chain-fatty-acid-- |
| TR13681\|c0_g1_i1 | 3.72E-005 | -3.52 |  |
| TR15312\|c0_g2_i1 | 2.28E-003 | -3.52 |  |
| TR12616\|c0_g1_i2 | 6.77E-004 | -3.51 | cyclin-dependent kinase c-1-like |
| TR11276\|c6_g1_i1 | 0.00E+000 | -3.51 |  |
| TR8287\|c0_g1_i3 | 0.00E+000 | -3.51 |  |
| TR6330\|c0_g1_i2 | 0.00E+000 | -3.51 |  |
| TR11332\|c0_g1_i8 | 1.81E-003 | -3.50 | protein |
| TR25001\|c0_g1_i2 | 0.00E+000 | -3.48 |  |
| TR56\|c0_g1_i3 | 0.00E+000 | -3.48 |  |
| TR2268\|c4_g1_i3 | 0.00E+000 | -3.48 | transmembrane protein with metallophosphoesterase domain |
| TR14415\|c0_g1_i1 | 0.00E+000 | -3.47 | protein |
| TR22098\|c0_g1_i1 | 4.44E-016 | -3.47 |  |
| TR25290\|c0_g1_i1 | 0.00E+000 | -3.47 |  |
| TR11356\|c2_g1_i1 | 5.75E-005 | -3.46 |  |
| TR21112\|c1_g3_i1 | 7.85E-005 | -3.46 | gag-pol polyprotein |
| TR6655\|c0_g1_i5 | 3.77E-013 | -3.44 | protein |
| TR24510\|c0_g1_i1 | 0.00E+000 | -3.44 |  |
| TR2171\|c0_g1_i1 | 1.11E-015 | -3.44 |  |
| TR16945\|c0_g1_i1 | 4.99E-010 | -3.44 |  |
| TR15139\|c0_g1_i2 | 1.47E-003 | -3.44 | protein |
| TR1297\|c0_g3_i1 | 9.35E-009 | -3.43 |  |
| TR12108\|c0_g1_i5 | 0.00E+000 | -3.41 | protein |
| TR6585\|c0_g6_i5 | 6.82E-005 | -3.41 |  |
| TR14812\|c1_g2_i2 | 0.00E+000 | -3.39 | 60s ribosomal protein l3 |
| TR18883\|c0_g1_i2 | 0.00E+000 | -3.39 | methylcrotonoyl- carboxylase beta mitochondrial |
| TR16845\|c0_g1_i2 | 5.85E-003 | -3.39 |  |
| TR7650\|c0_g1_i1 | 5.11E-004 | -3.38 |  |
| TR19179\|c0_g1_i1 | 7.06E-009 | -3.37 |  |
| TR10366\|c0_g2_i1 | 0.00E+000 | -3.36 |  |
| TR3743\|c4_g2_i6 | 0.00E+000 | -3.36 |  |
| TR15860\|c1_g1_i1 | 1.27E-009 | -3.36 |  |
| TR15139\|c0_g1_i4 | 5.20E-007 | -3.35 | protein |
| TR8913\|c0_g1_i1 | 0.00E+000 | -3.34 |  |
| TR24347\|c0_g2_i2 | 6.15E-003 | -3.33 |  |
| TR19423\|c0_g2_i1 | 0.00E+000 | -3.33 |  |
| TR15621\|c1_g1_i1 | 0.00E+000 | -3.32 |  |
| TR14773\|c0_g1_i1 | 0.00E+000 | -3.32 |  |
| TR6616\|c1_g1_i1 | 0.00E+000 | -3.32 |  |
| TR20242\|c0_g1_i1 | 0.00E+000 | -3.32 |  |
| TR506\|c0_g2_i1 | 0.00E+000 | -3.32 | 4-hydroxyphenylpyruvate dioxygenase |
| TR11589\|c0_g1_i1 | 0.00E+000 | -3.30 |  |
| TR16114\|c0_g1_i1 | 0.00E+000 | -3.30 |  |
| TR1422\|c0_g1_i4 | 0.00E+000 | -3.30 | probable purple acid phosphatase 20 |
| TR11569\|c0_g1_i1 | 3.34E-005 | -3.30 |  |
| TR962\|c0_g1_i1 | 1.75E-004 | -3.30 | long chain acyl- synthetase |
| TR4586\|c0_g1_i1 | 0.00E+000 | -3.29 |  |
| TR724\|c0_g2_i1 | 4.65E-003 | -3.29 |  |
| TR21132\|c0_g1_i1 | 6.63E-010 | -3.29 |  |
| TR20193\|c0_g1_i3 | 3.39E-008 | -3.29 |  |
| TR22405\|c1_g1_i1 | 0.00E+000 | -3.29 | mitochondrial rho 1 |
| TR24655\|c0_g1_i1 | 0.00E+000 | -3.28 |  |
| TR6457\|c0_g1_i1 | 0.00E+000 | -3.28 |  |
| TR11297\|c0_g1_i1 | 0.00E+000 | -3.28 |  |
| TR3498\|c0_g1_i2 | 0.00E+000 | -3.28 |  |
| TR22857\|c0_g1_i2 | 2.80E-011 | -3.26 | kinesin like protein |
| TR7171\|c1_g6_i1 | 0.00E+000 | -3.26 | protein |
| TR4392\|c0_g1_i1 | 0.00E+000 | -3.26 | dnaj homolog subfamily b member 9 |
| TR16718\|c0_g1_i1 | 0.00E+000 | -3.26 |  |
| TR12958\|c0_g1_i1 | 0.00E+000 | -3.26 | mitochondrial inner membrane metallopeptidase oma1 |
| TR13377\|c0_g1_i1 | 0.00E+000 | -3.26 | protein |
| TR5844\|c0_g1_i1 | 0.00E+000 | -3.26 | serine threonine protein kinase |
| TR2323\|c0_g1_i4 | 0.00E+000 | -3.25 |  |
| TR4798\|c0_g3_i2 | 3.92E-003 | -3.25 |  |
| TR15501\|c0_g1_i1 | 4.93E-005 | -3.24 |  |
| TR22386\|c0_g1_i2 | 3.91E-010 | -3.24 | spfh domain band 7 family protein |
| TR24604\|c0_g1_i2 | 0.00E+000 | -3.24 |  |
| TR2129\|c2_g1_i1 | 1.89E-015 | -3.24 |  |
| TR25332\|c0_g1_i1 | 0.00E+000 | -3.24 | predicted protein |
| TR13160\|c0_g1_i1 | 0.00E+000 | -3.24 |  |
| TR15979\|c0_g1_i1 | 0.00E+000 | -3.23 |  |
| TR6610\|c0_g1_i1 | 0.00E+000 | -3.23 |  |
| TR17236\|c0_g3_i1 | 0.00E+000 | -3.23 |  |
| TR24604\|c0_g1_i1 | 0.00E+000 | -3.23 |  |
| TR24436\|c0_g1_i1 | 0.00E+000 | -3.22 | o-acetylhomoserine -lyase |
| TR8421\|c0_g1_i2 | 4.72E-005 | -3.22 |  |
| TR23267\|c2_g4_i11 | 0.00E+000 | -3.22 | heat shock protein |
| TR23605\|c0_g1_i1 | 6.54E-003 | -3.20 |  |
| TR2129\|c0_g1_i2 | 0.00E+000 | -3.19 |  |
| TR16250\|c0_g1_i1 | 0.00E+000 | -3.19 |  |
| TR4195\|c1_g1_i2 | 0.00E+000 | -3.19 |  |
| TR24666\|c0_g1_i2 | 2.02E-009 | -3.19 |  |
| TR1434\|c2_g1_i1 | 1.09E-004 | -3.18 |  |
| TR18169\|c0_g1_i1 | 0.00E+000 | -3.18 | 3-ketoacyl- partial |
| TR23217\|c0_g1_i3 | 3.91E-004 | -3.17 | pt repeat family protein |
| TR20171\|c1_g1_i1 | 0.00E+000 | -3.16 |  |
| TR2315\|c9_g3_i2 | 1.67E-011 | -3.16 |  |
| TR3738\|c3_g2_i1 | 0.00E+000 | -3.16 |  |
| TR15123\|c0_g1_i1 | 0.00E+000 | -3.16 | calcium-dependent protein kinase |
| TR23001\|c0_g1_i1 | 0.00E+000 | -3.15 |  |
| TR8018\|c2_g1_i2 | 8.91E-004 | -3.15 |  |
| TR23246\|c1_g1_i1 | 2.05E-004 | -3.15 |  |
| TR16005\|c1_g5_i6 | 2.07E-003 | -3.15 |  |
| TR12589\|c0_g1_i2 | 1.82E-005 | -3.13 | isoamyl acetate-hydrolyzing esterase 1 homolog |
| TR11748\|c0_g1_i1 | 4.71E-010 | -3.13 | v-type proton atpase proteolipid subunit |
| TR17483\|c0_g1_i1 | 2.30E-006 | -3.12 |  |
| TR3731\|c4_g1_i1 | 0.00E+000 | -3.12 | alpha beta hydrolase fold protein |
| TR8430\|c0_g1_i1 | 0.00E+000 | -3.12 |  |
| TR20130\|c1_g1_i4 | 6.70E-005 | -3.11 | tkl dicty4 protein kinase |
| TR10733\|c0_g2_i1 | 1.87E-010 | -3.11 |  |
| TR1121\|c0_g1_i1 | 0.00E+000 | -3.10 |  |
| TR1567\|c0_g1_i1 | 0.00E+000 | -3.09 | cinnamoyl- reductase |
| TR24340\|c0_g1_i2 | 3.51E-004 | -3.09 | protein chloroplastic-like |
| TR6333\|c0_g2_i2 | 2.19E-004 | -3.09 |  |
| TR18\|c0_g1_i1 | 0.00E+000 | -3.08 |  |
| TR19423\|c0_g1_i1 | 0.00E+000 | -3.08 |  |
| TR6456\|c0_g1_i1 | 2.71E-004 | -3.07 | ef-hand calcium-binding site |
| TR20610\|c0_g1_i2 | 8.18E-004 | -3.06 |  |
| TR6698\|c0_g1_i1 | 0.00E+000 | -3.06 | chloroplast fructose- -bisphosphatase |
| TR3737\|c13_g1_i7 | 9.30E-006 | -3.06 |  |
| TR4821\|c0_g1_i1 | 0.00E+000 | -3.06 | hypothetical protein BRAFLDRAFT_75731 |
| TR7519\|c0_g9_i3 | 3.91E-006 | -3.05 |  |
| TR7878\|c0_g1_i2 | 0.00E+000 | -3.05 |  |
| TR20986\|c0_g1_i1 | 0.00E+000 | -3.05 |  |
| TR3055\|c3_g1_i3 | 2.11E-015 | -3.05 |  |
| TR19725\|c0_g1_i2 | 6.52E-010 | -3.05 |  |
| TR3123\|c2_g2_i1 | 0.00E+000 | -3.05 |  |
| TR23305\|c0_g2_i15 | 2.22E-016 | -3.04 |  |
| TR21107\|c1_g4_i1 | 3.81E-003 | -3.04 |  |
| TR18354\|c0_g1_i1 | 0.00E+000 | -3.03 | multi antimicrobial extrusion family protein |
| TR13646\|c0_g1_i1 | 5.96E-005 | -3.03 | glycoprotein endopeptidase kae1 |
| TR10475\|c1_g1_i1 | 8.24E-006 | -3.03 |  |
| TR10915\|c2_g1_i3 | 4.91E-008 | -3.03 |  |
| TR11303\|c0_g1_i7 | 8.11E-005 | -3.02 | protein |
| TR20118\|c0_g1_i1 | 6.09E-008 | -3.01 |  |
| TR22977\|c0_g1_i1 | 0.00E+000 | -3.01 | protein |
| TR19710\|c0_g1_i8 | 0.00E+000 | -3.01 | hnh endonuclease family protein |
| TR19454\|c0_g2_i1 | 9.33E-005 | -3.01 |  |
| TR7144\|c5_g1_i5 | 4.86E-003 | -3.00 |  |
| TR15554\|c0_g1_i1 | 0.00E+000 | -2.99 |  |
| TR24269\|c0_g1_i1 | 0.00E+000 | -2.99 |  |
| TR7478\|c2_g2_i6 | 0.00E+000 | -2.99 | protein |
| TR22115\|c0_g2_i1 | 0.00E+000 | -2.98 |  |
| TR19780\|c0_g1_i2 | 4.42E-007 | -2.98 | glutathione s- |
| TR8292\|c4_g2_i4 | 5.55E-016 | -2.98 |  |
| TR1409\|c0_g1_i2 | 2.70E-009 | -2.97 |  |
| TR22810\|c0_g1_i2 | 0.00E+000 | -2.97 |  |
| TR2272\|c2_g4_i1 | 8.76E-008 | -2.97 |  |
| TR2129\|c0_g1_i1 | 0.00E+000 | -2.97 |  |
| TR7171\|c0_g1_i1 | 0.00E+000 | -2.97 | atp-binding cassette |
| TR1434\|c7_g2_i2 | 6.31E-008 | -2.97 |  |
| TR1597\|c0_g1_i2 | 8.50E-004 | -2.96 |  |
| TR10078\|c0_g1_i1 | 2.02E-007 | -2.96 | protein yipf1 homolog |
| TR21876\|c1_g1_i1 | 0.00E+000 | -2.96 | dna j-like protein 2 |
| TR3437\|c0_g1_i1 | 0.00E+000 | -2.95 | protein |
| TR19299\|c0_g1_i2 | 7.81E-004 | -2.94 |  |
| TR18883\|c0_g1_i5 | 7.35E-006 | -2.93 | methylcrotonoyl- carboxylase beta mitochondrial |
| TR7197\|c0_g1_i1 | 0.00E+000 | -2.93 |  |
| TR7478\|c2_g2_i9 | 0.00E+000 | -2.93 |  |
| TR25290\|c0_g1_i2 | 0.00E+000 | -2.92 |  |
| TR16333\|c1_g1_i3 | 8.03E-013 | -2.92 | protein |
| TR25902\|c0_g1_i1 | 0.00E+000 | -2.91 | isocitrate lyase |
| TR10508\|c1_g1_i1 | 4.40E-006 | -2.91 |  |
| TR16106\|c1_g3_i1 | 0.00E+000 | -2.91 | protein |
| TR16005\|c1_g5_i1 | 0.00E+000 | -2.90 |  |
| TR6282\|c0_g1_i1 | 0.00E+000 | -2.90 | protein |
| TR10402\|c0_g1_i6 | 0.00E+000 | -2.90 | short chain dehydrogenase reductase family protein |
| TR20089\|c4_g1_i1 | 5.55E-015 | -2.89 |  |
| TR11314\|c0_g2_i1 | 3.27E-012 | -2.89 |  |
| TR8725\|c0_g1_i1 | 0.00E+000 | -2.89 | protein |
| TR2029\|c0_g1_i1 | 2.48E-005 | -2.89 | imp-specific 5 -nucleotidase 1 |
| TR20120\|c2_g1_i2 | 0.00E+000 | -2.89 | acetyl- synthetase-like protein |
| TR15158\|c0_g1_i1 | 0.00E+000 | -2.89 | gtp binding protein |
| TR24304\|c0_g1_i1 | 9.83E-008 | -2.89 |  |
| TR18559\|c0_g1_i3 | 1.48E-005 | -2.89 |  |
| TR21106\|c3_g2_i4 | 0.00E+000 | -2.88 | predicted protein |
| TR7174\|c0_g2_i1 | 0.00E+000 | -2.88 |  |
| TR10044\|c0_g2_i2 | 0.00E+000 | -2.88 |  |
| TR24794\|c0_g1_i1 | 0.00E+000 | -2.88 |  |
| TR18125\|c0_g1_i2 | 2.87E-004 | -2.88 |  |
| TR25179\|c0_g1_i1 | 0.00E+000 | -2.88 |  |
| TR11461\|c0_g1_i1 | 3.72E-014 | -2.87 | ectonucleoside triphosphate diphosphohydrolase 1 isoform x2 |
| TR22296\|c0_g1_i1 | 0.00E+000 | -2.87 | mfs general substrate transporter |
| TR6724\|c0_g1_i2 | 2.48E-010 | -2.86 |  |
| TR3725\|c0_g1_i4 | 0.00E+000 | -2.86 |  |
| TR14716\|c0_g1_i1 | 0.00E+000 | -2.86 |  |
| TR3724\|c2_g6_i6 | 0.00E+000 | -2.86 | abc abc-g wbc-type |
| TR18105\|c0_g1_i1 | 9.12E-003 | -2.86 |  |
| TR23890\|c0_g2_i1 | 0.00E+000 | -2.86 |  |
| TR6444\|c0_g1_i2 | 0.00E+000 | -2.85 |  |
| TR24182\|c0_g1_i1 | 1.90E-003 | -2.85 |  |
| TR6221\|c0_g1_i1 | 0.00E+000 | -2.85 |  |
| TR15599\|c0_g1_i3 | 6.14E-005 | -2.85 | nitroreductase family protein |
| TR15120\|c1_g1_i1 | 7.65E-005 | -2.84 |  |
| TR20412\|c0_g1_i1 | 0.00E+000 | -2.84 | probable galactinol--sucrose galactosyltransferase 2 |
| TR8013\|c0_g1_i1 | 3.54E-011 | -2.84 |  |
| TR7158\|c0_g3_i2 | 0.00E+000 | -2.84 |  |
| TR4798\|c0_g3_i1 | 0.00E+000 | -2.83 |  |
| TR15592\|c0_g1_i1 | 0.00E+000 | -2.83 |  |
| TR2171\|c1_g1_i1 | 0.00E+000 | -2.83 |  |
| TR16750\|c0_g1_i1 | 0.00E+000 | -2.82 |  |
| TR6453\|c0_g1_i1 | 1.44E-009 | -2.82 |  |
| TR12119\|c0_g1_i1 | 0.00E+000 | -2.82 | cax-interacting protein 4 |
| TR21080\|c1_g1_i1 | 8.27E-004 | -2.82 |  |
| TR11282\|c0_g1_i1 | 2.78E-005 | -2.82 |  |
| TR25623\|c0_g1_i1 | 0.00E+000 | -2.81 |  |
| TR9683\|c0_g2_i1 | 1.02E-012 | -2.81 |  |
| TR1474\|c0_g1_i1 | 2.22E-016 | -2.81 | transketolase |
| TR8154\|c0_g1_i1 | 0.00E+000 | -2.81 | dna helicase |
| TR2268\|c4_g4_i1 | 0.00E+000 | -2.81 |  |
| TR10542\|c0_g3_i2 | 8.06E-013 | -2.81 | protein |
| TR13563\|c0_g1_i1 | 4.25E-006 | -2.81 |  |
| TR23267\|c2_g2_i1 | 0.00E+000 | -2.80 |  |
| TR9024\|c0_g1_i1 | 0.00E+000 | -2.80 | tyrosine aminotransferase |
| TR19297\|c0_g1_i1 | 3.48E-008 | -2.80 |  |
| TR8306\|c0_g1_i2 | 0.00E+000 | -2.80 | lipase class 3 family protein |
| TR23267\|c2_g6_i1 | 0.00E+000 | -2.80 |  |
| TR2086\|c0_g1_i1 | 0.00E+000 | -2.80 |  |
| TR20321\|c0_g1_i1 | 0.00E+000 | -2.79 |  |
| TR1171\|c0_g1_i1 | 4.25E-004 | -2.79 |  |
| TR8311\|c1_g1_i11 | 8.88E-016 | -2.79 |  |
| TR21076\|c2_g1_i13 | 5.29E-009 | -2.79 | protein |
| TR21622\|c0_g4_i1 | 1.18E-003 | -2.79 |  |
| TR10060\|c0_g1_i1 | 3.31E-012 | -2.79 | ring finger-like protein |
| TR2125\|c0_g1_i1 | 0.00E+000 | -2.79 |  |
| TR11285\|c0_g2_i2 | 5.54E-004 | -2.78 |  |
| TR4872\|c0_g1_i1 | 4.87E-008 | -2.78 |  |
| TR11374\|c0_g1_i3 | 9.07E-003 | -2.78 | protein |
| TR24139\|c0_g1_i2 | 2.23E-003 | -2.78 |  |
| TR7949\|c0_g2_i2 | 6.21E-005 | -2.77 |  |
| TR9714\|c1_g1_i1 | 0.00E+000 | -2.77 | vacuolar h |
| TR21106\|c3_g2_i3 | 0.00E+000 | -2.77 | protein |
| TR21113\|c5_g2_i1 | 1.72E-006 | -2.77 |  |
| TR18527\|c0_g1_i1 | 2.06E-004 | -2.76 |  |
| TR6616\|c2_g1_i2 | 3.36E-010 | -2.76 |  |
| TR18578\|c1_g1_i5 | 1.84E-011 | -2.76 |  |
| TR21362\|c0_g1_i1 | 0.00E+000 | -2.76 |  |
| TR3720\|c1_g4_i1 | 1.49E-003 | -2.75 |  |
| TR13682\|c0_g1_i1 | 7.77E-016 | -2.75 |  |
| TR3090\|c1_g1_i1 | 0.00E+000 | -2.75 |  |
| TR1927\|c0_g1_i1 | 3.59E-004 | -2.74 |  |
| TR8715\|c0_g1_i1 | 0.00E+000 | -2.74 |  |
| TR25292\|c0_g1_i1 | 0.00E+000 | -2.74 | domain-containing protein |
| TR10862\|c0_g1_i2 | 1.22E-004 | -2.74 | carbonyl reductase |
| TR6715\|c0_g1_i2 | 5.15E-006 | -2.74 |  |
| TR17280\|c0_g1_i2 | 0.00E+000 | -2.73 |  |
| TR5218\|c0_g1_i1 | 0.00E+000 | -2.73 | acetyl- biotin carboxylase subunit |
| TR21876\|c0_g1_i3 | 0.00E+000 | -2.73 | -domain-containing protein |
| TR20351\|c0_g1_i2 | 1.28E-005 | -2.73 | bile acid:na+ symporter family |
| TR9697\|c0_g1_i1 | 0.00E+000 | -2.73 |  |
| TR9454\|c0_g1_i1 | 0.00E+000 | -2.72 |  |
| TR10402\|c0_g1_i3 | 0.00E+000 | -2.72 | short chain dehydrogenase reductase family protein |
| TR23728\|c0_g1_i1 | 0.00E+000 | -2.72 |  |
| TR9362\|c0_g1_i2 | 0.00E+000 | -2.72 | -trans-enoyl- mitochondrial |
| TR10525\|c1_g3_i5 | 5.05E-005 | -2.71 |  |
| TR15245\|c0_g1_i1 | 8.29E-009 | -2.71 | synaptobrevin like ykt6 |
| TR8145\|c0_g1_i1 | 0.00E+000 | -2.71 | kraken |
| TR4657\|c0_g1_i1 | 3.10E-013 | -2.71 |  |
| TR19421\|c6_g2_i1 | 6.82E-009 | -2.71 |  |
| TR23886\|c0_g1_i1 | 2.07E-013 | -2.71 |  |
| TR22333\|c1_g1_i1 | 0.00E+000 | -2.71 | vacuolar h |
| TR10843\|c0_g1_i4 | 1.51E-005 | -2.70 | triacylglycerol lipase |
| TR5470\|c0_g1_i1 | 0.00E+000 | -2.69 | rna polymerase sigma factor |
| TR22405\|c1_g1_i3 | 4.82E-004 | -2.69 |  |
| TR11113\|c0_g1_i2 | 3.50E-006 | -2.69 |  |
| TR23930\|c0_g1_i1 | 2.84E-012 | -2.69 |  |
| TR2088\|c0_g1_i5 | 5.05E-004 | -2.68 | cyclin-p3-1-like isoform x2 |
| TR20047\|c0_g3_i4 | 1.28E-007 | -2.68 |  |
| TR13207\|c0_g1_i1 | 0.00E+000 | -2.68 |  |
| TR7144\|c8_g5_i1 | 0.00E+000 | -2.68 |  |
| TR23217\|c0_g1_i2 | 4.89E-006 | -2.68 | pt repeat family protein |
| TR24108\|c0_g1_i2 | 0.00E+000 | -2.68 | jumonji arid domain-containing protein 1a |
| TR16112\|c1_g1_i4 | 1.54E-003 | -2.67 | protein |
| TR8158\|c0_g2_i3 | 3.67E-005 | -2.67 |  |
| TR23576\|c0_g1_i1 | 0.00E+000 | -2.67 |  |
| TR14285\|c0_g1_i1 | 0.00E+000 | -2.67 | protein |
| TR3611\|c0_g5_i1 | 7.55E-013 | -2.67 |  |
| TR8318\|c1_g1_i2 | 0.00E+000 | -2.67 |  |
| TR11878\|c0_g1_i2 | 1.07E-003 | -2.67 |  |
| TR21122\|c0_g1_i1 | 1.39E-003 | -2.66 |  |
| TR24227\|c0_g1_i1 | 5.65E-004 | -2.66 |  |
| TR19684\|c0_g1_i1 | 4.19E-011 | -2.66 |  |
| TR10035\|c0_g1_i1 | 0.00E+000 | -2.66 | acetyl- biotin carboxylase subunit |
| TR18918\|c0_g1_i1 | 0.00E+000 | -2.66 | bromodomain containing protein |
| TR19412\|c1_g1_i4 | 1.23E-009 | -2.65 | zinc finger dhhc domain containing related |
| TR20116\|c0_g1_i7 | 4.79E-003 | -2.65 |  |
| TR17759\|c0_g1_i1 | 5.66E-009 | -2.65 | protein |
| TR25926\|c0_g1_i2 | 0.00E+000 | -2.64 | succinate fumarate mitochondrial transporter |
| TR2323\|c0_g1_i2 | 0.00E+000 | -2.64 |  |
| TR19495\|c0_g1_i2 | 0.00E+000 | -2.64 | atp dependent dna ligase |
| TR7407\|c0_g1_i1 | 0.00E+000 | -2.64 | protein |
| TR13517\|c0_g1_i1 | 0.00E+000 | -2.64 | quinone-oxidoreductase chloroplastic |
| TR4319\|c1_g1_i1 | 5.72E-006 | -2.64 | pyruvate carboxylase |
| TR14289\|c0_g1_i1 | 2.41E-011 | -2.64 | unnamed protein product |
| TR12411\|c0_g1_i2 | 4.41E-007 | -2.64 | atp-dependent clp protease proteolytic subunit |
| TR21076\|c1_g4_i3 | 3.84E-009 | -2.64 | protein |
| TR21150\|c0_g1_i2 | 0.00E+000 | -2.64 | abc transporter |
| TR7478\|c2_g1_i1 | 0.00E+000 | -2.63 |  |
| TR11360\|c3_g2_i1 | 0.00E+000 | -2.63 |  |
| TR13648\|c0_g1_i2 | 3.35E-007 | -2.63 | kynurenine 3-monooxygenase |
| TR3664\|c0_g1_i1 | 0.00E+000 | -2.63 |  |
| TR3123\|c2_g2_i3 | 1.82E-010 | -2.63 |  |
| TR24277\|c0_g2_i1 | 0.00E+000 | -2.62 |  |
| TR12733\|c0_g1_i1 | 0.00E+000 | -2.62 | protein |
| TR25354\|c0_g1_i2 | 0.00E+000 | -2.62 |  |
| TR15096\|c0_g1_i1 | 1.81E-005 | -2.62 | calcium-dependent protein |
| TR6577\|c1_g1_i2 | 2.65E-007 | -2.62 |  |
| TR20059\|c1_g2_i5 | 0.00E+000 | -2.61 | ring-h2 finger protein atl65 |
| TR13170\|c0_g1_i1 | 8.10E-003 | -2.61 |  |
| TR25976\|c0_g2_i1 | 0.00E+000 | -2.61 |  |
| TR11938\|c0_g1_i1 | 3.72E-007 | -2.61 |  |
| TR11390\|c0_g1_i4 | 8.64E-006 | -2.61 |  |
| TR2323\|c0_g1_i5 | 0.00E+000 | -2.61 |  |
| TR6984\|c0_g1_i1 | 2.71E-003 | -2.61 |  |
| TR451\|c0_g1_i1 | 0.00E+000 | -2.61 | antibiotic transport system atp-binding partial |
| TR11310\|c1_g1_i3 | 5.28E-011 | -2.60 |  |
| TR7247\|c0_g1_i2 | 0.00E+000 | -2.60 |  |
| TR10425\|c0_g1_i1 | 7.88E-015 | -2.60 |  |
| TR24668\|c0_g1_i1 | 3.33E-016 | -2.60 |  |
| TR1297\|c0_g2_i3 | 0.00E+000 | -2.60 | vacuolar iron family transporter |
| TR16313\|c0_g1_i1 | 0.00E+000 | -2.60 |  |
| TR5221\|c1_g1_i3 | 2.89E-003 | -2.60 |  |
| TR1133\|c0_g1_i1 | 1.46E-005 | -2.60 | nicotinate nucleotide adenylyltransferase |
| TR15120\|c1_g1_i2 | 0.00E+000 | -2.60 |  |
| TR21653\|c0_g1_i1 | 6.63E-005 | -2.60 |  |
| TR10060\|c0_g1_i3 | 0.00E+000 | -2.59 | ring finger-like protein |
| TR955\|c0_g1_i1 | 0.00E+000 | -2.59 |  |
| TR15504\|c0_g3_i1 | 0.00E+000 | -2.59 | cytochrome p450 4f12 |
| TR6649\|c1_g1_i4 | 8.59E-008 | -2.58 |  |
| TR10863\|c0_g1_i3 | 1.69E-003 | -2.58 | glutamyl-trna amidotransferase subunit |
| TR10454\|c4_g2_i3 | 0.00E+000 | -2.58 | protein |
| TR22297\|c0_g1_i1 | 2.46E-005 | -2.57 |  |
| TR19335\|c0_g1_i1 | 1.40E-003 | -2.57 |  |
| TR5543\|c3_g1_i2 | 4.94E-003 | -2.57 |  |
| TR3728\|c2_g1_i1 | 0.00E+000 | -2.57 | dde family endonuclease |
| TR20388\|c0_g1_i3 | 0.00E+000 | -2.56 |  |
| TR5322\|c0_g1_i1 | 6.39E-013 | -2.56 | protein |
| TR25195\|c0_g1_i1 | 1.35E-004 | -2.56 |  |
| TR3731\|c7_g1_i1 | 1.14E-007 | -2.56 |  |
| TR11406\|c0_g1_i2 | 3.30E-004 | -2.56 | phox domain-containing |
| TR10542\|c0_g2_i2 | 0.00E+000 | -2.55 | atp-binding cassette |
| TR10862\|c0_g1_i1 | 2.39E-012 | -2.55 | carbonyl reductase |
| TR962\|c0_g1_i4 | 7.88E-015 | -2.55 | long chain acyl- synthetase |
| TR1409\|c1_g1_i2 | 8.74E-003 | -2.55 |  |
| TR12943\|c0_g1_i1 | 0.00E+000 | -2.55 |  |
| TR8326\|c0_g1_i2 | 0.00E+000 | -2.55 |  |
| TR15081\|c0_g1_i2 | 1.34E-005 | -2.55 | patatin-like phospholipase domain-containing protein |
| TR7478\|c2_g2_i2 | 0.00E+000 | -2.55 | protein |
| TR3707\|c0_g1_i7 | 9.76E-005 | -2.54 |  |
| TR24980\|c1_g1_i1 | 1.45E-005 | -2.54 |  |
| TR25346\|c0_g1_i1 | 4.85E-010 | -2.54 | atp-dependent protease la |
| TR9803\|c0_g2_i1 | 0.00E+000 | -2.54 | carnitine o-acetyltransferase |
| TR10560\|c6_g4_i12 | 2.56E-004 | -2.52 |  |
| TR10843\|c0_g1_i2 | 9.79E-009 | -2.52 | triacylglycerol lipase |
| TR12536\|c0_g1_i1 | 7.68E-011 | -2.52 | villin 1- |
| TR2196\|c0_g1_i2 | 0.00E+000 | -2.52 |  |
| TR16034\|c1_g1_i10 | 9.90E-003 | -2.51 |  |
| TR10536\|c0_g1_i11 | 0.00E+000 | -2.51 |  |
| TR11259\|c0_g1_i1 | 1.08E-003 | -2.51 | reverse transcriptase homolog |
| TR25161\|c0_g1_i2 | 0.00E+000 | -2.51 | rna exonuclease 4 |
| TR23223\|c4_g2_i4 | 0.00E+000 | -2.50 |  |
| TR10682\|c0_g1_i1 | 8.38E-006 | -2.50 |  |
| TR9258\|c0_g1_i2 | 0.00E+000 | -2.50 |  |
| TR23303\|c4_g2_i2 | 1.57E-003 | -2.50 |  |
| TR1693\|c0_g1_i1 | 0.00E+000 | -2.50 | predicted protein |
| TR4411\|c0_g1_i1 | 0.00E+000 | -2.50 | duf775 domain protein |
| TR22408\|c0_g1_i1 | 1.86E-007 | -2.50 | efflux partial |
| TR13698\|c0_g1_i2 | 0.00E+000 | -2.50 | transmembrane o-methyltransferase |
| TR390\|c0_g1_i1 | 0.00E+000 | -2.49 |  |
| TR10850\|c2_g1_i7 | 2.16E-014 | -2.49 |  |
| TR219\|c0_g1_i2 | 9.41E-010 | -2.49 |  |
| TR22040\|c0_g1_i1 | 1.10E-004 | -2.49 |  |
| TR14288\|c0_g1_i3 | 0.00E+000 | -2.49 | mfs agza xanthine uracil permease |
| TR16725\|c0_g1_i1 | 0.00E+000 | -2.49 |  |
| TR1255\|c0_g1_i1 | 0.00E+000 | -2.48 | mop family transporter: multidrug efflux |
| TR1532\|c0_g1_i1 | 4.10E-008 | -2.48 | aureochrome1-like protein |
| TR7478\|c2_g2_i8 | 0.00E+000 | -2.48 | protein |
| TR19424\|c0_g1_i1 | 6.13E-009 | -2.48 |  |
| TR14773\|c0_g1_i3 | 0.00E+000 | -2.48 |  |
| TR18587\|c0_g1_i1 | 0.00E+000 | -2.48 |  |
| TR16025\|c0_g1_i12 | 0.00E+000 | -2.48 | alpha beta hydrolase domain-containing protein 14b |
| TR20613\|c1_g1_i4 | 0.00E+000 | -2.47 | auxin efflux carrier family protein |
| TR5464\|c0_g1_i1 | 0.00E+000 | -2.47 |  |
| TR2268\|c4_g1_i5 | 0.00E+000 | -2.47 | transmembrane protein with metallophosphoesterase domain |
| TR19538\|c0_g1_i1 | 7.82E-008 | -2.47 | hypothetical protein THAOC_13120 |
| TR583\|c1_g1_i1 | 3.13E-012 | -2.47 |  |
| TR21106\|c3_g2_i2 | 3.53E-008 | -2.47 | protein |
| TR24688\|c0_g1_i1 | 1.64E-003 | -2.47 |  |
| TR2268\|c4_g1_i1 | 0.00E+000 | -2.46 | transmembrane protein with metallophosphoesterase domain |
| TR18937\|c0_g1_i5 | 1.25E-004 | -2.46 | protein |
| TR11109\|c0_g1_i1 | 0.00E+000 | -2.46 | protein |
| TR20122\|c1_g2_i12 | 0.00E+000 | -2.46 |  |
| TR2277\|c0_g1_i1 | 0.00E+000 | -2.46 | protein |
| TR15169\|c0_g1_i1 | 0.00E+000 | -2.46 |  |
| TR24490\|c0_g1_i1 | 3.84E-004 | -2.46 |  |
| TR21246\|c0_g1_i1 | 5.77E-006 | -2.46 |  |
| TR9215\|c0_g1_i1 | 0.00E+000 | -2.46 |  |
| TR2268\|c3_g1_i4 | 1.88E-003 | -2.45 |  |
| TR14353\|c0_g1_i2 | 1.15E-011 | -2.45 | delta -sterol-c5 -desaturase 1-like |
| TR462\|c0_g1_i4 | 0.00E+000 | -2.45 |  |
| TR5571\|c0_g1_i2 | 6.47E-007 | -2.45 |  |
| TR9308\|c0_g1_i1 | 0.00E+000 | -2.44 |  |
| TR1284\|c0_g2_i3 | 0.00E+000 | -2.43 |  |
| TR4869\|c1_g3_i7 | 0.00E+000 | -2.43 |  |
| TR3055\|c3_g1_i1 | 0.00E+000 | -2.43 |  |
| TR16106\|c1_g2_i1 | 7.72E-008 | -2.43 | protein |
| TR16925\|c0_g1_i1 | 0.00E+000 | -2.43 |  |
| TR4554\|c0_g2_i1 | 1.93E-003 | -2.42 |  |
| TR926\|c0_g2_i1 | 0.00E+000 | -2.42 |  |
| TR19760\|c0_g1_i1 | 0.00E+000 | -2.42 | translation initiation factor sui1 |
| TR24143\|c0_g1_i3 | 3.95E-004 | -2.42 |  |
| TR10505\|c1_g1_i1 | 6.31E-007 | -2.41 |  |
| TR20939\|c0_g2_i1 | 1.89E-012 | -2.41 |  |
| TR5544\|c0_g1_i2 | 0.00E+000 | -2.41 |  |
| TR6154\|c0_g1_i1 | 8.03E-005 | -2.41 |  |
| TR24246\|c0_g1_i1 | 1.09E-004 | -2.41 | protein |
| TR20534\|c0_g1_i1 | 3.03E-006 | -2.40 | protein |
| TR9280\|c0_g1_i1 | 8.43E-012 | -2.40 |  |
| TR16827\|c0_g1_i4 | 1.87E-008 | -2.40 |  |
| TR6638\|c1_g1_i7 | 4.51E-003 | -2.40 | protein |
| TR25226\|c0_g1_i1 | 0.00E+000 | -2.40 |  |
| TR2099\|c0_g1_i3 | 6.68E-004 | -2.40 |  |
| TR3755\|c4_g3_i3 | 1.29E-003 | -2.39 |  |
| TR3611\|c0_g2_i1 | 0.00E+000 | -2.39 |  |
| TR15545\|c0_g1_i1 | 0.00E+000 | -2.39 |  |
| TR19495\|c0_g1_i1 | 0.00E+000 | -2.39 | atp dependent dna ligase |
| TR18626\|c1_g1_i3 | 7.06E-003 | -2.39 | single-stranded dna-bindig protein mitochondrial |
| TR11661\|c0_g1_i1 | 0.00E+000 | -2.38 |  |
| TR7478\|c2_g2_i4 | 0.00E+000 | -2.38 | protein |
| TR4853\|c1_g1_i6 | 0.00E+000 | -2.38 | protein |
| TR14408\|c0_g1_i1 | 0.00E+000 | -2.38 |  |
| TR16029\|c0_g3_i2 | 0.00E+000 | -2.38 | quinone oxidoreductase |
| TR1284\|c0_g2_i4 | 0.00E+000 | -2.38 |  |
| TR8594\|c0_g1_i2 | 7.84E-009 | -2.38 |  |
| TR11838\|c0_g2_i2 | 6.79E-003 | -2.38 | egf domain-specific o-linked n-acetylglucosamine transferase |
| TR3427\|c0_g1_i1 | 0.00E+000 | -2.37 | serine hydroxymethyltransferase |
| TR7144\|c5_g1_i4 | 1.50E-004 | -2.37 |  |
| TR22066\|c0_g1_i1 | 0.00E+000 | -2.37 |  |
| TR5517\|c0_g1_i1 | 0.00E+000 | -2.37 |  |
| TR4565\|c0_g1_i2 | 5.35E-004 | -2.37 |  |
| TR16045\|c0_g2_i2 | 0.00E+000 | -2.36 |  |
| TR1818\|c0_g1_i1 | 0.00E+000 | -2.36 |  |
| TR22853\|c0_g2_i2 | 0.00E+000 | -2.36 | ubiquitin carboxyl-terminal hydrolase 25 |
| TR18875\|c0_g1_i1 | 0.00E+000 | -2.35 |  |
| TR4173\|c0_g1_i1 | 0.00E+000 | -2.35 |  |
| TR13656\|c0_g1_i3 | 8.59E-003 | -2.35 |  |
| TR24278\|c0_g1_i1 | 5.76E-003 | -2.35 |  |
| TR25649\|c0_g1_i1 | 4.27E-014 | -2.35 |  |
| TR16699\|c0_g2_i1 | 7.86E-008 | -2.35 |  |
| TR11407\|c0_g1_i2 | 0.00E+000 | -2.35 |  |
| TR24604\|c0_g1_i3 | 0.00E+000 | -2.34 |  |
| TR19367\|c0_g1_i1 | 0.00E+000 | -2.34 |  |
| TR18500\|c0_g1_i1 | 0.00E+000 | -2.34 |  |
| TR14466\|c0_g1_i2 | 0.00E+000 | -2.34 |  |
| TR24786\|c0_g1_i1 | 0.00E+000 | -2.34 |  |
| TR16662\|c0_g1_i1 | 0.00E+000 | -2.34 |  |
| TR3076\|c0_g1_i2 | 7.21E-003 | -2.34 |  |
| TR13692\|c0_g1_i2 | 3.89E-004 | -2.34 | cyclin-l1-1 |
| TR2129\|c1_g1_i2 | 3.47E-004 | -2.33 |  |
| TR10430\|c1_g1_i3 | 2.23E-003 | -2.33 |  |
| TR24935\|c0_g1_i1 | 1.35E-003 | -2.33 |  |
| TR12663\|c0_g1_i3 | 0.00E+000 | -2.32 |  |
| TR14404\|c0_g1_i1 | 0.00E+000 | -2.32 | zinc c3hc4 type (ring finger) domain-containing protein |
| TR11312\|c6_g1_i2 | 4.42E-004 | -2.32 |  |
| TR18143\|c0_g1_i1 | 0.00E+000 | -2.32 |  |
| TR16881\|c0_g1_i1 | 0.00E+000 | -2.32 | flagellar calcium-binding protein |
| TR20619\|c0_g1_i5 | 0.00E+000 | -2.31 |  |
| TR17688\|c0_g1_i1 | 0.00E+000 | -2.31 | interferon-induced gtp-binding protein mx |
| TR22158\|c0_g1_i1 | 9.27E-004 | -2.31 |  |
| TR23223\|c4_g2_i3 | 5.90E-003 | -2.31 |  |
| TR24221\|c0_g1_i1 | 0.00E+000 | -2.31 | coq5 family methyltransferase |
| TR1194\|c0_g1_i1 | 0.00E+000 | -2.30 | predicted protein |
| TR16021\|c1_g1_i1 | 2.10E-006 | -2.30 |  |
| TR1297\|c1_g1_i1 | 9.35E-014 | -2.30 |  |
| TR10499\|c1_g1_i6 | 8.41E-013 | -2.30 | cry-dash from the cryptochrome photolyase family |
| TR24955\|c0_g2_i4 | 4.22E-007 | -2.30 | protein |
| TR20414\|c0_g1_i1 | 2.34E-003 | -2.30 |  |
| TR11564\|c0_g1_i1 | 3.51E-004 | -2.30 |  |
| TR20222\|c0_g2_i1 | 0.00E+000 | -2.29 | nadph-cytochrome p450 |
| TR10062\|c0_g1_i1 | 0.00E+000 | -2.29 |  |
| TR5173\|c0_g1_i1 | 2.95E-004 | -2.29 |  |
| TR7262\|c0_g1_i1 | 1.81E-008 | -2.29 |  |
| TR10042\|c0_g1_i1 | 0.00E+000 | -2.29 |  |
| TR2606\|c0_g1_i1 | 1.10E-008 | -2.29 | coiled-coil domain-containing protein 25 |
| TR21150\|c2_g1_i5 | 0.00E+000 | -2.29 | abc transporter |
| TR21939\|c0_g1_i1 | 1.75E-009 | -2.29 |  |
| TR11087\|c0_g1_i1 | 0.00E+000 | -2.29 | non-symbiotic hemoglobin partial |
| TR15879\|c0_g1_i3 | 7.46E-014 | -2.29 |  |
| TR257\|c0_g1_i1 | 0.00E+000 | -2.28 | patatin family protein |
| TR7424\|c3_g1_i1 | 2.80E-005 | -2.28 |  |
| TR18085\|c0_g1_i2 | 0.00E+000 | -2.28 | small ubiquitin-like modifier |
| TR2310\|c0_g1_i1 | 0.00E+000 | -2.28 |  |
| TR18408\|c0_g1_i3 | 0.00E+000 | -2.27 | 2og-fe oxygenase family protein |
| TR5346\|c0_g1_i2 | 0.00E+000 | -2.27 |  |
| TR18818\|c0_g1_i1 | 1.21E-011 | -2.27 |  |
| TR16051\|c0_g1_i3 | 0.00E+000 | -2.27 | pyruvate dehydrogenase (acetyl-transferring) mitochondrial-like |
| TR3636\|c0_g1_i1 | 0.00E+000 | -2.27 |  |
| TR6655\|c0_g1_i4 | 0.00E+000 | -2.27 | protein |
| TR9832\|c0_g2_i1 | 0.00E+000 | -2.27 | zinc induced facilitator-like 1 isoform 2 |
| TR13466\|c0_g1_i2 | 3.26E-003 | -2.27 | calcium-binding mitochondrial carrier protein aralar1 |
| TR25429\|c0_g1_i1 | 0.00E+000 | -2.27 |  |
| TR14408\|c0_g2_i1 | 1.06E-003 | -2.27 |  |
| TR18321\|c1_g1_i1 | 0.00E+000 | -2.26 |  |
| TR15046\|c0_g1_i1 | 2.64E-012 | -2.26 |  |
| TR21150\|c2_g1_i3 | 0.00E+000 | -2.26 | abc transporter |
| TR8424\|c0_g1_i1 | 0.00E+000 | -2.26 |  |
| TR19725\|c0_g1_i1 | 0.00E+000 | -2.26 |  |
| TR16938\|c0_g1_i1 | 0.00E+000 | -2.25 |  |
| TR20278\|c0_g2_i2 | 1.18E-006 | -2.25 | serine hydroxymethyltransferase |
| TR4561\|c0_g1_i2 | 0.00E+000 | -2.25 | malate synthase a |
| TR2245\|c3_g2_i1 | 6.90E-008 | -2.25 | protein |
| TR25005\|c0_g1_i3 | 0.00E+000 | -2.25 |  |
| TR7513\|c3_g2_i1 | 0.00E+000 | -2.24 |  |
| TR12255\|c0_g1_i1 | 2.63E-009 | -2.24 |  |
| TR12592\|c0_g1_i2 | 6.82E-013 | -2.24 |  |
| TR9667\|c0_g1_i1 | 3.99E-005 | -2.24 |  |
| TR23259\|c1_g1_i1 | 0.00E+000 | -2.24 | fadh- -dienoyl- reductase |
| TR508\|c1_g1_i1 | 5.65E-003 | -2.23 |  |
| TR2315\|c10_g1_i1 | 0.00E+000 | -2.23 |  |
| TR11432\|c0_g1_i1 | 0.00E+000 | -2.22 |  |
| TR16058\|c2_g1_i1 | 0.00E+000 | -2.22 | probable lrr receptor-like serine threonine-protein kinase at1g34110-like |
| TR16437\|c0_g1_i1 | 0.00E+000 | -2.22 | patatin-like phospholipase domain-containing protein 7 |
| TR24806\|c0_g2_i1 | 1.38E-008 | -2.22 | iron-sulfur cluster assembly protein |
| TR18690\|c0_g1_i1 | 0.00E+000 | -2.22 |  |
| TR2268\|c5_g1_i1 | 0.00E+000 | -2.22 |  |
| TR24998\|c0_g1_i1 | 0.00E+000 | -2.22 |  |
| TR2272\|c2_g4_i3 | 0.00E+000 | -2.21 |  |
| TR853\|c0_g1_i1 | 5.01E-012 | -2.21 |  |
| TR25050\|c0_g1_i1 | 9.03E-004 | -2.21 |  |
| TR12538\|c0_g1_i1 | 0.00E+000 | -2.21 | serine-type peptidase |
| TR1602\|c0_g1_i2 | 2.01E-004 | -2.21 |  |
| TR15257\|c0_g1_i1 | 0.00E+000 | -2.21 |  |
| TR8326\|c0_g1_i1 | 2.37E-010 | -2.21 |  |
| TR271\|c0_g1_i1 | 4.44E-016 | -2.21 | sugar kinase and ribulose phosphate 3 fused |
| TR1533\|c0_g1_i1 | 2.15E-003 | -2.20 | ck1 ck1 ck1-d protein variant |
| TR1263\|c0_g1_i1 | 0.00E+000 | -2.20 | pyridoxamine 5 phosphate oxidase-related protein |
| TR20525\|c0_g1_i1 | 0.00E+000 | -2.20 |  |
| TR3727\|c6_g2_i2 | 4.03E-003 | -2.20 |  |
| TR12592\|c0_g1_i4 | 0.00E+000 | -2.20 |  |
| TR19705\|c0_g1_i1 | 0.00E+000 | -2.20 |  |
| TR8431\|c0_g1_i1 | 0.00E+000 | -2.20 |  |
| TR24788\|c0_g1_i1 | 3.07E-003 | -2.20 |  |
| TR15317\|c0_g2_i1 | 0.00E+000 | -2.19 | estradiol 17-beta-dehydrogenase 8-like |
| TR11591\|c0_g1_i1 | 0.00E+000 | -2.19 | mate efflux family protein |
| TR15814\|c0_g1_i1 | 0.00E+000 | -2.19 |  |
| TR24391\|c0_g1_i1 | 0.00E+000 | -2.19 |  |
| TR24266\|c0_g2_i1 | 5.33E-015 | -2.19 |  |
| TR668\|c0_g1_i1 | 0.00E+000 | -2.19 |  |
| TR15224\|c0_g1_i1 | 0.00E+000 | -2.19 | serine threonine protein phosphatase 2b catalytic subunit gamma |
| TR11309\|c0_g1_i1 | 0.00E+000 | -2.18 |  |
| TR21143\|c1_g1_i6 | 9.85E-004 | -2.18 |  |
| TR1265\|c0_g1_i1 | 0.00E+000 | -2.18 | partial |
| TR20121\|c0_g1_i3 | 0.00E+000 | -2.18 |  |
| TR14400\|c0_g1_i1 | 0.00E+000 | -2.18 |  |
| TR8271\|c0_g1_i2 | 2.22E-016 | -2.18 |  |
| TR19449\|c0_g1_i1 | 0.00E+000 | -2.18 | glycerophosphodiester phosphodiesterase |
| TR7218\|c3_g2_i1 | 0.00E+000 | -2.18 |  |
| TR6591\|c1_g1_i2 | 8.15E-004 | -2.18 | protein |
| TR19173\|c0_g1_i1 | 0.00E+000 | -2.18 | inosine monophosphate dehydrogenase |
| TR7478\|c2_g2_i3 | 0.00E+000 | -2.17 | protein |
| TR7369\|c0_g1_i1 | 2.68E-006 | -2.17 | geranylgeranyl pyrophosphate synthase |
| TR10436\|c1_g1_i5 | 1.72E-004 | -2.17 |  |
| TR4316\|c0_g1_i1 | 0.00E+000 | -2.17 |  |
| TR7934\|c0_g1_i1 | 0.00E+000 | -2.17 |  |
| TR22421\|c0_g1_i1 | 3.83E-005 | -2.17 |  |
| TR18546\|c0_g1_i1 | 0.00E+000 | -2.17 | threonine-trna ligase |
| TR12118\|c0_g1_i1 | 2.01E-004 | -2.17 |  |
| TR7513\|c3_g2_i5 | 3.20E-014 | -2.17 |  |
| TR2323\|c0_g1_i3 | 0.00E+000 | -2.17 |  |
| TR4204\|c0_g3_i1 | 4.38E-006 | -2.17 | syntaxin-like protein |
| TR15654\|c0_g1_i1 | 1.75E-013 | -2.17 |  |
| TR462\|c0_g1_i3 | 0.00E+000 | -2.17 |  |
| TR18370\|c0_g3_i1 | 0.00E+000 | -2.16 |  |
| TR19418\|c0_g1_i1 | 0.00E+000 | -2.16 |  |
| TR25114\|c0_g1_i1 | 0.00E+000 | -2.16 | u2 snrnp component ist3 |
| TR11356\|c2_g1_i2 | 9.59E-003 | -2.16 |  |
| TR7959\|c0_g1_i1 | 0.00E+000 | -2.16 |  |
| TR19416\|c0_g1_i1 | 0.00E+000 | -2.16 | chr family transporter: chromate ion |
| TR22897\|c0_g1_i1 | 7.68E-009 | -2.15 |  |
| TR25005\|c0_g1_i1 | 0.00E+000 | -2.15 | elongation of very long chain fatty acids protein 2 |
| TR13681\|c0_g1_i4 | 1.73E-006 | -2.15 |  |
| TR2237\|c0_g1_i1 | 1.43E-004 | -2.15 | phosphopantothenoylcysteine decarboxylase |
| TR19742\|c0_g1_i1 | 0.00E+000 | -2.15 |  |
| TR17519\|c0_g1_i1 | 0.00E+000 | -2.15 |  |
| TR11310\|c0_g1_i1 | 0.00E+000 | -2.14 |  |
| TR4350\|c0_g2_i3 | 0.00E+000 | -2.14 |  |
| TR15823\|c0_g1_i1 | 3.85E-003 | -2.14 |  |
| TR20609\|c0_g1_i2 | 6.24E-004 | -2.14 | myo-inositol-1-phosphate synthase |
| TR14379\|c1_g1_i1 | 3.66E-004 | -2.13 | sarcosine mitochondrial |
| TR3759\|c1_g1_i4 | 4.66E-004 | -2.13 | predicted protein |
| TR101\|c0_g1_i1 | 0.00E+000 | -2.13 |  |
| TR13191\|c0_g1_i1 | 0.00E+000 | -2.13 |  |
| TR3117\|c4_g2_i2 | 8.40E-005 | -2.13 |  |
| TR12563\|c1_g1_i4 | 8.72E-003 | -2.13 |  |
| TR5726\|c0_g1_i1 | 0.00E+000 | -2.13 |  |
| TR4277\|c0_g1_i1 | 0.00E+000 | -2.13 |  |
| TR1628\|c0_g1_i1 | 0.00E+000 | -2.13 | alpha-galactosidase |
| TR19530\|c0_g1_i1 | 0.00E+000 | -2.13 |  |
| TR14437\|c0_g1_i4 | 0.00E+000 | -2.12 | probable adp-ribosylation factor gtpase-activating protein agd5 isoform x2 |
| TR16025\|c0_g1_i5 | 0.00E+000 | -2.12 |  |
| TR6655\|c1_g1_i4 | 5.55E-003 | -2.12 |  |
| TR20262\|c0_g1_i2 | 0.00E+000 | -2.12 | auxin efflux carrier family protein |
| TR25916\|c0_g1_i1 | 0.00E+000 | -2.12 |  |
| TR4653\|c0_g1_i2 | 5.89E-013 | -2.12 |  |
| TR24082\|c0_g1_i1 | 0.00E+000 | -2.12 | alcohol dehydrogenase |
| TR19773\|c0_g1_i1 | 0.00E+000 | -2.12 |  |
| TR12652\|c1_g1_i1 | 0.00E+000 | -2.11 |  |
| TR24221\|c0_g1_i2 | 0.00E+000 | -2.11 | coq5 family methyltransferase |
| TR17919\|c0_g1_i1 | 0.00E+000 | -2.11 |  |
| TR3731\|c0_g2_i1 | 5.13E-003 | -2.11 |  |
| TR5237\|c0_g1_i1 | 0.00E+000 | -2.11 | protein |
| TR14725\|c0_g1_i1 | 0.00E+000 | -2.11 | cystathionine gamma-lyase |
| TR19144\|c0_g1_i1 | 0.00E+000 | -2.11 |  |
| TR17676\|c0_g1_i1 | 0.00E+000 | -2.11 |  |
| TR15859\|c0_g1_i2 | 0.00E+000 | -2.11 |  |
| TR8318\|c1_g1_i1 | 0.00E+000 | -2.11 |  |
| TR20588\|c0_g1_i1 | 0.00E+000 | -2.11 | homebox domain-containing protein |
| TR21865\|c0_g1_i1 | 0.00E+000 | -2.10 | protein |
| TR15255\|c0_g1_i2 | 1.14E-003 | -2.10 |  |
| TR12331\|c0_g1_i1 | 1.95E-007 | -2.10 | related to mynd domain protein |
| TR24964\|c0_g1_i3 | 2.00E-004 | -2.10 |  |
| TR22332\|c0_g1_i1 | 6.35E-012 | -2.10 |  |
| TR16000\|c0_g1_i1 | 0.00E+000 | -2.10 |  |
| TR24357\|c0_g1_i2 | 3.34E-003 | -2.10 | sh3 domain-containing protein |
| TR19406\|c0_g1_i3 | 2.09E-005 | -2.10 |  |
| TR24229\|c0_g1_i1 | 0.00E+000 | -2.10 | ring zinc finger-containing protein |
| TR2015\|c0_g1_i1 | 0.00E+000 | -2.09 |  |
| TR10402\|c0_g1_i4 | 0.00E+000 | -2.09 |  |
| TR3498\|c0_g1_i1 | 4.21E-006 | -2.09 |  |
| TR11407\|c0_g1_i1 | 2.27E-013 | -2.09 |  |
| TR21150\|c0_g1_i3 | 0.00E+000 | -2.08 | abc transporter |
| TR10542\|c0_g3_i1 | 0.00E+000 | -2.08 | protein |
| TR12567\|c0_g1_i3 | 0.00E+000 | -2.08 | arylamine n-acetyltransferase 2 |
| TR16080\|c0_g1_i1 | 0.00E+000 | -2.08 | rna-editing complex protein mp100 |
| TR21150\|c0_g1_i1 | 0.00E+000 | -2.08 | abc transporter |
| TR18004\|c0_g1_i1 | 1.42E-003 | -2.08 |  |
| TR21076\|c1_g3_i1 | 9.31E-008 | -2.08 |  |
| TR9691\|c0_g2_i1 | 1.30E-004 | -2.08 |  |
| TR15268\|c0_g1_i2 | 2.92E-006 | -2.08 |  |
| TR22296\|c0_g2_i1 | 2.90E-003 | -2.07 | mfs general substrate transporter |
| TR15515\|c0_g1_i1 | 0.00E+000 | -2.07 |  |
| TR4791\|c0_g3_i1 | 2.12E-007 | -2.07 |  |
| TR3543\|c0_g1_i1 | 0.00E+000 | -2.07 | -specific serine endopeptidase |
| TR10454\|c4_g2_i1 | 0.00E+000 | -2.07 | protein |
| TR10623\|c0_g1_i1 | 2.39E-012 | -2.07 |  |
| TR23740\|c0_g2_i1 | 2.72E-003 | -2.06 | death-associated protein kinase 1 |
| TR10473\|c1_g2_i1 | 0.00E+000 | -2.06 |  |
| TR14354\|c0_g1_i1 | 0.00E+000 | -2.06 | phosphatidylinositol- -trisphosphate 3- |
| TR8369\|c0_g1_i1 | 0.00E+000 | -2.05 | beta-ig-h3 fasciclin |
| TR21076\|c2_g1_i6 | 0.00E+000 | -2.05 | protein |
| TR8270\|c0_g1_i1 | 3.37E-011 | -2.05 |  |
| TR6591\|c1_g1_i1 | 1.97E-004 | -2.05 | protein |
| TR16029\|c0_g2_i1 | 0.00E+000 | -2.05 | protein rad-8 |
| TR1720\|c0_g1_i1 | 0.00E+000 | -2.05 |  |
| TR12553\|c0_g1_i1 | 0.00E+000 | -2.05 |  |
| TR20069\|c1_g1_i1 | 0.00E+000 | -2.04 | thymidylate synthase |
| TR9576\|c0_g2_i1 | 1.84E-005 | -2.04 |  |
| TR19917\|c1_g1_i3 | 1.76E-003 | -2.04 |  |
| TR4392\|c0_g1_i2 | 0.00E+000 | -2.04 | dnaj homolog subfamily b member 9 |
| TR12663\|c0_g1_i1 | 0.00E+000 | -2.04 |  |
| TR21076\|c1_g2_i1 | 1.41E-003 | -2.04 |  |
| TR8372\|c0_g1_i1 | 0.00E+000 | -2.04 | tkl protein kinase |
| TR11899\|c0_g1_i1 | 0.00E+000 | -2.04 |  |
| TR25128\|c0_g1_i1 | 4.36E-010 | -2.04 | major facilitator superfamily |
| TR21297\|c0_g1_i1 | 4.02E-006 | -2.04 | protein |
| TR23304\|c0_g1_i2 | 1.62E-003 | -2.03 |  |
| TR8379\|c0_g1_i2 | 3.62E-008 | -2.03 | potassium voltage-gated channel subfamily h member 8-like |
| TR506\|c0_g2_i2 | 1.67E-008 | -2.03 | predicted protein |
| TR20420\|c3_g4_i1 | 5.25E-007 | -2.03 |  |
| TR6698\|c0_g1_i2 | 0.00E+000 | -2.03 | fructose- -bisphosphatase precursor |
| TR17663\|c0_g1_i3 | 0.00E+000 | -2.03 | agc pkg protein kinase |
| TR21958\|c0_g1_i1 | 0.00E+000 | -2.03 | inositol hexakisphosphate and diphosphoinositol-pentakisphosphate |
| TR14408\|c0_g1_i2 | 3.05E-009 | -2.03 |  |
| TR24280\|c0_g1_i1 | 1.17E-003 | -2.03 | cca trna nucleotidyltransferase 2 |
| TR15057\|c0_g1_i1 | 0.00E+000 | -2.03 | protein |
| TR19700\|c0_g1_i1 | 6.42E-007 | -2.03 |  |
| TR20131\|c1_g1_i6 | 0.00E+000 | -2.03 | nde1p |
| TR21106\|c3_g1_i5 | 3.45E-014 | -2.03 | protein |
| TR24849\|c0_g1_i1 | 0.00E+000 | -2.03 |  |
| TR535\|c0_g1_i2 | 0.00E+000 | -2.02 |  |
| TR14771\|c3_g3_i1 | 5.65E-003 | -2.02 |  |
| TR535\|c0_g1_i4 | 0.00E+000 | -2.02 |  |
| TR10405\|c0_g1_i1 | 0.00E+000 | -2.02 | cmp-sialic acid transporter 5-like |
| TR23223\|c4_g1_i18 | 5.41E-004 | -2.01 |  |
| TR7187\|c2_g1_i1 | 0.00E+000 | -2.01 | protein |
| TR1676\|c0_g1_i1 | 3.24E-008 | -2.01 | electron carrier protein |
| TR22045\|c0_g1_i2 | 0.00E+000 | -2.01 |  |
| TR11370\|c4_g1_i2 | 5.60E-004 | -2.01 |  |
| TR8980\|c0_g1_i2 | 2.98E-003 | -2.01 |  |
| TR3055\|c3_g1_i2 | 0.00E+000 | -2.01 |  |
| TR10933\|c0_g1_i3 | 1.41E-003 | -2.01 |  |
| TR10454\|c4_g3_i1 | 4.44E-016 | -2.01 | protein |
| TR16025\|c0_g1_i9 | 0.00E+000 | -2.01 | alpha beta hydrolase domain-containing protein 14a isoform x2 |
| TR17455\|c0_g1_i1 | 2.66E-006 | -2.00 |  |
| TR1842\|c0_g1_i1 | 0.00E+000 | -2.00 | 6-hydroxy-d-nicotine oxidase |
| TR1491\|c0_g1_i1 | 0.00E+000 | -2.00 | protein |
| TR19079\|c0_g1_i1 | 0.00E+000 | -2.00 |  |
| TR7126\|c0_g2_i1 | 1.48E-004 | -2.00 | rubredoxin domain protein |
| TR11508\|c0_g1_i1 | 0.00E+000 | -2.00 | mitochondrial phosphate carrier protein |

**Supplementary Table 2.** The table reports selected reference genes and genes of interest, their abbreviation (Abbr) and function.

| Reference genes (RGs) | |  |
| --- | --- | --- |
| Abbr | Gene name | Function |
| Act | Actin | Formation of the cytoskeleton |
| CaM | Calmodulin | Calcium-binding messenger |
| EFL | Translation elongation factor-like protein | Translational elongation factor |
| GAPDH | Glyceraldehyde 3-Phosphate Dehydrogenase | Oxidoreductase in glycolysis and gluconeogenesis |
| Tub-α | α-tubulin | Microtubule subunit |
| Tub-β | β-tubulin | Microtubule subunit |
| Genes of Interest (GOI) | |  |
| PIGA | Phosphatidylinositol N-acetylglucosaminyltransferase subunit A | phosphatidylinositol synthesis |
| PIK3C3 | Phosphatidylinositol 3-kinase catalytic subunit type 3 | phosphatidylinositol synthesis |
| PtdIns(3) | Phosphatidylinositol-3-Phosphatase | phosphatidylinositol synthesis |
| MGD | Monogalactosyldiacylglycerol synthase | monogalactosyldiacylglycerol synthesis |
| VDE | Violaxanthin De-Epoxidase | violaxanthin metabolism |
| Other selected genes for transcriptome validation | |  |
| ABC | ATP binding cassette transporter | Transport of several compounds. It is also involved in the involved in multiple drug resistance. |
| AG | Adenylate and Guanylate cyclase catalytic domain-containing protein | Involved in phosphorus-oxygen lyase activity, intracellular signal transduction and cyclic nucleotide biosynthetic process |
| NRT | Nitrate Transporter | Involved in the transport of nitrate |
| PDEase | 3'5'-cyclic nucleotide phosphodiesterase | Involved in signal transduction and 3',5'-cyclic-nucleotide phosphodiesterase activity |
| Rsu | rRNA pseudouridylate synthase | Involved in Ribosome Biogenesis |
| TE | Transposon Protein | DNA sequences which have the ability to move and spread within the genome |
| VIT | Vacuolar Iron Family Transporter | Transport of iron ions from the inter-membrane space into the stroma or matrix |

**Supplementary Table 3.** The table reports selected reference genes and genes of interest, their primer sequences, amplicon sizes (bp), % oligo efficiencies (E) and correlation factors (R^2^).

| Reference Genes | Forward Primer | Reverse Primer | bp | E | R^2^ |
| --- | --- | --- | --- | --- | --- |
| Act | CCGTCAGCCTGGTATCATGG | CTCGGTAAGGAGGACAGGGT | 208 | 100 | 0.9991 |
| CaM | GGTCATGAGAAAGCTAGGCCA | CAATCGACCCACTTCCGTCA | 200 | 99.5 | 0..9947 |
| EFL | CAATTGTCGATGCCCCAGGA | CACGCCAAGCAAATACAGCA | 197 | 93 | 0.9932 |
| GAPDH | TGAACGGCAAGCTTACTGGT | GATTCCGGCCTTCTCGTCAA | 233 | 100 | 0.9975 |
| Tub-α | TGCCGACAACTGTACAGGTC | CAAGAGGGCATGGGTGGAAA | 211 | 100 | 0.9876 |
| Tub-β | GCTACACTTTCCGTCCACCA | TGTTGACTGCCAGCTTTCGA | 217 | 100 | 0.9938 |
| Genes of Interest | Forward Primer | Reverse Primer | bp | E | R^2^ |
| PIGA | AAGATGCGCTCCCTACTGTC | ATACACTCGGCAACCCCATT | 154 | 100 | 0.9998 |
| PIK3C3 | TGGTGATGGGAGCTCTAGGA | GAGACAGAGATCCAACGACG | 155 | 100 | 0.9909 |
| PtdIns(3) | GGAACAAACGGGCATTCTCC | GGGCTTCTGAACCACCGTAT | 224 | 100 | 0.9808 |
| MGD | GATGTGGTCGTCAGTGTGCA | CATCGTTTCCATCCCATTCG | 158 | 100 | 0.9756 |
| VDE | ACTCTTGTATTACCGGCTGCA | AACCCCGCAACTTTGTACCA | 211 | 100 | 0.9913 |
| Others | Forward Primer | Reverse Primer | bp | E | R^2^ |
| ABC | TGCTCTGGTTGGTTCCGTAA | CGAGACCAACACCAACACTG | 242 | 100 | 0.9951 |
| AG | TCGATAAGCAAGCGTACCGG | AAGTGTCAGGACCAAGAGCG | 189 | 100 | 0.9843 |
| NRT | CCTGCACCGGATTTGTCAAC | ACAACGCTGATCCCATGACA | 204 | 100 | 0.9973 |
| PDEase | TCCAAACGCTCAGCTTGTGA | GTTCTTCCGAAGTGCCTGGA | 238 | 100 | 0.9859 |
| Rsu | TCGAAGCAACCCAAGAGTGT | GCAATTCCATTTGTCGAGCGA | 221 | 100 | 0.9949 |
| TE | GCAAGGGTCGTTCAAGGGTA | GTTCGAACTGCTGCTCCTCA | 233 | 100 | 0.9979 |
| VIT | CGACTTGATGATGACCCAGGA | CATGCCGCCTTGAACAACG | 189 | 100 | 0.9843 |
